# Mechanics-informed risk-aware learning for multiaxial structural reliability with Bayesian calibration

**DOI:** 10.1038/s44172-026-00752-y

**Published:** 2026-08-14

**Authors:** Gaofeng Zhang, Xuanrui Yu, Anxiang Song, Hanguang Liu, Xinhong Lin, Xuyijin Zhang, Qianling Wang, Wentao Wang, Pandeng Zhang

**Affiliations:** 1 Sanyou Future (Chongqing) Intelligence Automotive Chassis Technology Co., Ltd., Chongqing, China; 2 School of Civil and Hydraulic Engineering, Chongqing University of Science and Technology, Chongqing, Shapingba District China; 3 School of Civil Engineering, Chongqing Sanxia University of Science and Technology, Chongqing, Wanzhou China; 4 Xiamen University of Technology, Xiamen, Fujian China; 5 Department of Applied Mathematics, Universitat Politècnica de Catalunya-BarcelonaTech, Barcelona, Spain

**Keywords:** Mechanical engineering, Computer science

## Abstract

Reliable prediction of structural vulnerability under complex multiaxial loading remains challenging due to nonlinear load couplings, imbalanced failure-critical samples, and limited model interpretability. Here, we present a mechanics-informed, risk-aware learning framework integrating polynomial–harmonic feature augmentation, Weibull-based risk reweighting, and a unified Degradation Risk Score that combines stress margins and bolt pretension loss. Demonstrated on a bolted steering-knuckle assembly with finite-element-derived multiaxial load–response data, the framework improves multiple linear regression from R² = 0.37 to 0.96 with a 67% reduction in RMSE, while enhancing robustness and sensitivity in high-risk regimes across ensemble and neural network models. SHAP analysis confirms that physically meaningful multiaxial interaction features dominate predictions, revealing critical load paths associated with structural vulnerability. Finally, Bayesian logistic and Weibull calibration provide a route to link Degradation Risk Score to component-level failure probabilities, enabling probabilistic risk assessment and reliability-centred decision-making in practical engineering systems.

## Abstract (web summary)

Gaofeng Zhang and colleagues develop a mechanics-informed, risk-aware learning framework for multiaxial structural reliability, combining augmented load features, Weibull reweighting and a Degradation Risk Score. In a bolted steering-knuckle assembly, it raises linear-model R² from 0.37 to 0.96, reduces RMSE by 67%, and enables Bayesian risk calibration.

## Introduction

Complex multiaxial loading conditions are inherent to numerous safety-critical structural systems, such as automotive steering knuckles, aircraft wing–fuselage joints, offshore wind turbine foundations, and railway bogie frames^1–4^^.^ These components are often located along key load-transfer paths and are subjected to spatially varying forces and moments during service. Their failure may compromise system-level safety, durability, and operational continuity, leading to severe economic and engineering consequences^5,6^. Accurate, robust, and interpretable prediction of structural vulnerability under such loading conditions is therefore essential for reliability-centered design and maintenance^7–9^.

Physics-based methods, such as finite element analysis (FEA), fatigue-fracture mechanics, and degradation-curve models, remain central to structural reliability assessment because they provide explicit mechanical interpretation of stress evolution, damage progression, and failure thresholds^10–12^. Degradation-curve-based approaches combined with Bayesian updating further allow assumed relationships between degradation indicators and failure probability to be progressively refined when inspection, testing, or monitoring data become available^13–16^. These methods are powerful for probabilistic reliability assessment when the relevant degradation variables and component-level observations are accessible. However, for multiaxial jointed structures, a primary difficulty lies in obtaining mechanically meaningful stress and connection-degradation indicators from nonlinear force–moment interactions, contact behavior, and localized load redistribution.

Machine learning (ML) has emerged as an efficient alternative for mapping high-dimensional inputs to complex structural responses and has shown promise in structural health monitoring, reliability analysis, and performance prediction^17–19^. Nevertheless, its practical use in reliability-critical multiaxial problems remains limited. Existing ML models often rely on raw load or sensor inputs, which may not adequately encode multiaxial coupling, contact-induced stress redistribution, or localized failure modes^20,21^. In addition, degradation datasets are frequently imbalanced, with failure-critical samples occupying only a small fraction of the response space, causing conventional loss functions to underrepresent safety-relevant regimes^22^. Finally, many ML outputs remain difficult to translate into interpretable risk indicators that can support inspection, prioritization, or maintenance decisions.

To address these challenges, we propose a mechanics-informed, risk-aware learning framework for structural reliability prediction under complex multiaxial loading. The framework integrates three components. First, polynomial–harmonic feature augmentation enriches the original force–moment inputs with mechanically meaningful nonlinear and interaction terms. Second, Weibull-based probabilistic reweighting assigns greater learning emphasis to failure-critical samples. Third, a unified Degradation Risk Score (DRS) combines stress-margin degradation and bolt pretension loss into a normalized vulnerability indicator. The framework is demonstrated on a bolted steering-knuckle assembly, a representative multiaxial jointed structure involving both material overstress and connection-level degradation. The resulting workflow improves predictive accuracy, enhances high-risk sensitivity, and provides interpretable risk-oriented outputs. In addition, Bayesian logistic and Weibull calibration formulations are introduced to link DRS with failure probability, extending the framework from deterministic vulnerability scoring toward probabilistic engineering risk assessment. Its model-agnostic workflow can be adapted to analogous multiaxial jointed systems with defined load inputs and degradation mechanisms, thereby bridging mechanics-informed prediction and reliability-oriented decision-making.

## Results

### Framework overview for reliability-oriented prediction

**Fig. 1:**
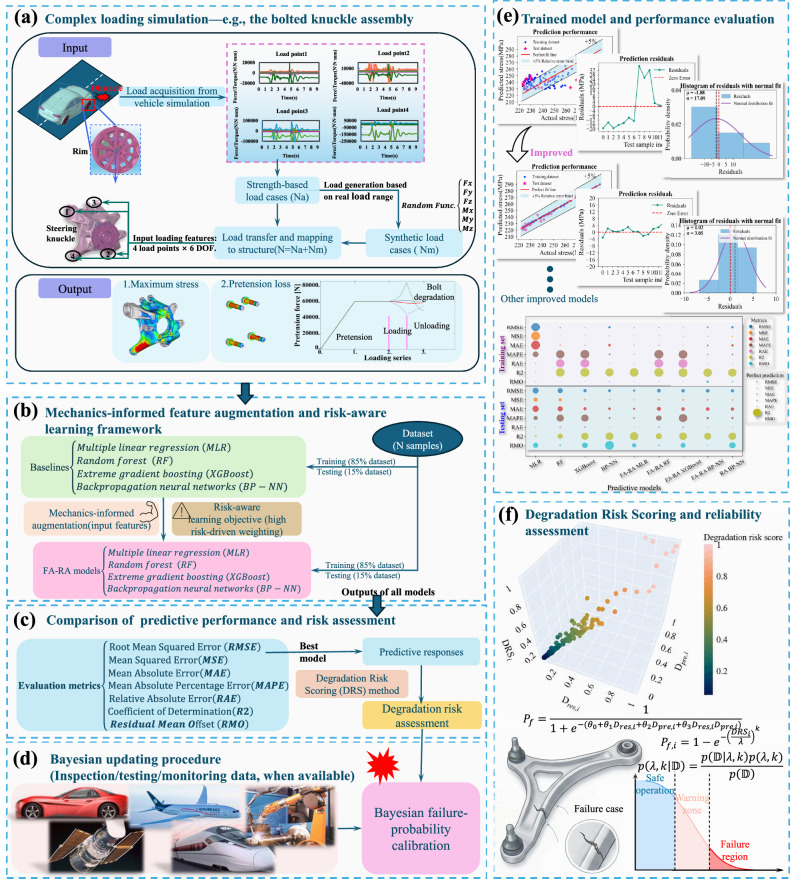
Workflow of the proposed mechanics-informed, risk-aware learning framework for structural reliability prediction under multiaxial loading. **a** Multibody dynamics load acquisition and finite-element simulation, producing critical stress and bolt pretension degradation. **b** Mechanics-informed feature augmentation (FA) and risk-aware (RA) learning framework, showing baseline and FA-RA enhanced models. **c** Predictive performance comparison using seven residual-based evaluation metrics and subsequent DRS-based risk assessment. **d** Bayesian failure-probability calibration, incorporating inspection, testing, or monitoring data when available. **e** Trained model evaluation, showing predicted versus physical responses, residual distributions, and metric benchmarking across models. **f** Integration of DRS with Bayesian probability calibration to quantify sample-wise failure risk and structural reliability under monitoring or operational scenarios.

We propose a mechanics-informed and risk-aware learning workflow for structural reliability prediction under complex multiaxial loading. As summarized in Fig. 1, the framework links multiaxial load generation, mechanics-informed feature construction, risk-aware model training, predictive benchmarking, degradation risk scoring, and Bayesian probability calibration within a unified reliability-oriented pipeline. The workflow is demonstrated on a representative bolted steering-knuckle assembly, where the prediction targets include the critical stress response of the knuckle and bolt-level pretension degradation.

The framework operates in three connected stages. First, multibody dynamics (MBD) simulations and finite-element analysis (FEA) provide physically grounded load–response data under complex service-like multiaxial loading. Second, polynomial–harmonic feature augmentation and Weibull-based risk-aware learning are used to improve nonlinear response representation and increase sensitivity to upper-tail degradation samples. Third, the trained models are evaluated using residual-based metrics, and their predicted responses are integrated into a Degradation Risk Score (DRS) for sample-wise vulnerability ranking. When inspection, testing, or monitoring data are available, DRS or its degradation components can be further linked to component-level failure probability through Bayesian calibration. The technical details of load generation, finite-element modelling, feature construction, risk-aware learning, DRS formulation, and Bayesian updating are provided in the section “*Methods*”.

### Predictive performance across model families

**Fig. 2:**
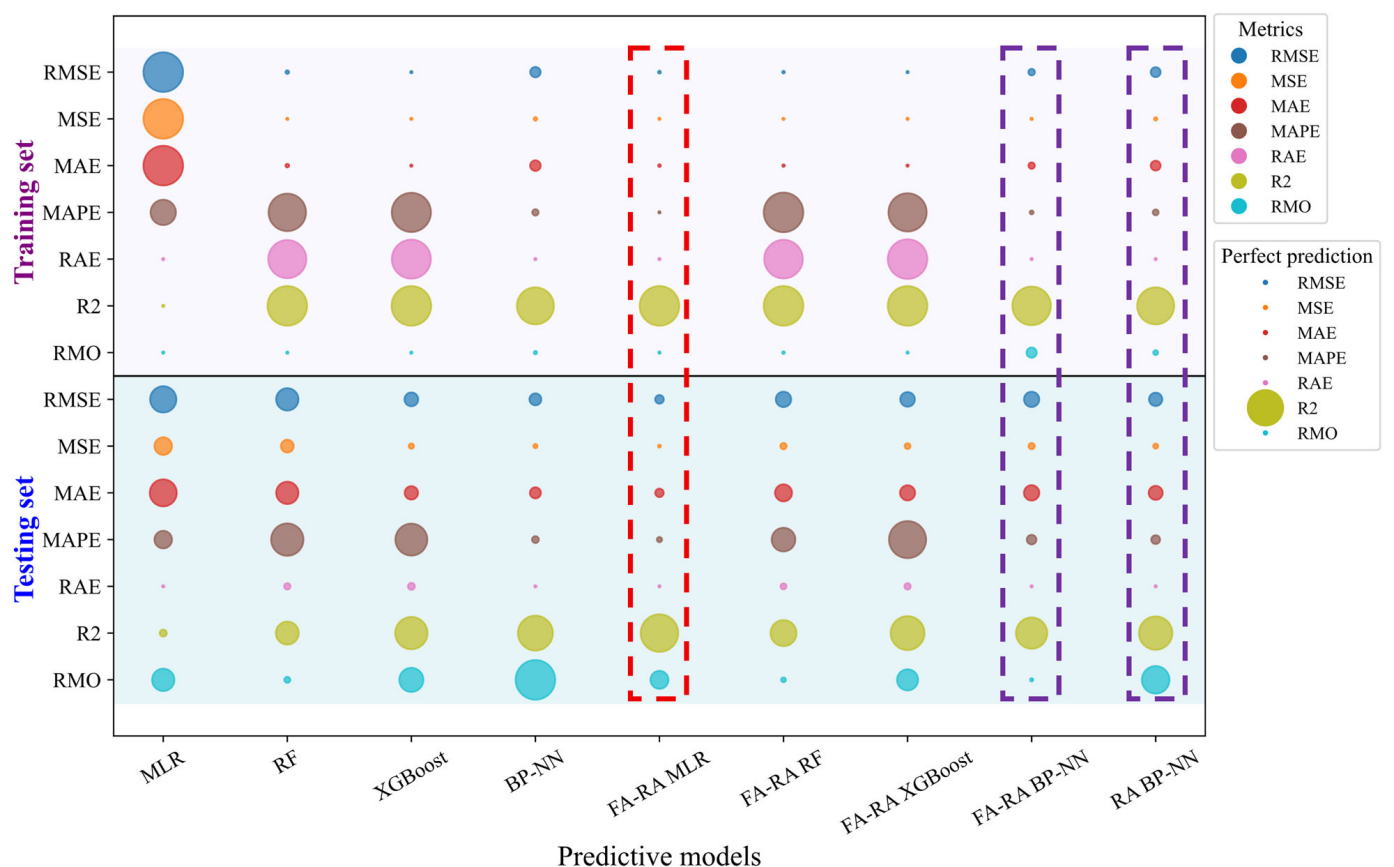
Predictive performance benchmarking across model families. Bubble plot comparing training and testing performance of baseline and FA/RA-enhanced models using RMSE, MSE, MAE, MAPE, RAE, ${R}^{2}$, and residual mean offset (RMO). Bubble size represents the normalized magnitude of each metric. Lower values are preferred for RMSE, MSE, MAE, MAPE, RAE, and residual mean offset, whereas higher values are preferred for ${R}^{2}$.

The predictive performance of all model variants was evaluated using seven metrics on both training and testing sets, with the overall benchmarking results summarized in Fig. 2 and complete testing-set metrics provided in Supplementary Table S1. Among the baseline models, backpropagation neural networks (BP-NN) and XGBoost outperform multiple linear regression (MLR) and random forest (RF) in critical-stress prediction, reflecting their stronger ability to capture nonlinear multiaxial load–response relationships^23,24^. On the testing set, BP-NN achieves the lowest baseline RMSE of 4.49 with ${R}^{2}$ = 0.91, whereas baseline MLR shows substantially larger errors, with RMSE = 9.96 and ${R}^{2}$ = 0.37.

Integrating feature augmentation and risk-aware learning produces the largest improvement for MLR. FA-RA MLR reduces the testing RMSE from 9.96 to 3.22 and increases ${R}^{2}$ from 0.37 to 0.96, corresponding to a 67% reduction in RMSE. This result shows that polynomial–harmonic augmented mechanical features enable a transparent linear model to capture nonlinear multiaxial response patterns that are poorly represented by raw loads alone. For nonlinear models such as XGBoost and BP-NN, which already show strong baseline accuracy, FA/RA mainly improves residual balance, prediction stability, and sensitivity to upper-tail response states; for example, FA-RA BP-NN achieves the smallest residual mean offset of 0.13 [Supplementary Table S1].

Prediction–observation and residual analyses further support these trends. Baseline MLR underestimates critical stress in several high-stress testing samples, whereas FA-RA MLR aligns more closely with the perfect-fit line and keeps most testing predictions within the ±5% relative error band. FA/RA-enhanced RF, XGBoost, and BP-NN models show more moderate but consistent improvements in prediction alignment, residual symmetry, and systematic offset. These observations indicate that the proposed strategies are particularly useful for stabilizing predictions in upper-tail stress regions, where accurate response estimation is important for subsequent risk assessment. This finding is consistent with previous studies showing that embedding domain or physics information into learning pipelines and addressing response imbalance can improve prediction robustness in engineering applications^25,26^. Detailed critical-stress residual diagnostics are provided in Supplementary Note 1 and Supplementary Fig. S1.

The bolt pretension-degradation task shows a consistent pattern. Under the joint risk-aware objective, FA-RA MLR achieves an overall ${R}^{2}=0.90$, with most testing samples falling within the ±15% relative error band. The predictions remain stable even for bolts with sparse high-loss samples, indicating that the proposed learning strategy improves prediction consistency for both critical stress and connection-level degradation. Detailed bolt-level prediction results are provided in Supplementary Note 1 and Supplementary Fig. S2. Together, the predicted stress and pretension-degradation responses provide the response basis for the DRS-based risk assessment in the next section.

### Structural risk assessment using the unified DRS

The predicted critical stress and bolt pretension degradation from the previous section were integrated into the Degradation Risk Score (DRS) framework to quantify sample-wise structural vulnerability. For each loading case i, the predicted critical stress ${\sigma }_{{crit},i}$ was converted into the stress-margin degradation component ${D}_{{{{\rm{res}}}},{i}}$, while the four bolt-level pretension losses were summarized by the maximum pretension degradation ${D}_{{{{\rm{pre}}}},{i}}$. The overall DRS was then computed through multiplicative coupling of stress-margin degradation and connection-level degradation, as defined in the section “*Methods*”.

Across the 120 load samples, most cases showed low DRS values, indicating relatively low stress-margin degradation and limited bolt pretension loss. A smaller subset shifted toward higher DRS values when ${D}_{{res},i}$, ${D}_{{pre},{i}}$, or both increased. This monotonic increase indicates that the DRS responds to two distinct vulnerability sources and highlights compound degradation states that would be difficult to prioritize using stress or pretension loss alone. The full sample-wise DRS distribution is shown in Supplementary Fig. S3a.

To connect degradation scoring with reliability assessment, the sample-wise structural reliability can be expressed as:

1 $${R}_{i}=1-{P}_{f,i}$$

where ${P}_{f,{i}}$ denotes the component-level failure probability for loading case i obtained after Bayesian calibration when inspection, testing, or monitoring observations are available. For multiple loading scenarios or temporal monitoring samples, the system-level reliability can be estimated as:

2 $${R}_{{system}}={\prod}_{i}(1-{P}_{f,i})={\prod}_{i}{R}_{i}$$

This provides a direct route from multiaxial response prediction to reliability-oriented decision-making. The corresponding reliability transition is shown in Supplementary Fig. S3b.

Bayesian logistic and Weibull calibration models provide two complementary routes for mapping ${D}_{{{{\rm{res}}}}}$, ${D}_{{{{\rm{pre}}}}}$, or DRS to ${P}_{f}$ when inspection, testing, or monitoring observations are available. The logistic form explicitly represents the separate and coupled effects of stress-margin degradation and pretension degradation, whereas the Weibull form uses DRS as a scalar vulnerability input. Detailed Bayesian updating procedures are provided in Supplementary Note 2. Together, this DRS–Bayesian framework enables sample-wise risk ranking, system-level reliability estimation, and component-level probabilistic calibration to support inspection prioritization and preventive maintenance.

## Discussion

The results show that mechanics-informed feature augmentation and risk-aware learning improve reliability-oriented prediction by addressing two coupled challenges in multiaxial structural systems: nonlinear load–response interactions and imbalanced degradation responses. The strong improvement of FA-RA MLR indicates that the augmented feature space does not simply increase input dimensionality, but provides a physics-aligned representation that allows even a transparent linear model to capture nonlinear multiaxial response patterns. This is supported by SHAP analysis^27,28^, where dominant predictors are mainly polynomial interaction features involving load-transfer points LP2 and LP4 rather than isolated raw load components. Detailed global and sample-wise SHAP interpretations are provided in Supplementary Discussion 1 and Supplementary Figs. S4 and 5.

The risk-aware component provides a complementary effect. Structural degradation data are strongly imbalanced, with most samples located in low- or moderate-response regimes and only a small subset occupying upper-tail stress or pretension-loss regions. The Weibull-based weighting strategy increases the training contribution of these upper-tail samples without imposing hard binary failure thresholds. This explains why FA/RA improves not only average prediction accuracy but also residual stability in high-response regions. Detailed residual diagnostics and bolt-level prediction analyses are provided in Supplementary Note 1 and Supplementary Figs. S1 and 2.

The Degradation Risk Score (DRS) further converts predicted stress-margin degradation and bolt pretension degradation into a normalized vulnerability indicator. This score is useful for ranking loading cases according to compounded degradation severity, especially when stress demand and connection degradation occur simultaneously. Importantly, DRS should not be interpreted as a directly calibrated probability of failure. Instead, Bayesian logistic and Weibull calibration provide a probability-calibration layer that can map ${D}_{{res},i}$, ${D}_{{pre},{i}}$ or DRS to component-level failure probability when inspection, testing, or monitoring data become available. Detailed Bayesian updating procedures are provided in Supplementary Note 2.

From a model-selection perspective, the results suggest that transparent models with physics-aligned feature spaces can perform competitively in moderate-sample regimes. In the present dataset, FA-RA MLR achieves the best overall testing performance, whereas more flexible models such as XGBoost and BP-NN mainly benefit from improved residual balance and upper-tail sensitivity. This does not imply that linear models are universally preferable. Instead, it shows that when physically meaningful nonlinear interactions are explicitly encoded as augmented features, even a transparent linear model can capture key multiaxial response patterns without relying solely on highly flexible model architectures.

The framework can be adapted to other multiaxial jointed systems by redefining the load descriptors, augmented feature basis, and degradation indicators according to the mechanics of the target structure. In uncertain real-world environments, the workflow can operate as a real-time monitoring loop: online structural load data are collected from sensors and passed into the calibrated FA/RA framework; predicted stress and connection degradation are integrated into DRS; DRS or its components are then mapped to failure probability through Bayesian calibration; and high-risk alerts can be issued to guide inspection or preventive intervention. This provides a pathway toward reliability-centered maintenance and proactive prevention of engineering accidents. However, the present validation is based on MBD- and FEA-derived data from a bolted steering-knuckle assembly. Future work should incorporate component-level experiments, field monitoring data, and online uncertainty quantification to further evaluate generalization across different structures, materials, and operating environments.

## Methods

The proposed framework was designed to predict structural reliability under complex multiaxial loading by combining simulation-based response generation, mechanics-informed feature augmentation, risk-aware learning, degradation risk scoring, and Bayesian probability calibration. A bolted steering-knuckle assembly was used as the demonstration system, as it contains two coupled degradation pathways: material overstress in the knuckle and connection-level pretension loss in the bolts.

The Methods are organized according to this workflow. First, six-degree-of-freedom service loads were reconstructed using MBD and applied to a finite element model to obtain critical stress and bolt pretension degradation responses. Second, the original load cases were statistically expanded to improve coverage of the admissible multiaxial load space, with each additional case evaluated through FEA rather than label interpolation. Third, the raw force–moment inputs were transformed into a polynomial–harmonic augmented feature space, and a Weibull-based weighting function was introduced to emphasize upper-tail stress and pretension-degradation responses during model training.

The resulting learning configurations were evaluated using predictive performance and residual-based metrics. Predicted stress and pretension degradation were then integrated into a unified Degradation Risk Score (DRS), which provides a normalized vulnerability indicator for each loading case. When inspection, testing, or monitoring observations are available, the DRS or its components can be mapped to calibrated component-level failure probability through Bayesian logistic or Weibull calibration models.

### Multiaxial load extraction and finite element simulation

Service-induced multiaxial loading of the bolted steering-knuckle assembly was reconstructed using MBD^29^. Vehicle event scenarios were simulated to obtain six-degree-of-freedom force–moment histories (${F}_{x},{F}_{y},{F}_{z},{M}_{x},{M}_{y},{M}_{z}$) at four load-transfer points, denoted LP1–LP4, along the knuckle–control arm interface. The load-acquisition procedure and its mapping to the finite-element load points are shown in Supplementary Fig. S6. From these time histories, twenty representative load cases were selected according to peak combined force–moment magnitudes to cover high-stress excitation states. This load-case extraction strategy can be adapted to analogous jointed components by redefining load-transfer points, input variables, and criticality criteria according to the target structure^30^. Finite-element simulations were then used to extract the structural response of the assembly under each selected multiaxial load case. The simulation platform, constitutive material models, and other details are described in Supplementary Method 1.

**Fig. 3:**
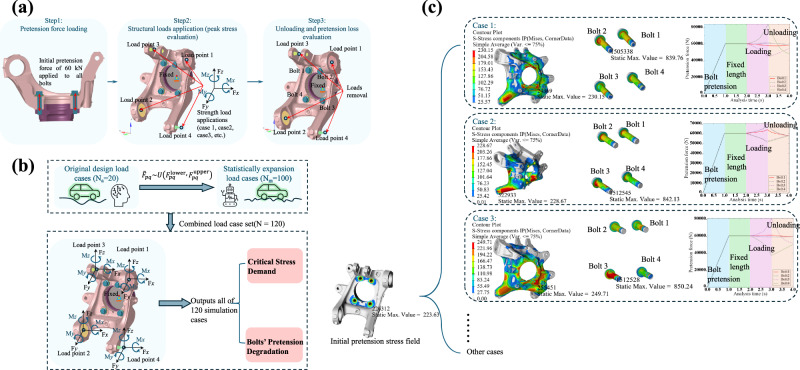
Finite-element simulation protocol, load-space expansion and representative responses. **a** Sequential finite-element protocol for the bolted steering-knuckle assembly, including bolt preloading, multiaxial service loading, unloading, and extraction of residual bolt forces. **b** Statistical expansion of multiaxial load cases from twenty baseline cases to 120 FEA samples using randomized sampling within physically admissible load bounds. **c** Representative FEA responses for selected load cases, showing critical stress distribution in the knuckle and bolt pretension degradation.

A sequential finite-element protocol was used to quantify both critical stress and bolt pretension degradation, as shown in Fig. 3a. First, an initial bolt pretension of 60 kN was applied to reproduce the assembly preload and establish contact. Second, multiaxial service loads were imposed at LP1–LP4 through load-transfer constraints. Third, the external loads were removed, and residual bolt forces were extracted to quantify load-induced pretension loss. For bolt j, the pretension degradation was calculated as:

3 $${D}_{{pre},j}=\frac{{F}_{{pre},j}^{{initial}}-{F}_{{pre},j}^{{residual}}}{{F}_{{pre},j}^{{initial}}}\times 100 \%$$

where ${F}_{{pre},j}^{{initial}}$ and ${F}_{{pre},j}^{{residual}}$ denote the initial and residual pretension forces of bolt j, respectively.

For each loading case, the finite-element outputs included the peak von Mises stress of the knuckle and the pretension degradation of the four bolts. Representative FEA responses are shown in Fig. 3c. Across the twenty baseline cases, peak knuckle stress ranged from 223.09 MPa to 248.42 MPa, while bolt pretension degradation varied substantially between cases and bolts. For example, bolt 4 in Case 2 exhibited more than 20% pretension loss, whereas the corresponding loss was only 0.13% in Case 18. The complete baseline response values for the twenty load cases are provided in Supplementary Table S3. These results indicate strong coupling between global multiaxial load paths and localized contact-driven degradation.

### Statistical expansion of load scenarios

The twenty multibody-derived load cases capture representative service events but cannot fully cover the broader uncertainty encountered during real operation, such as impact loading, road irregularities, local bumps and potholes, wind-induced disturbances, and other stochastic operating conditions^31^. Training a predictive model only on these limited simulated events may cause the model to learn event-specific response patterns and reduce its applicability to unseen operating conditions. To improve robustness while retaining physical plausibility, we expanded the load space by randomized sampling within an adverse load envelope derived from the multibody simulations. This follows reliability-oriented sampling strategies for improving design-space coverage under uncertainty^32^.

For each loading point $p\in \left\{1,\,2,\,3,\,4\right\}$ and load component $q\in \{{F}_{x},{F}_{y},{F}_{z},{M}_{x},{M}_{y},{M}_{z}\}$, the minimum and maximum values across the original twenty cases were recorded as ${F}_{{pq}}^{\min }$ and ${F}_{{pq}}^{\max }$. To allow limited boundary expansion while avoiding unrealistic load amplitudes, the bounds were expanded using a factor ${\alpha }^{l}=1.1$:

4 $$\left\{\begin{array}{c}{{F}_{{pq}}^{{{{\rm{lower}}}}}={\alpha }^{l}\cdot F}_{{pq}}^{\min }\\ {{F}_{{pq}}^{{{{\rm{upper}}}}}={\alpha }^{l}\cdot F}_{{pq}}^{\max }\end{array}\right.$$

Each synthetic component ${\widetilde{F}}_{{pq}}$ was then sampled from a uniform distribution:

5 $${\widetilde{F}}_{{pq}} \sim U({F}_{{pq}}^{{{{\rm{lower}}}}},{F}_{{pq}}^{{{{\rm{upper}}}}})$$

Repeating this process across all load points and load components yielded 100 new 24-dimensional multiaxial load vectors:

6 $${\widetilde{F}}_{{pq}}^{(k)}\in {{\mathbb{R}}}^{24},k=1,\,2,\,3,\ldots \ldots ,\,100$$

The statistical expansion procedure is illustrated in Fig. 3b. Importantly, the additional cases were not generated by interpolating response labels; each sampled load vector was evaluated using the same FE protocol described in Fig. 3a to obtain its corresponding stress response and bolt pretension degradation. Together with the original twenty load cases, this produced 120 FEA samples. Each sample contained a 24-dimensional multiaxial load input vector, the corresponding peak von Mises stress of the knuckle, and pretension degradation values for the four bolts. Further rationale and dataset composition are provided in Supplementary Method 2 and the “*Data availability*” section.

### Mechanics-informed feature augmentation

**Fig. 4:**
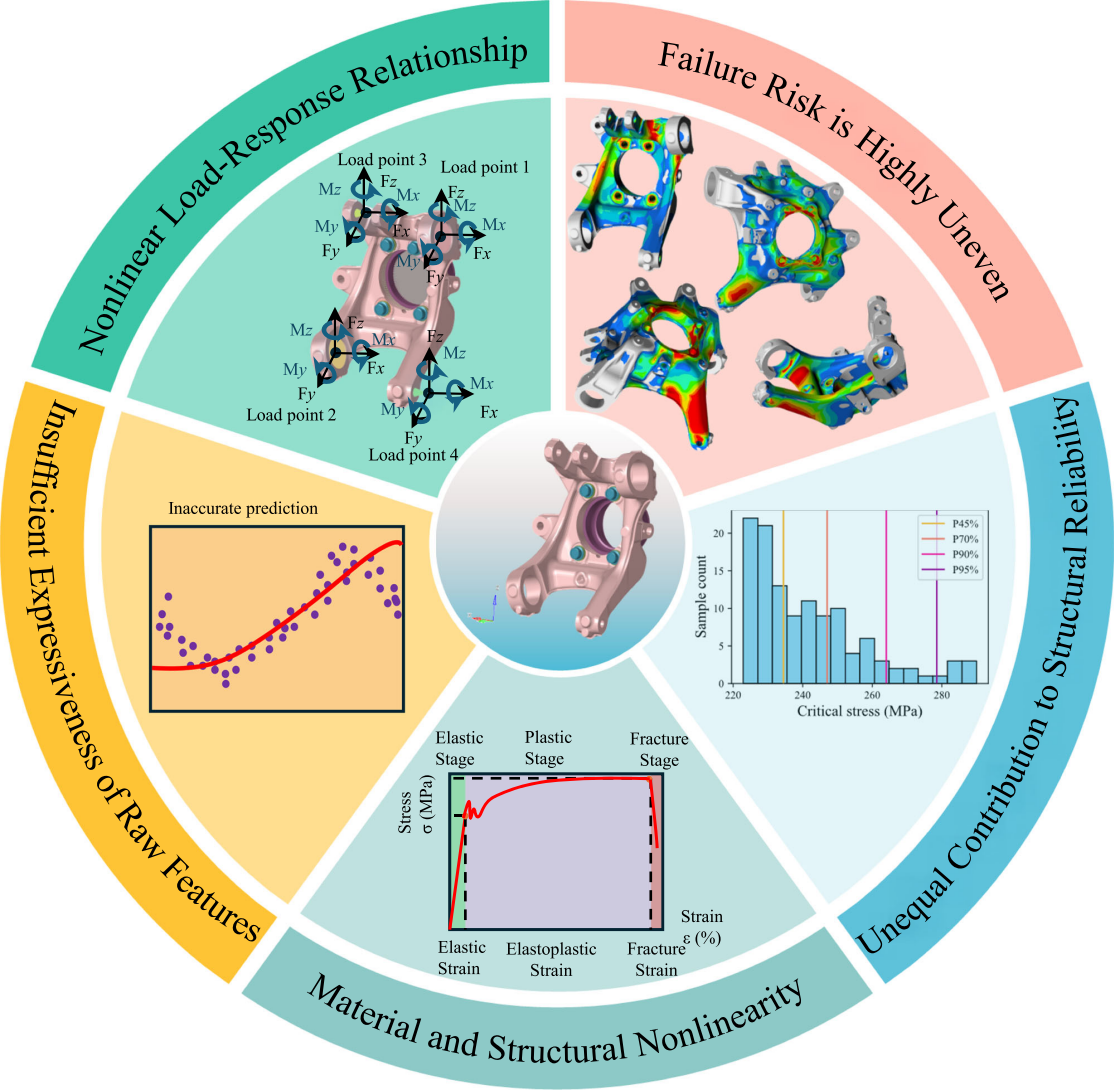
Motivation for mechanics-informed feature augmentation and risk-aware learning. The proposed framework addresses five challenges in multiaxial reliability prediction: nonlinear load–response relationships, material and structural nonlinearity, insufficient expressiveness of raw load features, highly uneven failure risk, and unequal sample contributions to structural reliability. These factors motivate the integration of polynomial–harmonic feature augmentation and risk-aware learning.

Machine-learning prediction under complex multiaxial loading is challenging because structural responses are governed by nonlinear load–response relationships, material and structural nonlinearity, localized stress redistribution, and uneven degradation risk. As summarized in Fig. 4, raw force–moment inputs alone may not sufficiently represent the coupled mechanics of multiaxial jointed structures, particularly when rare upper-tail stress or pretension-loss responses dominate reliability assessment^1,33–36^. This motivated the use of mechanics-informed feature augmentation to improve input representation before model training.

For each loading case, the raw input was a 24-dimensional force–moment vector, consisting of six load components $\left({F}_{x},|,{F}_{y},|,{F}_{z},|,{M}_{x},|,{M}_{y},|,{M}_{z}\right)$ at four load-transfer points. To embed nonlinear load interactions explicitly, the raw load matrix $X\in {{\mathbb{R}}}^{n\times d}$, where n is the number of samples and d=24, was transformed into an augmented feature matrix:

7 $${{{\mathcal{N}}}}\left(X\right)=\left[X\left|{\Phi }^{{{{\rm{poly}}}}}\left(X\right)\right.\left|{\Phi }^{\sin }\left(X\right)\right.\left|{\Phi }^{\cos }\left(X\right)\right.\right]\in {{\mathbb{R}}}^{n\times {d}^{{\prime} }}$$

where ${\Phi }^{{{{\rm{poly}}}}}$, ${\Phi }^{\sin }$ and ${\Phi }^{\cos }$ denote polynomial interaction features, sine features, and cosine features, respectively.

The polynomial terms encode squared and pairwise force–moment interactions, allowing the model to represent nonlinear coupling among load components. For a sample vector $X=({x}_{1},{x}_{2},{x}_{3},\ldots ,{x}_{d})$, the second-order polynomial terms were defined as

8 $${\Phi }^{{{{\rm{poly}}}}}\left(x\right)=\left\{\left.{\prod }_{i=1}^{d}{x}_{i}^{{\alpha }_{i}}\right|{\sum}_{i=1}^{d}{\alpha }_{i}\le p,{\alpha }_{i}\in {{\mathbb{N}}}_{0}\right\}$$

where the total degree p=2.

Harmonic terms were further introduced to capture sign-dependent or oscillatory response patterns that may arise under cyclic or vibration-sensitive loading conditions^37,38^:

9 $${\Phi }^{\sin }\left(x\right)={\left[\sin (\frac{m}{K}\cdot x)\right]}_{m=1}^{s},\,{\Phi }^{\cos }\left(x\right)={\left[\cos (\frac{m}{K}\cdot x)\right]}_{m=1}^{c}$$

where K is a scaling factor used to control the harmonic feature range. In this study, the original 24-dimensional load vector was expanded into a 372-dimensional augmented feature vector, consisting of 24 raw features, 300 second-order polynomial features, 24 sine features, and 24 cosine features. The raw-feature correlation analysis and full feature-space expansion are provided in Supplementary Method 3 and Supplementary Fig. S9.

### Weibull-based risk-aware learning

Although the statistical expansion in section “*Statistical expansion of load scenarios*” broadens the sampled multiaxial load space using randomized uniform sampling, the resulting structural responses are not uniformly distributed in terms of risk. After nonlinear load interaction, contact redistribution, and bolt-level degradation mechanisms, most FE-simulated samples remain in low- or moderate-response regimes, whereas only a small subset exhibits upper-tail critical stress or severe pretension loss. This creates a mismatch between load-space coverage and risk-space representation: engineering decisions are often governed by rare high-risk states, but standard training losses can be dominated by abundant low-risk samples. The response imbalance is quantified in Supplementary Method 4.

To address this imbalance, response-dependent risk-aware learning was introduced. The response distributions used to motivate this strategy are shown in Supplementary Fig. S10. In the present dataset, high-stress or severe-degradation samples occupy only a small portion of the response space, which can reduce model sensitivity in safety-relevant regimes if all samples are treated equally^39^.

A Weibull-type cumulative function was used to construct sample weights that increase smoothly with response severity. The Weibull function was not used here as a calibrated failure-probability model; instead, it served as a monotonic weighting curve for emphasizing structurally critical responses during training. For a response variable z, the Weibull cumulative function is

10 $$F\left({z;}\lambda ,k\right)=1-{{{{\rm{e}}}}}^{-{(\frac{z}{\lambda })}^{k}}$$

where λ>0 is the scale parameter and k>0 is the shape parameter. Owing to its flexibility in representing increasing failure tendency, the Weibull distribution is widely used in reliability engineering^40,41^. In this framework, $F({z;}\lambda ,k)$ was interpreted as a response-severity accumulation function rather than a direct probability of failure.

The sample-wise risk weight was then defined as

11 $${\omega }_{{risk}}\left(z\right)=1+{\alpha }_{r}\cdot F\left({z;}\lambda ,k\right)$$

where ${\alpha }_{r} > 0$ controls the additional emphasis assigned to upper-tail response samples. This formulation preserves near-unit weights for low-severity samples while increasing the contribution of stress-critical or degradation-sensitive samples. The resulting weight distributions for critical stress and bolt pretension degradation are shown in Supplementary Fig. S11.

For critical-stress prediction, the weighted training objective was

12 $${{{{\mathcal{L}}}}}_{{stress}}=\frac{1}{N}{\sum}_{i=1}^{N}{\omega }_{{risk}}\left({y}_{i}\right){\cdot }{\ell}({y}_{i},{\hat{y}}_{i})$$

where ${y}_{i}$ and ${\hat{y}}_{i}$ denote the FE-simulated and predicted stress responses, respectively, ${\ell}\left(\cdot \right)$ is the base loss function, and N is the number of training samples. For bolt pretension degradation, the objective was extended to four bolt-level outputs:

13 $${{{{\mathcal{L}}}}}_{{pretension}}={\sum}_{j=1}^{4}\frac{1}{N}{\sum}_{i=1}^{N}{\omega }_{{risk}}^{(j)}\left({y}_{i}\right)\cdot {{{\mathcal{l}}}}({y}_{i}^{(j)},{\hat{y}}_{i}^{(j)})$$

where ${y}_{i}^{\left(j\right)}$ and ${\hat{y}}_{i}^{\left(j\right)}$ are the FE-simulated and predicted pretension degradation of bolt j, respectively. This risk-aware objective guides the model toward upper-tail stress and pretension-loss regimes without imposing hard binary failure thresholds, improving robustness under imbalanced degradation responses^42,43^.

### Predictive model configurations and evaluation metrics

**Fig. 5:**
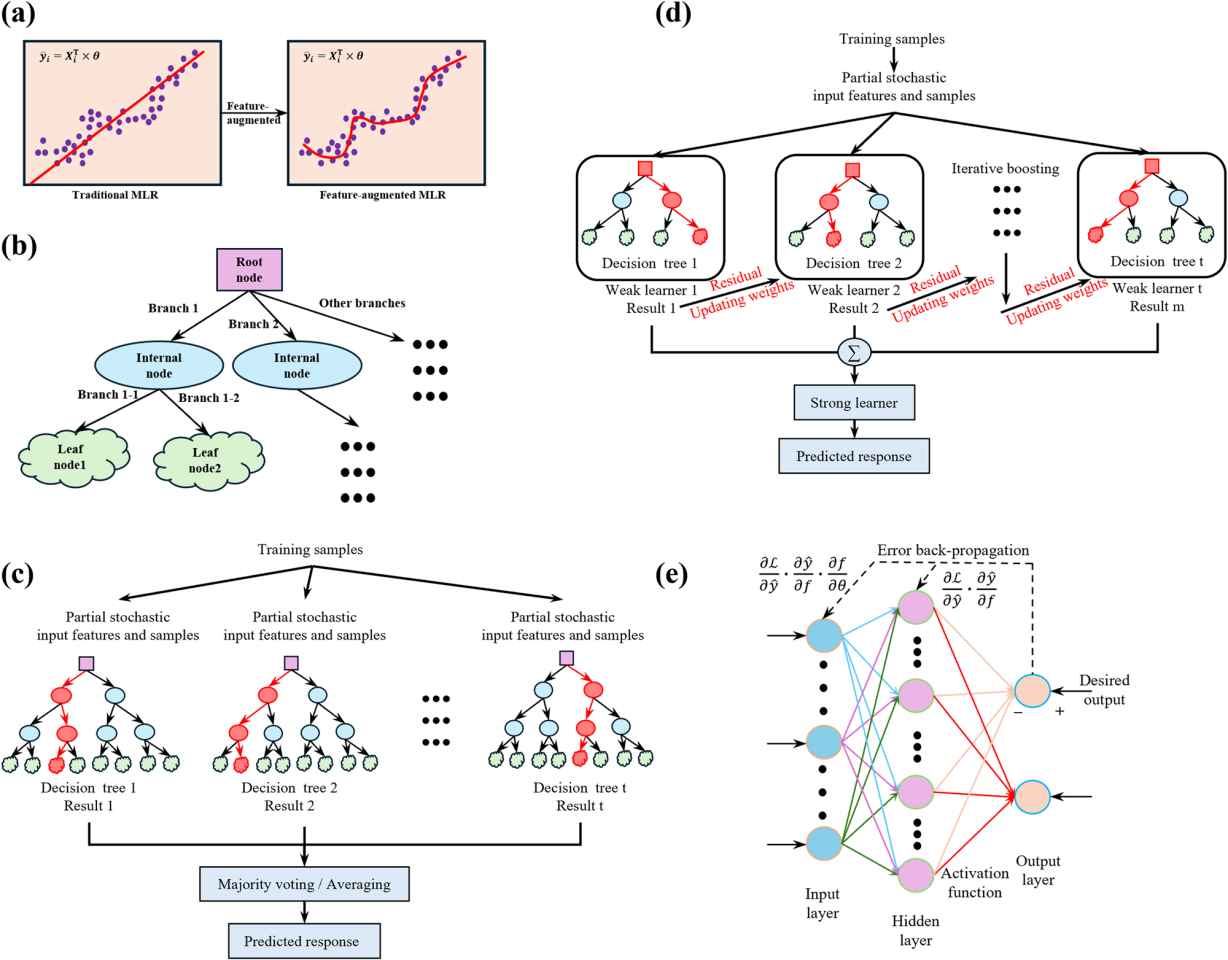
Predictive model architectures used for comparative benchmarking. **a** Traditional and feature-augmented MLR; **b** single decision tree; **c** random forest; **d** XGBoost; and **e** BP-NN. These models span interpretable linear regression, ensemble tree learning, gradient boosting, and neural-network-based nonlinear mapping.

To evaluate the effects of mechanics-informed feature augmentation and risk-aware learning across different model families, four representative regression models were selected: multiple linear regression (MLR), random forest (RF), extreme gradient boosting (XGBoost), and backpropagation neural network (BP-NN). These models cover interpretable linear regression, ensemble tree learning, gradient boosting, and neural-network-based nonlinear mapping, enabling controlled comparison for two prediction tasks: critical-stress estimation and bolt pretension degradation. Their schematic structures are shown in Fig. 5, and the mathematical formulations are summarized in Supplementary Method 5.

Three learning configurations were constructed to isolate the effects of feature augmentation and risk-aware weighting. The baseline configuration used raw load features and uniform sample weights. The full FA/RA configuration combined polynomial–harmonic feature augmentation with Weibull-based response-dependent weighting. An additional RA-only BP-NN configuration was included as an ablation case to evaluate whether risk-aware weighting alone could improve neural-network performance without augmented features. The full configuration matrix is provided in Supplementary Table S4. In total, nine learning configurations were evaluated for both critical-stress and bolt pretension-degradation prediction. The training–testing datasets across prediction targets are described in Supplementary Method 5, confirming that both subsets cover the main response range and include upper-tail response samples.

Predictive performance was assessed using seven metrics: root mean squared error (RMSE), mean squared error (MSE), mean absolute error (MAE), mean absolute percentage error (MAPE), relative absolute error (RAE), coefficient of determination $\left({{{{\rm{R}}}}}^{2}\right)$, and residual mean offset (RMO). These metrics jointly quantify absolute error, relative error, goodness of fit, and residual bias, and are commonly used for benchmarking predictive models in structural engineering applications^44^. Their mathematical definitions are provided in Supplementary Table S5.

### Degradation risk score and Bayesian calibration

To convert predicted structural responses into a unified vulnerability indicator, critical stress and bolt pretension degradation were integrated into a Degradation Risk Score (DRS). The DRS combines two degradation pathways: stress-margin degradation in the knuckle and connection-level degradation caused by bolt pretension loss.

For loading case i, the predicted critical stress ${\sigma }_{{{{\rm{crit}}}},i}$ was first converted into a normalized stress-margin degradation component:

14 $${D}_{{{{\rm{res}}}},i}=\left\{\begin{array}{cc}0, & {\sigma }_{{crit},i} < {\sigma }_{y}\\ \frac{{\sigma }_{{crit},i}-{\sigma }_{y}}{{\sigma }_{u}-{\sigma }_{y}}, & {\sigma }_{y}\le {\sigma }_{{crit},i}\le {\sigma }_{u}\\ 1, & {\sigma }_{{crit},i}\ge {\sigma }_{u}\end{array}\right.$$

where ${\sigma }_{y}$ and ${\sigma }_{u}$ are the yield and ultimate strengths of the knuckle material, respectively. This formulation assigns zero degradation when the predicted stress remains below yield strength and increases the degradation value toward one as the stress approaches or exceeds the ultimate strength.

The connection-level degradation component was defined using the maximum predicted pretension loss among the four bolts. For sample i:

15 $${D}_{{{{\rm{pre}}}},i}={{{\rm{clip}}}}[{\max }_{j=1,2,3,4}{\left({D}_{{{{\rm{pre}}}},j}\right)}_{i},0,\,1]$$

where ${({D}_{{{{\rm{pre}}}},j})}_{i}$ is the fractional pretension degradation of bolt j in sample i, and ${{{\rm{clip}}}}[\cdot ,{{\mathrm{0,1}}}]$ restricts the value to the interval [0,1]. This maximum-loss definition reflects the fact that severe degradation in a single bolt can dominate connection-level vulnerability.

The overall DRS was then computed through multiplicative coupling:

16 $${{DRS}}_{i}=1-\left(1-{D}_{{res},i}\right)\left(1-{D}_{{pre},i}\right)$$

This formulation ensures that $0\le {DR}{S}_{i}\le 1$, where values close to 0 indicate low predicted degradation and values close to 1 indicate severe combined stress-margin and connection-level degradation. The multiplicative form allows either material overstress or bolt pretension loss to increase the score, while simultaneous degradation in both pathways leads to a stronger increase in vulnerability.

The DRS was used as a normalized vulnerability score rather than as a directly calibrated probability of failure. When inspection, testing, or monitoring observations are available, ${D}_{{res}}$, ${D}_{{pre}}$, or DRS can be mapped to component-level failure probability through Bayesian calibration. The Bayesian logistic and Weibull calibration formulations used to map degradation indicators to failure probability are provided in Supplementary Note 2.

### Reporting summary

Further information on research design is available in the Nature Portfolio Reporting Summary linked to this article.

## Supplementary information

Transparent Peer Review file

Supplementary information for Manuscript

Description of Additional Supplementary Files

Supplementary Data 1

Supplementary Data 2

Reporting Summary

## Data Availability

The datasets generated and analyzed during the current study are publicly available in the GitHub repository at: https://github.com/Peaker99/Simulations-of-Steering-knuckle-System.git. The repository contains the finite-element analysis models and simulation datasets used in this study, including the 24-dimensional multiaxial load inputs, peak von Mises stress responses, and bolt pretension degradation results used for model training, performance evaluation, and degradation risk assessment.

## Code availability

The custom machine-learning and deep-learning codes developed and used in this study are publicly available in the GitHub repository: https://github.com/Peaker99/Risk-ware-learning-with-mechanics-informed-feature-augmentation.git. The repository contains the implementation scripts for all predictive models and ablation studies reported in this work. The scripts include the corresponding model configurations, hyperparameter settings, explanatory comments, and commands required to reproduce the reported analyses and figures. The code is available without restrictions.
