# Supplementary material for: Mechanics-informed risk-aware learning for multiaxial structural reliability with Bayesian calibration
Source: Commun Eng. 2026 Aug 14;5:148. doi: 10.1038/s44172-026-00752-y (PMC13476256; doi:10.1038/s44172-026-00752-y)
Supplement: Supplementary file 1 [file 44172_2026_752_MOESM1_ESM.pdf]

nature portfolio

Peer Review File

Mechanics-Informed Risk-Aware Learning for Multiaxial Structural Reliability with Bayesian Calibration

Corresponding Author: Mr Gaofeng Zhang

This file contains all reviewer reports in order by version, followed by all author rebuttals in order by version.

Attachments originally included by the reviewers as part of their assessment can be found at the end of this file.

Version 0:

Reviewer comments:

Reviewer #1

(Remarks to the Author)
The manuscript introduces a mechanics-informed, risk-aware learning framework for predicting structural performance under complex multiaxial loading. It combines polynomial–harmonic feature augmentation, Weibull-based probabilistic sample reweighting, and a unified Degradation Risk Score (DRS) fusing stress margins and bolt pretension loss. Demonstrated on a bolted steering-knuckle assembly with FEA-derived data, the framework notably boosts baseline models (e.g., MLR from R^2=0.37 to 0.96) and claims improved sensitivity in high-risk regimes with interpretable SHAP attributions.

The dual integration of mechanics-informed features + risk-aware weighting with a scalar DRS is a thoughtful synthesis. Each ingredient exists in adjacent literature, but their combined, pragmatic pipeline for reliability-centric prediction under multiaxial loading is relatively novel.

Useful for risk-aware digital twins and reliability-centered maintenance of jointed structures; impact will hinge on validation beyond synthetic/FEA data.

Improvements on the presented dataset are strong, but generalization claims are overstated without external/component-level validation or real data.

The authors should include a discussion of related approaches based on degradation curve assumptions combined with Bayesian updating to adequately contextualize the contribution of the proposed framework.

The manuscript should provide a more detailed discussion of the training strategy, including the methods used for statistical expansion or augmentation of the training data, to clarify how model robustness and generalization are ensured.

The authors should discuss how the proposed framework could be adapted for real-time monitoring in environments with higher uncertainty.

Please consider reducing the number of figures and ensure the readability of all remaining visuals.

I recommend publication after the authors satisfactorily address the comments outlined above.

Reviewer #2

(Remarks to the Author)
My comments are attached.

Version 1:

Reviewer comments:

Reviewer #1

(Remarks to the Author)
All of my previous concerns have been satisfactorily addressed by the authors. The manuscript has been revised accordingly, and the changes have improved its clarity and quality.

I have no further comments or concerns. In my opinion, the manuscript is now suitable for publication in its present form.

Reviewer #2

(Remarks to the Author)
I am happy with the revision made by the authors. I recommend its publication.

Open Access This Peer Review File is licensed under a Creative Commons Attribution 4.0 International License, which permits use, sharing, adaptation, distribution and reproduction in any medium or format, as long as you give appropriate credit to the original author(s) and the source, provide a link to the Creative Commons license, and indicate if changes were made.
In cases where reviewers are anonymous, credit should be given to 'Anonymous Referee' and the source.
The images or other third party material in this Peer Review File are included in the article's Creative Commons license, unless indicated otherwise in a credit line to the material. If material is not included in the article's Creative Commons license and your intended use is not permitted by statutory regulation or exceeds the permitted use, you will need to obtain permission directly from the copyright holder.
To view a copy of this license, visit https://creativecommons.org/licenses/by/4.0/

Reviewer comments and changes made in accordance

Version 0:

Reviewer #1

The manuscript introduces a mechanics-informed, risk-aware learning framework for predicting structural performance under complex multiaxial loading. It combines polynomial–harmonic feature augmentation, Weibull-based probabilistic sample reweighting, and a unified Degradation Risk Score (DRS) fusing stress margins and bolt pretension loss. Demonstrated on a bolted steering-knuckle assembly with FEA-derived data, the framework notably boosts baseline models (e.g., MLR from R^2=0.37 to 0.96) and claims improved sensitivity in high-risk regimes with interpretable SHAP attributions.

The dual integration of mechanics-informed features + risk-aware weighting with a scalar DRS is a thoughtful synthesis. Each ingredient exists in adjacent literature, but their combined, pragmatic pipeline for reliability-centric prediction under multiaxial loading is relatively novel.

Useful for risk-aware digital twins and reliability-centered maintenance of jointed structures; impact will hinge on validation beyond synthetic/FEA data.

I recommend publication after the authors satisfactorily address the following comments:

Q1. Improvements on the presented dataset are strong, but generalization claims are overstated without external/component-level validation or real data.

Q2. The authors should include a discussion of related approaches based on degradation curve assumptions combined with Bayesian updating to adequately contextualize the contribution of the proposed framework.

Q3. The manuscript should provide a more detailed discussion of the training strategy, including the methods used for statistical expansion or augmentation of the training data, to clarify how model robustness and generalization are ensured.

Q4. The authors should discuss how the proposed framework could be adapted for real-time monitoring in environments with higher uncertainty.

Q5. Please consider reducing the number of figures and ensure the readability of all remaining visuals.

Response: We sincerely thank the reviewer for the careful evaluation of our manuscript and for the positive comments on the proposed mechanics-informed, risk-aware learning framework. We are grateful that the reviewer recognized the value of integrating mechanics-informed feature augmentation, Weibull-based risk-aware learning, and the Degradation Risk Score for reliability-oriented prediction under multiaxial loading. We also appreciate the constructive comments regarding the scope of generalization, Bayesian updating, training-data expansion, real-time monitoring, and figure readability. These comments were highly valuable for

improving the clarity, rigor, and presentation of the manuscript.

In response to the reviewer's suggestions, we have substantially revised the manuscript. In particular, we clarified the validation scope of the proposed framework, added a discussion of degradation-curve-based Bayesian updating approaches, expanded the description of the statistical load-space expansion strategy, discussed the potential adaptation of the framework for real-time monitoring under uncertainty, and reorganized the figures to improve readability. To comply with the journal formatting requirements and to improve the readability of the paper, the original manuscript has also been reorganized into two separate files: the revised main manuscript and the Supplementary Information. Detailed methodological derivations, diagnostic analyses, interpretability results, and supporting figures have been moved to Supplementary Information, while the main manuscript now focuses on the core workflow, principal results, and key methodological contributions.

In the following point-by-point response, the reviewer's comments are highlighted in **purple**, our responses are highlighted in **light blue**, and the most important revised or newly added content is highlighted in **red**. In the revised manuscript, key modifications have also been marked in **red** for ease of review. We again thank the reviewer for the thoughtful and constructive feedback, which has helped us significantly improve the manuscript.

Q1. Improvements on the presented dataset are strong, but generalization claims are overstated without external/component-level validation or real data.

Response: We agree that the generalization claims in the original manuscript were too strong, given that the present validation is based on multibody-dynamics- and finite-element-derived data from a single bolted steering-knuckle assembly rather than external component-level experiments or field monitoring data.

To address this concern, we revised the manuscript to distinguish more clearly between the performance demonstrated on the present FE-simulated dataset and the potential adaptability of the workflow to analogous multiaxial jointed systems**.** We removed or softened statements that implied direct cross-domain validation. In the revised manuscript, the framework is now described as adaptable only when the load descriptors, augmented feature basis, and degradation indicators are redefined according to the mechanics of the target structure.

We also added an explicit limitation statement in the Discussion. The revised manuscript now states that "the present validation is based on MBD- and FEA-derived data from a bolted steering-knuckle assembly" and that future work should incorporate "component-level experiments, field monitoring data, and online uncertainty quantification" to further evaluate generalization across different structures, materials, and operating environments. This revision makes the scope of the contribution more cautious and technically accurate.

.

Q2. The authors should include a discussion of related approaches based on degradation curve assumptions combined with Bayesian updating to adequately contextualize the contribution of the proposed framework.

Response: We thank the reviewer for this helpful suggestion. We agree that degradation-curve-

based reliability models combined with Bayesian updating provide an important context for the proposed framework. We have therefore revised the Introduction and the Bayesian calibration part of the manuscript to clarify how our work relates to these approaches.

In the revised Introduction, we now explain that physics-based methods, including finite-element analysis, fatigue-fracture mechanics, degradation-curve models and Bayesian updating, remain central to structural reliability assessment because they provide explicit mechanical interpretation of stress evolution, damage progression and failure thresholds. We further clarified that degradation-curve-based approaches combined with Bayesian updating are powerful when relevant degradation indicators and component-level observations are available, because the assumed relationship between degradation state and failure probability can be progressively updated using inspection, testing or monitoring data.

We also clarified the distinction between those approaches and the proposed framework. Conventional degradation-curve-based Bayesian methods usually begin with observed or assumed degradation trajectories and then update the degradation–failure relationship. In contrast, our framework first predicts mechanically meaningful degradation indicators from multiaxial force–moment inputs. Specifically, the FA/RA model predicts critical stress and bolt pretension degradation, these responses are converted into stress-margin degradation and pretension degradation, and the two pathways are integrated into the Degradation Risk Score (DRS). Bayesian logistic or Weibull calibration can then be applied as a probability-calibration layer when inspection, testing or monitoring observations are available.

This relationship is now emphasized in the revised framework figure. Fig. 1 illustrates the complete workflow from MBD/FEA load–response generation, mechanics-informed feature augmentation, risk-aware model training, DRS-based vulnerability assessment, and Bayesian failure-probability calibration. This figure helps position the proposed framework as a front-end mechanics-informed response prediction and risk-scoring module that can be combined with Bayesian updating, rather than as a replacement for degradation-curve-based reliability modelling.

In addition, Supplementary Note 2 now provides detailed Bayesian updating formulations. It includes a Bayesian logistic model using D_{res}, $D_{\mathrm{pre}}$and their interaction, and a Bayesian Weibull model using DRS as a scalar vulnerability input. These additions better contextualize the contribution of the proposed framework within existing Bayesian reliability modelling approaches.

Version before revised of Fig. 1

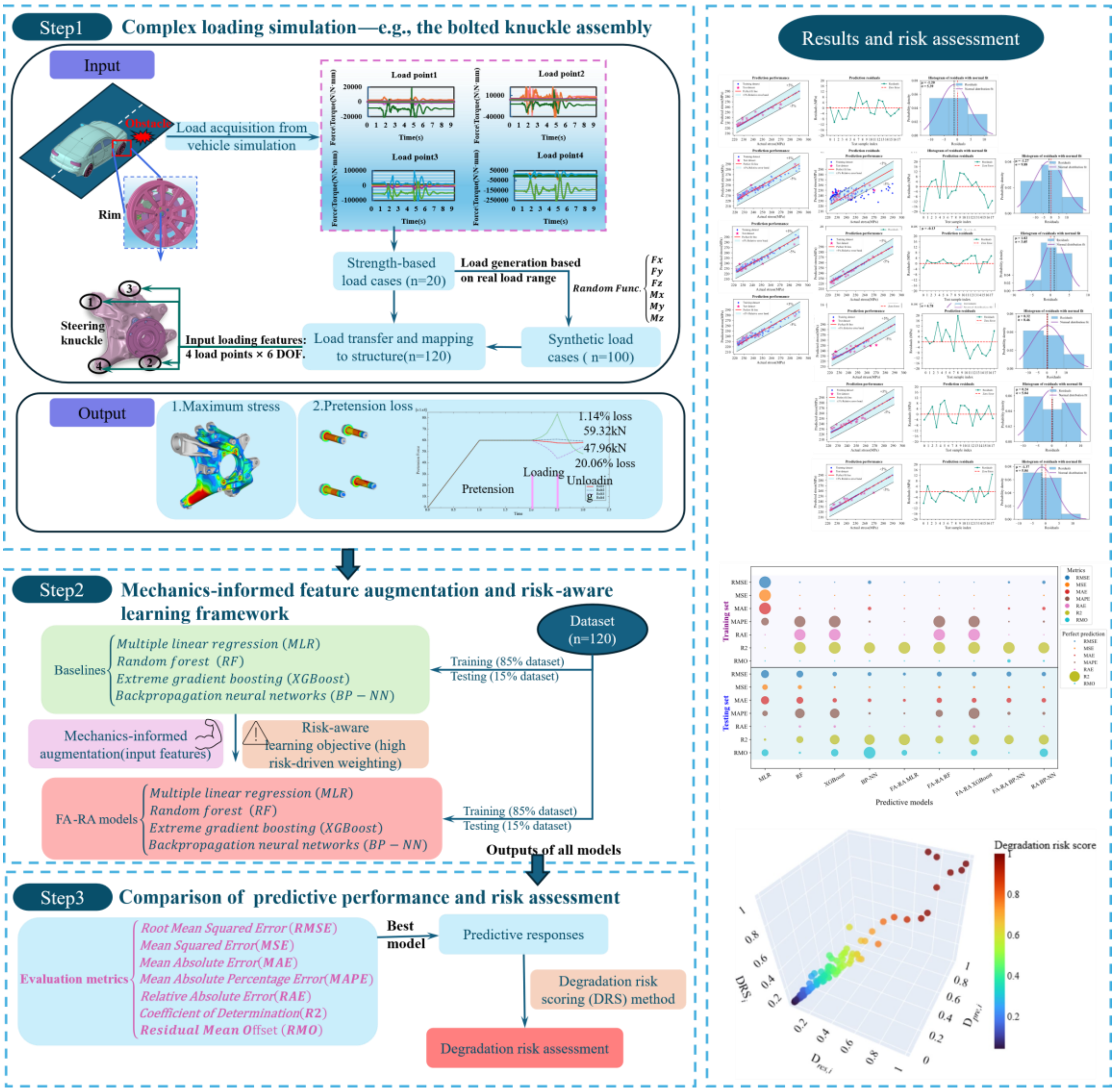

Fig. 1. **Proposed framework workflow:** Workflow of the proposed mechanics-informed, risk-aware learning framework for structural reliability prediction, integrating multiaxial load simulation, mechanics-guided feature augmentation, risk-aware training, and unified degradation risk scoring.

Revised version of Fig. 1

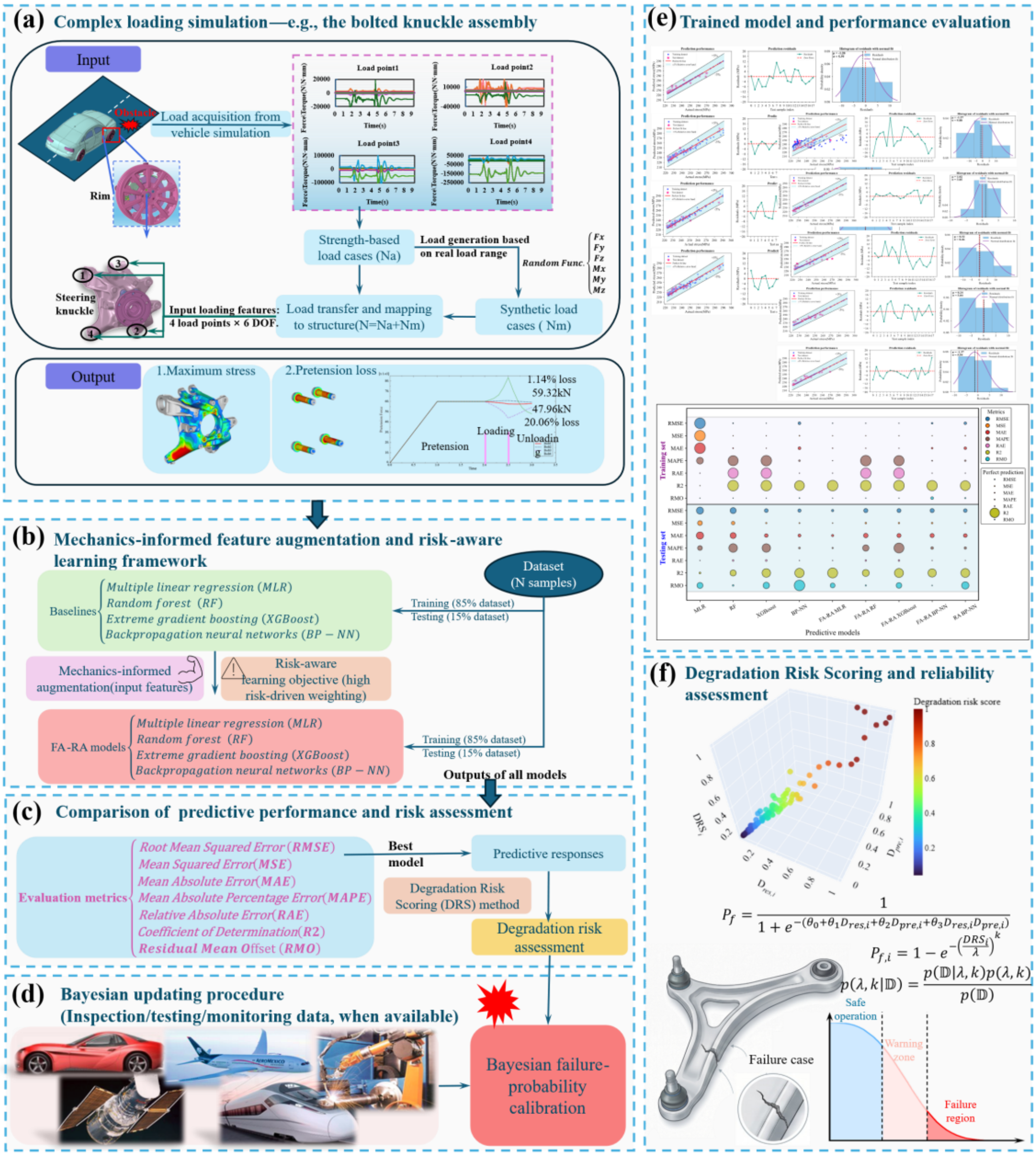

Fig. 1. Workflow of the proposed mechanics-informed, risk-aware learning framework for structural reliability prediction under multiaxial loading. (a) Multibody dynamics load acquisition and finite-element simulation, producing critical stress and bolt pretension degradation. (b) Mechanics-informed feature augmentation (FA) and risk-aware (RA) learning framework, showing baseline and FA-RA enhanced models. (c) Predictive performance comparison using seven residual-based evaluation metrics and subsequent DRS-based risk assessment. (d) Bayesian failure-probability calibration, incorporating inspection, testing, or monitoring data when available. (e) Trained model evaluation, showing predicted versus physical responses, residual distributions, and metric benchmarking across models. (f) Integration of DRS with Bayesian probability calibration to quantify sample-wise failure risk and structural reliability under monitoring or operational scenarios.

Q3. The manuscript should provide a more detailed discussion of the training strategy, including the methods used for statistical expansion or augmentation of the training data, to clarify how model robustness and generalization are ensured.

Response: We have substantially revised the Methods and Supplementary Information to provide a clearer explanation of the training-data expansion strategy and why it was used.

In the original manuscript, the statistical expansion procedure was described only briefly. In the revised manuscript, we now explain that the original twenty multibody-derived load cases represent a limited set of representative vehicle events and cannot cover the full uncertainty of real operation. Real service may involve additional stochastic load variations caused by impact loading, wind disturbance, road roughness, potholes, bumps and other uncertain operating conditions. Therefore, training only on the original MBD-derived cases could make the model overly dependent on event-specific response patterns.

To improve robustness while maintaining physical plausibility, the revised manuscript now describes a randomized uncertainty sampling strategy. The sampling was not performed in an arbitrary load domain. Instead, the MBD simulations were first used to identify an adverse but physically meaningful multiaxial load envelope. For each load-transfer point and each force–moment component, the lower and upper sampling bounds were obtained from the minimum and maximum values of the original twenty cases and then slightly expanded using $\alpha^l = 1.1$. Within this envelope, one hundred additional 24-dimensional load vectors were generated.

We also clarified that these additional samples were not produced by interpolating response labels. Each sampled load vector was evaluated using the same finite-element protocol as the original cases, so every additional sample has FE-simulated response targets, including peak von Mises stress and bolt pretension degradation. This ensures that the expanded dataset remains mechanically consistent while exposing the learning models to a broader range of force–moment combinations.

These revisions are now included in the Methods section "Statistical expansion of load scenarios" and in Supplementary Method 2. The main workflow of the expansion procedure is shown in **Fig. 3b**.

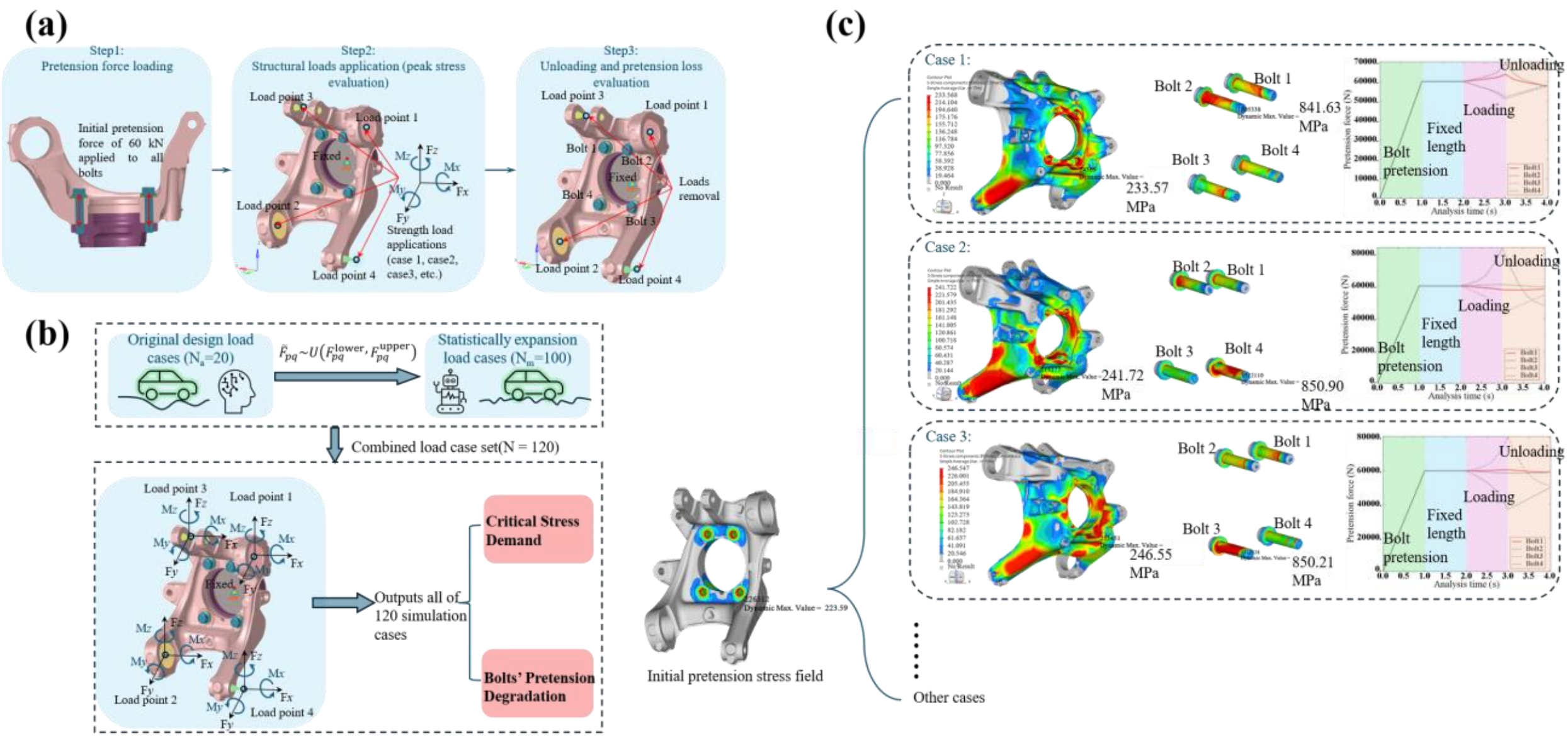

Fig. 3. Finite-element simulation protocol, load-space expansion and representative responses. (a) Sequential finite-element protocol for the bolted steering-knuckle assembly, including bolt preloading, multiaxial service loading, unloading, and extraction of residual bolt forces. (b) Statistical expansion of multiaxial load cases from twenty baseline cases to 120 FEA samples using randomized sampling within physically admissible load bounds. (c) Representative FEA responses for selected load cases, showing critical stress distribution in the knuckle and bolt pretension degradation.

Q4. The authors should discuss how the proposed framework could be adapted for real-time monitoring in environments with higher uncertainty.

Response: We have revised the Discussion to explain how the proposed framework could be adapted for real-time monitoring under high-uncertainty operating environments, while also clarifying that this is an adaptation pathway rather than a fully validated deployment in the present study.

In the revised Discussion, we describe the workflow as a sequential monitoring loop. Online structural load data can be collected from sensors and passed into the calibrated FA/RA framework. The model then predicts critical stress and connection-level degradation, which are integrated into DRS. When inspection, testing or monitoring observations are available, DRS or its components can be mapped to component-level failure probability through Bayesian calibration. High-risk alerts can then be issued to guide inspection or preventive intervention.

This revision clarifies how the proposed framework could operate under uncertainty: the FA/RA model provides real-time response prediction from incoming load descriptors, DRS provides sample-wise vulnerability ranking, and Bayesian calibration provides a route to update failure probability when observational data become available.

At the same time, we revised the Discussion to avoid overstating this capability. The manuscript now explicitly states that the present validation is based on MBD- and FEA-derived data, and that future real-time deployment requires sensor integration, component-level or field monitoring observations, and online uncertainty quantification.

Q5. Please consider reducing the number of figures and ensure the readability of all remaining visuals.

Response: We agree that the original manuscript contained too many figures in the main text, which reduced readability. We have therefore reorganized the figure layout and moved detailed diagnostic and supporting figures to Supplementary Information.

In the revised manuscript, the main text retains only the figures needed to understand the core workflow, key results and main methodology. Specifically, the main figures now focus on the overall framework, predictive performance benchmarking, finite-element protocol and load-space expansion, the motivation for mechanics-informed feature augmentation and risk-aware learning, and predictive model architectures.

Detailed supporting figures were moved to the Supplementary Information. These include residual diagnostics, bolt-level prediction plots, DRS distribution, SHAP beeswarm plots, SHAP heatmaps, response imbalance distributions, Weibull risk-weight distributions and training–testing distributions. This restructuring improves the readability of the main text while

preserving the detailed evidence needed for reproducibility and technical evaluation.

Reviewer #2

This paper presents a mechanics-informed, risk-aware machine learning framework for structural reliability prediction under multiaxial loading. The work connects physics-based simulation with data-driven prediction, based on feature augmentation, risk-aware weighting and unified risk scoring. Such development is important for reliability engineering. However, I have the following concerns about their work:

Q1. Explanations for considering the specific forms of nonlinearity are required to be connected to structural mechanics. Please explicitly map each feature family to a mechanics phenomenon. Explanation with intuition would be very much appreciated.

Q2. Check Eqn 2 for both positive and negative cases.

Q3. Could the degradation value be negative in some case? If yes, how to address this?

Q4. Authors should comment on possible overfitting issues.

Q5. Clearly mention the parameter values for reproducibility.

Q6. Authors may like to elaborate on the assumed form for DRS.

Response: We sincerely thank the reviewer for the careful evaluation of our manuscript and for recognizing the importance of connecting physics-based simulation with data-driven reliability prediction under multiaxial loading. We are grateful for the constructive comments regarding the mechanics interpretation of nonlinear feature augmentation, the treatment of signed load components, the bounded definition of degradation variables, possible overfitting, reproducibility, and the assumed form of the Degradation Risk Score. These comments were highly valuable for improving the technical clarity, methodological rigor, and reproducibility of the manuscript.

In response to the reviewer's suggestions, we have revised the manuscript and Supplementary Information in several aspects. First, we clarified the structural-mechanics motivation for the nonlinear feature families, including harmonic terms and pairwise interaction terms. Second, we clarified that the positive and negative values in the load-expansion equation represent signed Cartesian force–moment components rather than invalid negative load magnitudes. Third, we revised the degradation-score formulation to explicitly bound stress-margin degradation and bolt pretension degradation within physically meaningful intervals. Fourth, we added further discussion of overfitting control from the perspectives of load-case expansion, model regularization, cross-validation, dropout and training–testing performance comparison. Finally, we clarified the reproducibility information through the Data availability and Code availability statements and further explained the engineering interpretation of the DRS formulation.

To comply with the journal formatting requirements and to improve the readability of the paper, the original manuscript has also been reorganized into two separate files: the revised main manuscript and the Supplementary Information. The main manuscript now focuses on the core workflow, principal results and key methodological contributions, while detailed derivations, additional explanations, diagnostic analyses and supporting figures are provided in

the Supplementary Information. The key modifications in the revised main manuscript have been marked in **red** for ease of review. In the following point-by-point response, the reviewer's comments are highlighted in **purple**, our responses are highlighted in **light blue**, and the most important revised or explanatory content is highlighted in **red**.

Q1. Explanations for considering the specific forms of nonlinearity are required to be connected to structural mechanics. Please explicitly map each feature family to a mechanics phenomenon. Explanation with intuition would be very much appreciated.

Response: We thank the reviewer for this insightful comment. We agree that the original manuscript did not sufficiently explain why the specific nonlinear feature families were selected from a structural-mechanics perspective. In the revised response and manuscript clarification, we now explain that the feature augmentation is not intended as a purely algebraic expansion. Instead, it was designed as a mechanics-inspired feature basis to represent different nonlinear response mechanisms that commonly arise in multiaxial structural systems.

Specifically, the raw force–moment components represent the first-order effects of individual axial, shear, bending and torsional loads. The squared terms represent magnitude-dominated nonlinear effects, such as equivalent-stress amplification, local contact compression and yielding tendency, where the response may increase with load intensity even if the load direction changes. The pairwise interaction terms represent multiaxial load coupling, such as force–force, force–moment and moment–moment interactions. The harmonic sine and cosine terms were included to provide a bounded nonlinear basis inspired by vibration-driven structural response, load reversal and direction-sensitive dynamic effects.

For the harmonic terms, the motivation comes from classical structural dynamics and vibration theory **[Response References 1-4]**. For a damped single-degree-of-freedom structural system subjected to dynamic excitation, the governing equation can be written as:

$$m\ddot{u}(t) + c\dot{u}(t) + ku(t) = p(t)$$

where m, $c$and k denote mass, damping and stiffness, respectively, $u(t)$ is the structural response and $p(t)$ is the external excitation. When the excitation contains a harmonic component, for example:

$$p(t) = P_0\sin(\Omega t)$$

the analytical response consists of a transient component and a steady-state component:

$$u(t) = u_h(t) + u_p(t)$$

The homogeneous response is commonly expressed as:

$$u_h(t) = e^{-\zeta\omega_n t}[C_1\cos(\omega_d t) + C_2\sin(\omega_d t)]$$

and the forced steady-state response can be expressed as a combination of sine and cosine terms:

$$u_p(t) = A\sin(\Omega t) + B\cos(\Omega t)$$

Therefore, sine and cosine functions naturally appear in the canonical solution of vibration-driven structural response. In the present framework, the harmonic features are not intended to imply that the bolted steering-knuckle response is purely harmonic. Rather, they provide a mechanics-inspired nonlinear basis to help the learning model represent sign-dependent, load-reversal, cyclic or vibration-sensitive response components that may arise under multiaxial service loading.

For the pairwise interaction terms, the motivation comes from the fact that structural responses under combined loads are often not separable into independent single-load contributions. A simple mechanics example is the second order P–Δ effect of a cantilever column subjected to simultaneous axial and lateral loads. As shown in **Response Fig. R2-1(a)**, consider a fixed-base column of height L and rectangular cross-section $H \times W$, with an axial compressive force F_1 and a lateral force F_2 applied at the column top.

For the lateral force alone, the first order bending moment at a distance $x$from the fixed base can be written as:

$$M_1(x) = F_2(L - x)$$

The corresponding top lateral displacement of a cantilever beam is approximately

$$\delta_0 = \frac{F_2 L^3}{3EI}$$

where E is Young's modulus and I is the second moment of area of the cross-section. When the axial compressive load F_1 acts simultaneously with the lateral displacement, it produces an additional second-order moment,

$$M_\Delta(x) = F_1 \delta(x)$$

which is the classical P–Δ contribution in structural stability and beam-column theory **[Response References 5, Response References 6]**, At the fixed base, using the top displacement approximation for an intuitive derivation, the total bending moment can be expressed as:

$$M_{base} \approx F_2 L + F_1 \delta_0 = F_2 L + F_1 (\frac{F_2 L^3}{3EI})$$

Thus,

$$M_{base} \approx F_2 L + \frac{F_1 F_2 L^3}{3EI}$$

This expression explicitly contains the interaction term $F_1 F_2$, showing that the combined structural response cannot always be decomposed into independent contributions from F_1 and F_2. The corresponding bending stress at the fixed base can be written as

$$\sigma_b = \frac{M_{base} c}{I} \approx \frac{F_2 L c}{I} + \frac{F_1 F_2 L^3 c}{3EI^2}$$

where c is the distance from the neutral axis to the extreme fiber. If the axial stress is also included, the total stress can be intuitively expressed as:

$$\sigma = \frac{F_1}{A} + \frac{F_2 L c}{I} + \frac{F_1 F_2 L^3 c}{3EI^2}$$

The first term represents the direct axial-load effect, the second term represents the first-order lateral-bending effect, and the third term represents the coupled axial–lateral interaction effect. This derivation illustrates why pairwise polynomial features such as $x_i x_j$ are mechanically meaningful: they provide the learning model with candidate terms capable of representing force–force, force–moment and moment–moment coupling under multiaxial loading.

To further illustrate this point, we performed a simple finite-element example. A fixed-base steel column with a $2\text{mm} \times 2mm$ cross-section and a height of 20 mm was analyzed under three loading cases. When only a lateral top load of $F_2 = 20$ N was applied, the monitored stress at node 1407 was 231.376 MPa (Response Fig. R2-1(b)). When only an axial

load of $F_1 = 1000$ N was applied, the corresponding stress was 245.667 MPa (Response Fig. R2-1(c)). However, when $F_1 = 1000$ N and $F_2 = 20$ N were applied simultaneously, the stress at the same node increased to 736.313 MPa (Response Fig. R2-1(d)). The combined stress response was therefore much larger than the direct sum of the two single-load responses, $231.376 + 245.667 = 477.043$ MPa, indicating that the simultaneous axial–lateral loading generated an additional interaction-induced stress amplification.

This combined response is not a simple scalar addition of the two separate responses. It reflects the combined influence of load interaction, geometric amplification, local stress redistribution and the nonlinear nature of equivalent stress measures. This example provides an intuitive structural-mechanics explanation for including pairwise interaction terms in the augmented feature space.

Overall, the feature families used in the proposed framework can be mapped to mechanics phenomena as follows: raw load features represent first-order load effects; squared terms represent magnitude-dominated nonlinear effects; pairwise products represent multiaxial coupling and secondary load amplification; and sine/cosine terms represent vibration-inspired, sign-dependent or cyclic response components. Thus, the feature augmentation is mechanics-inspired rather than purely data-driven, and it provides the model with physically interpretable candidate terms for learning nonlinear multiaxial structural responses.

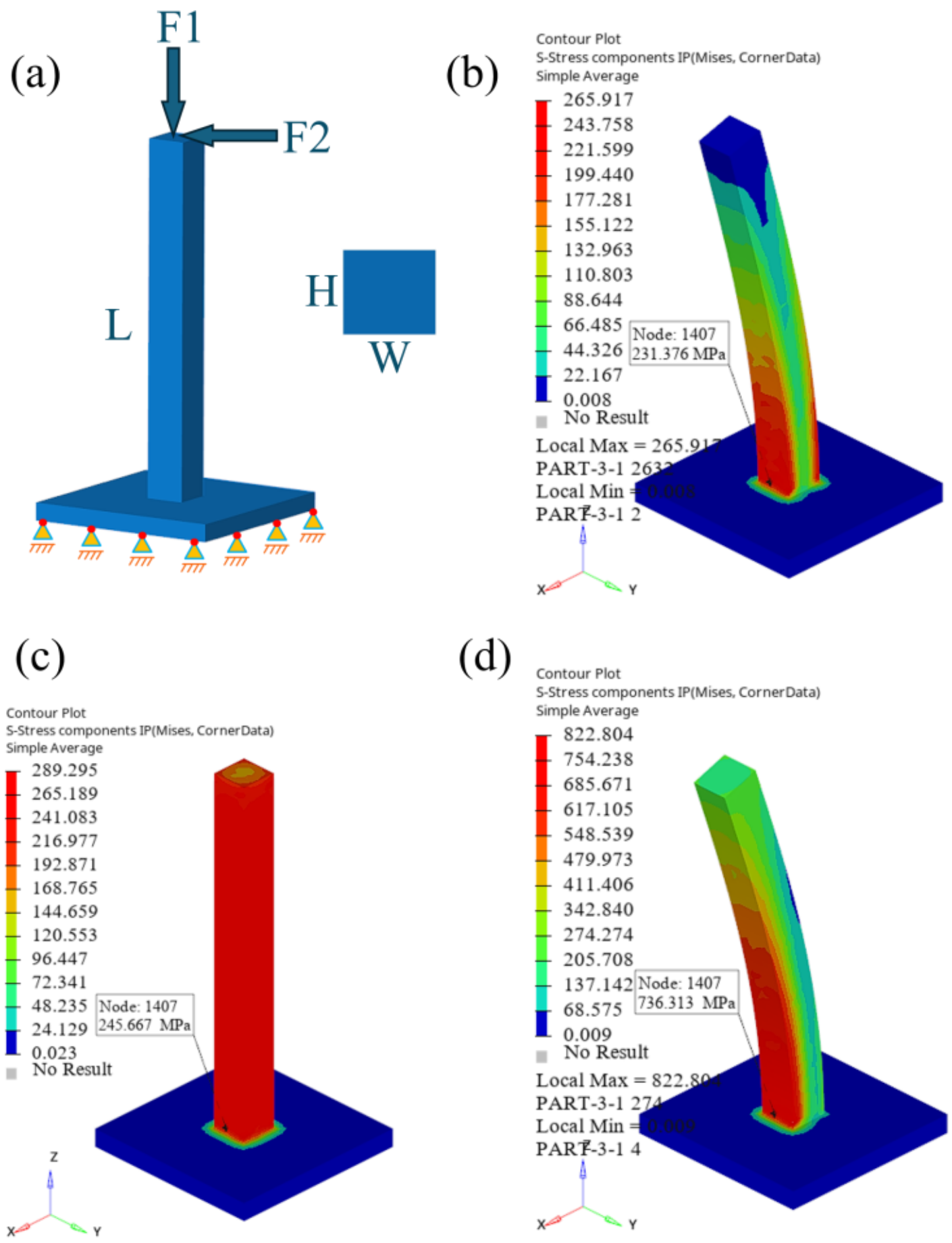

Response Fig. R2-1. Illustrative example for the mechanical meaning of interaction features. (a) Fixed-base steel column subjected to axial load F_1 and lateral load F_2. **(b)** Stress response under lateral load only, $F_2 = 20$ N, with monitored node stress of 231.376 MPa. **(c)**

Stress response under axial load only, $F_1 = 1000\ \mathrm{N}$, with monitored node stress of 245.667 MPa. **(d)** Stress response under combined axial and lateral loads, $F_1 = 1000\ \mathrm{N}$and $F_2 = 20\ \mathrm{N}$, with monitored node stress increasing to 736.313 MPa. The combined response is not a simple scalar addition of the two separate responses, illustrating the need for interaction features in multiaxial structural prediction.

Q2. Check Eq 2 for both positive and negative cases.

Response: We agree that the meaning of positive and negative values in the load-bound expansion equation should be clarified. In the original manuscript, this equation was labelled as Eq. (2); after restructuring the manuscript, the corresponding equation is now labelled as Eq. (4) in the revised Methods section. The force and moment components used in this study are signed Cartesian vector components rather than unsigned load magnitudes.

In the multibody-dynamics load extraction, each force or moment component, such as F_x, F_y, F_z, M_x, M_y, and M_z, represents a directional component along the corresponding Cartesian axis. Therefore, a negative value does not indicate an invalid or "negative load magnitude"; rather, it indicates that the force or moment acts along the negative direction of the corresponding axis. Similarly, a positive value indicates loading along the positive direction of that axis.

For example, during different vehicle events, such as forward driving and backward driving, or left-turning and right-turning conditions, the dominant load path at a given transfer point may reverse direction. In such a case, the same load component may be negative in one event and positive in another. If, for a certain load point, $F_x$is negative during a backward-driving event and positive during a forward-driving event, then the admissible sampling interval for this component naturally spans from a negative value to a positive value. A newly sampled value $\tilde{F}_x$ within this interval therefore retains a clear physical meaning: $\tilde{F}_x < 0$ represents a force component acting along the negative x-direction, whereas $\tilde{F}_x > 0$ represents a force component acting along the positive x-direction.

The purpose of Eq. (4) was therefore to preserve the signed directional range observed from the MBD-derived baseline cases and to allow a limited expansion of that physically admissible range. This is important because the learning model should be exposed not only to different load magnitudes, but also to directional changes in the multiaxial force–moment space. The signs of the sampled components are thus retained as part of the mechanical input information.

To avoid possible misunderstanding, we have clarified this point in Supplementary Method 2. We now state explicitly that the sampled load components are signed Cartesian force–moment components, and that positive and negative values represent opposite physical directions rather than different validity categories. We also clarified that the statistical expansion was performed component-wise within the MBD-derived directional load bounds, so that the generated samples remain physically interpretable.

Q3. Could the degradation value be negative in some case? If yes, how to address this?

Response: We agree that the definition and treatment of negative degradation values should be clarified. In the physical interpretation of bolt pretension degradation, a negative

degradation value does not represent meaningful structural damage. The pretension-degradation index is intended to quantify the loss of bolt preload after loading and unloading. Therefore, when the residual pretension is lower than the initial pretension, the degradation value is positive and represents pretension loss. In contrast, if a computed or predicted value is less than zero, it does not indicate "negative damage"; rather, it indicates that no pretension loss has occurred, or that the response is associated with a very small degradation state.

This distinction is especially important in the data-driven prediction stage. In finite-element-based mechanical evaluation, pretension loss is extracted from the physical preload–load–unload process and is interpreted as a non-negative degradation quantity. However, regression models may occasionally predict slightly negative degradation values because their outputs are continuous and not automatically constrained by physical bounds. Such negative predictions should therefore be corrected before being used in the Degradation Risk Score.

To address this issue, we revised the DRS formulation in the Methods section. In the original manuscript, the bolt-level degradation component was defined directly as the maximum pretension loss across the four bolts. In the revised manuscript, we explicitly constrain the pretension-degradation component to the physically meaningful interval $[0, 1]$. The revised formulation is

$$D_{\mathrm{pre},i} = \mathrm{clip}[\max_{j=1,2,3,4} \left(D_{\mathrm{pre},j}\right)_i, 0, 1]$$

where $\left(D_{\mathrm{pre},j}\right)_i$ is the fractional pretension degradation of bolt j in sample i, and $\mathrm{clip}[\cdot, 0,1]$ restricts the value to the interval $[0,1]$.

With this treatment, any predicted pretension-degradation value below zero is interpreted as no detectable pretension loss and is set to zero before DRS calculation. Values greater than one are also capped at one to prevent non-physical degradation values from entering the risk score. This ensures that the DRS remains a normalized vulnerability indicator bounded between 0 and 1.

We also applied the same bounded logic to the stress-margin degradation component. In the revised manuscript, $D_{\mathrm{res},i}$ is defined as

$$D_{\mathrm{res},i} = \begin{cases} 0, & \sigma_{crit,i} < \sigma_y \\ \frac{\sigma_{crit,i} - \sigma_y}{\sigma_u - \sigma_y}, & \sigma_y \le \sigma_{crit,i} \le \sigma_u \\ 1, & \sigma_{crit,i} \ge \sigma_u \end{cases}$$

so that stress states below yield strength do not generate negative degradation, and stress states beyond ultimate strength are capped at full degradation.

Accordingly, the final DRS is computed as

$$DRS_i = 1 - \left(1 - D_{res,i}\right)\left(1 - D_{pre,i}\right)$$

which guarantees

$$0 \le DRS_i \le 1$$

These revisions clarify that the DRS is calculated only from physically meaningful, bounded degradation indicators.

Q4. Authors should comment on possible overfitting issues.

Response: We agree that possible overfitting should be explicitly discussed, especially

because the proposed feature augmentation increases the input dimension and the learning task is based on a finite FE-simulated dataset.

In the revised manuscript and Supplementary Information, we clarified that overfitting was considered from both the load-case generation level and the predictive-model level. At the load-case level, statistical load-space expansion was used to reduce the dependence of the learning models on a small set of typical MBD-derived service events. The additional load cases were generated by randomized sampling within an MBD-derived physically admissible load envelope and were subsequently evaluated using finite-element simulation, rather than by interpolating response labels. This strategy exposes the models to broader combinations of multiaxial force–moment inputs and reduces the risk that the model simply memorizes a limited set of representative operating cases. The detailed training–testing split and overfitting considerations are now provided in Supplementary Method 5.3.

At the predictive-model level, we also considered overfitting control during model training. Cross-validation, regularization and dropout-based strategies were used or considered according to the model architecture. For example, regularization terms help constrain overly complex tree-based boosting models, ensemble averaging in RF reduces variance, and dropout or related regularization strategies can reduce excessive co-adaptation of neural-network parameters. These model-level controls help prevent the learning algorithms from fitting noise or case-specific fluctuations rather than the underlying multiaxial load–response relationship.

In addition, we evaluated possible overfitting through the comparison between training and testing performance using seven metrics, including RMSE, MSE, MAE, MAPE, RAE, R^2, and residual mean offset. The metric definitions are provided in Table S5. These metrics jointly quantify error magnitude, relative error, goodness of fitness and residual bias, rather than relying only on R^2. Fig. 2 presents a bubble-plot comparison of training and testing performance across baseline and FA/RA-enhanced models. Because the bubble size represents the normalized magnitude of each metric, similar bubble patterns between the training and testing sets indicate that the testing performance remains consistent with the training behavior. As shown in Fig. 2, the FA-RA MLR and FA-RA BP-NN models exhibit broadly similar bubble patterns between the training and testing sets, indicating that the trained models retain predictive capability on unseen samples and that the risk of severe overfitting is limited.

Table S5

Mathematical formulation of each evaluation metric.

Evaluation metrics	Expression
RMSE (Root Mean Squared Error)	$RMSE = \sqrt{\frac{1}{N}\sum_{i=1}^{N}(y_i - \hat{y}_i)^2}$
MSE (Mean Squared Error)	$MSE = \frac{1}{N}\sum_{i=1}^{N}(y_i - \hat{y}_i)^2$
MAE (Mean Absolute Error)	$MAE = \frac{1}{N}\sum_{i=1}^{N}\lvert y_i - \hat{y}_i\rvert$
MAPE (Mean Absolute Percentage Error)	$MAPE = 100\% \times \frac{1}{N}\sum_{i=1}^{N}\frac{\lvert y_i - \hat{y}_i\rvert}{\lvert y_i\rvert}$

RAE (Relative Absolute Error)	$RAE = \frac{\sum_{i=1}^{N}\lvert y_i - \hat{y}_i\rvert}{\sum_{i=1}^{N}\lvert y_i - \bar{y}\rvert}$
R^2 (Coefficient of Determination)	$R^2 = 1 - \frac{\sum_{i=1}^{N}(y_i - \hat{y}_i)^2}{\sum_{i=1}^{N}(y_i - \bar{y})^2}$
RMO (Residual mean offset)	$RMO = \lvert u_r\rvert, u_r = \text{argmax } [p(r)]$ $p(r) = \frac{1}{\sigma\sqrt{2\pi}} exp\left(-\frac{(r - u_r)^2}{2\sigma^2}\right)$

Here, y_i and $\hat{y}_i$ denote the FEA and predicted responses, respectively; $\bar{y}$ is the mean FEA response; N is the number of samples; and $r = y_i - \hat{y}_i$ is the residual.

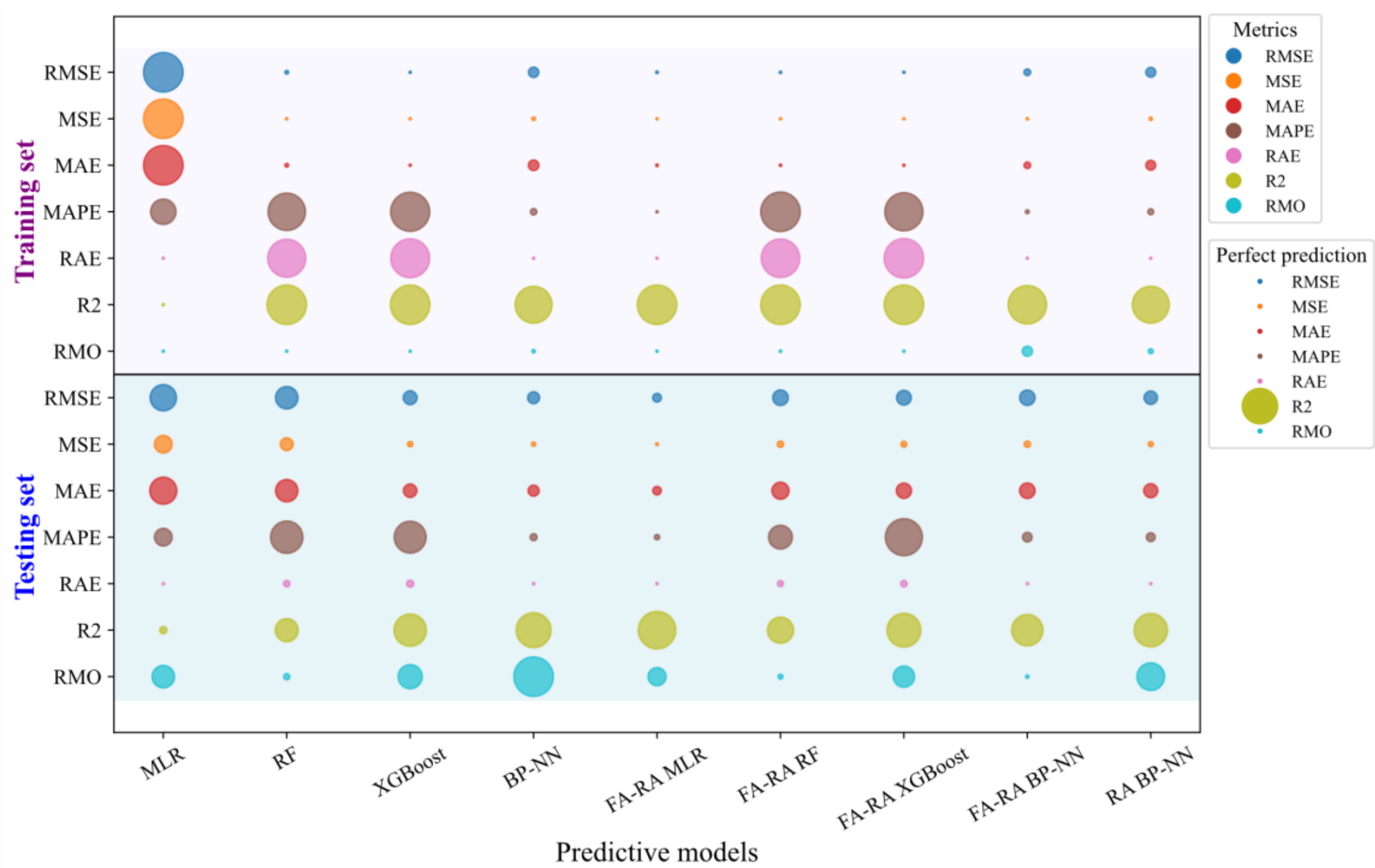

Fig. 2. Predictive performance benchmarking across model families. Bubble plot comparing training and testing performance of baseline and FA/RA-enhanced models using RMSE, MSE, MAE, MAPE, RAE, R^2, and residual mean offset (RMO). Bubble size represents the normalized magnitude of each metric. Lower values are preferred for RMSE, MSE, MAE, MAPE, RAE, and residual mean offset, whereas higher values are preferred for R^2.

Q5. Clearly mention the parameter values for reproducibility.

Response: We agree that reproducibility is important for evaluating and reusing the proposed framework. In the revised manuscript, we have clarified where the key information required for reproducing the study can be found.

First, the finite-element model, the original multibody-derived load cases, the statistically expanded load samples, and the corresponding FE-simulated response results are provided through the "Data availability" section. These data include the multiaxial load inputs, the peak von Mises stress responses, and the bolt pretension-degradation values used for model training and evaluation.

Second, the revised Methods section now provides a clearer description of the training targets and modelling procedure, including the multiaxial load extraction, statistical load-space

expansion, mechanics-informed feature augmentation, Weibull-based risk-aware learning, predictive model configurations, evaluation metrics, and DRS calculation. The sample-splitting and overfitting considerations are further described in Supplementary Method 5.3, and the evaluation metric definitions are provided in Table S5.

Third, the code supporting the findings of this work is available from the corresponding authors upon reasonable request. This includes the scripts used for data preprocessing, feature construction, model training, prediction evaluation, and risk-score calculation. We have clarified this point in the "Code availability" statement.

These revisions and availability statements provide the necessary data, methodological details, and code access for reproducing the main results of the study.

Q6. Authors may like to elaborate on the assumed form for DRS.

Response: We agree that the assumed form of the Degradation Risk Score (DRS) should be explained more clearly. In the revised manuscript, we clarified that DRS is not treated as a directly calibrated probability of failure, but as a normalized degradation-based vulnerability score. Its purpose is to integrate two mechanically different but coupled degradation pathways: stress-margin degradation in the knuckle and connection-level degradation caused by bolt pretension loss.

The DRS formulation is based on the following expression:

$$DRS_i = 1 - (1 - D_{res,i})(1 - D_{pre,i})$$

Here, $D_{\mathrm{res},i}$ represents the normalized stress-margin degradation of the knuckle, while $D_{\mathrm{pre},i}$ represents the connection-level degradation defined by the maximum pretension loss among the four bolts. We selected the multiplicative complementary form because it has a clear engineering interpretation. The term $1 - D_{res,i}$ can be regarded as the remaining integrity of the stress-margin pathway, and $1 - D_{pre,i}$ can be regarded as the remaining integrity of the bolted-connection pathway. Their product therefore represents the simultaneous preservation of both pathways. Taking the complement gives the combined vulnerability induced by either stress-margin loss, pretension degradation, or both.

The key intuition is that the structure remains in a low-vulnerability state only when both degradation pathways retain sufficient integrity; degradation in either pathway increases the DRS, and simultaneous degradation in both pathways produces a stronger increase. This is why the multiplicative form is more appropriate than a simple average for the present purpose. For example, if either the stress margin is strongly degraded or one bolt experiences severe pretension loss, the risk score should increase even if the other pathway remains moderate. If both pathways degrade simultaneously, the score should increase more strongly to reflect compound degradation.

This form also has useful mathematical properties for risk ranking. First, because both $D_{\mathrm{res},i}$ and $D_{\mathrm{pre},i}$ are bounded in $[0,1]$, the resulting DRS is also bounded in $[0,1]$. Second, the score increases when either degradation component becomes more severe. In other words, a larger stress-margin degradation or a larger bolt pretension loss will not reduce the DRS; it will either increase the score or keep it unchanged. Third, the formulation avoids prescribing subjective weighting coefficients between material overstress and connection degradation. This is important because, in the absence of sufficient component-level failure labels, assigning fixed empirical weights to the two degradation pathways would introduce additional assumptions.

We also clarified the relation between DRS and Bayesian calibration. In the main workflow, DRS is used as a deterministic vulnerability score for sample-wise ranking rather than as a calibrated failure probability. When inspection, testing, or monitoring observations become available, DRS or its components D_{res} and D_{pre} can be mapped to component-level failure probability through Bayesian logistic or Bayesian Weibull calibration, as detailed in Supplementary Note 2. This separation avoids treating the assumed DRS form as an already calibrated probability model, while still allowing it to serve as a physically interpretable input for probabilistic reliability assessment.

Response References

[1] Bendiksen O O. Localization phenomena in structural dynamics[J]. Chaos, Solitons & Fractals, 2000, 11(10): 1621-1660.
[2] Craig Jr R R, Kurdila A J. Fundamentals of structural dynamics[M]. John Wiley & Sons, 2006.
[3] Strømmen E N. Structural dynamics[M]. Switzerland: Springer International Publishing, 2014.
[4] Géradin M, Rixen D J. Mechanical vibrations: theory and application to structural dynamics[M]. John Wiley & Sons, 2015.
[5] Timoshenko S P, Gere J M. Theory of elastic stability[M]. Courier Corporation, 2012.
[6] Chen W F, Lui E M. Structural stability: theory and implementation[J]. (No Title), 1987.

Version 1:

Reviewer #1

All of my previous concerns have been satisfactorily addressed by the authors. The manuscript has been revised accordingly, and the changes have improved its clarity and quality. I have no further comments or concerns. In my opinion, the manuscript is now suitable for publication in its present form.

Response: We sincerely thank the reviewer for the careful re-evaluation of our revised manuscript and for the positive assessment of our work. We greatly appreciate the reviewer's recognition that our responses and revisions have satisfactorily addressed the previous concerns and improved the clarity and quality of the manuscript. Your constructive comments during the first round of review were highly valuable in helping us refine the methodological explanations, clarify the scope of the proposed framework, improve the discussion of reliability modeling, and enhance the overall presentation of the manuscript.

Reviewer #2

I am happy with the revision made by the authors. I recommend its publication.

Response: We sincerely thank the reviewer for the careful evaluation of our revised manuscript and for the positive comments regarding our work. We greatly appreciate the reviewer's recognition of the importance of the proposed mechanics-informed, risk-aware learning framework for structural reliability prediction under multiaxial loading. Your insightful comments in the previous round were instrumental in improving the technical rigor, physical interpretation, reproducibility, and presentation quality of the manuscript. We are grateful for the reviewer's constructive suggestions and encouragement throughout the review process.

This paper presents a mechanics-informed, risk-aware machine learning framework for structural reliability prediction under multiaxial loading. The work connects physics-based simulation with data-driven prediction, based on feature augmentation, risk-aware weighting and unified risk scoring. Such development is important for reliability engineering. However, I have the following concerns about their work:

1) Explanations for considering the specific forms of nonlinearity are required to be connected to structural mechanics. Please explicitly map each feature family to a mechanics phenomenon. Explanation with intuition would be very much appreciated.
2) Check Eqn 2 for both positive and negative cases.
3) Could the degradation value be negative in some case? If yes, how to address this?
4) Authors should comment on possible overfitting issues.
5) Clearly mention the parameter values for reproducibility.
6) Authors may like to elaborate on the assumed form for DRS.
