# Supplementary material for: Mechanics-informed risk-aware learning for multiaxial structural reliability with Bayesian calibration
Source: Commun Eng. 2026 Aug 14;5:148. doi: 10.1038/s44172-026-00752-y (PMC13476256; doi:10.1038/s44172-026-00752-y)
Supplement: Supplementary file 2 [file 44172_2026_752_MOESM2_ESM.pdf]

Supplementary Information for

Mechanics-Informed Risk-Aware Learning for Multiaxial Structural Reliability with Bayesian Calibration

This file includes:

- Supplementary Figures S1-S12
- Supplementary Tables S1-S5
- Supplementary Methods 1-5
- Supplementary Notes 1-2
- Supplementary Discussion 1
- Supplementary References 1-13

Supplementary Figures.

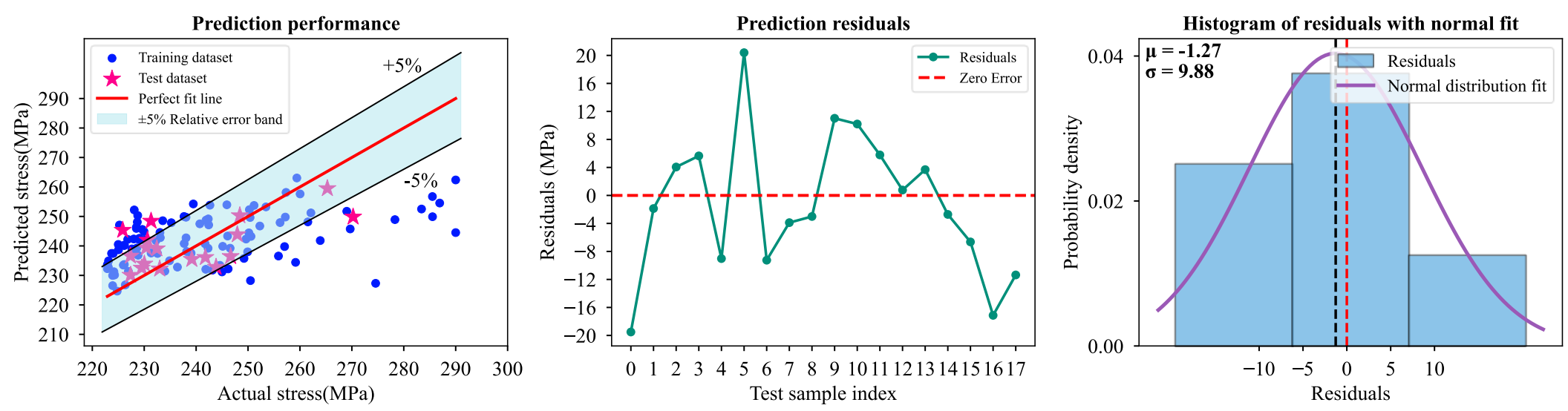

(a)

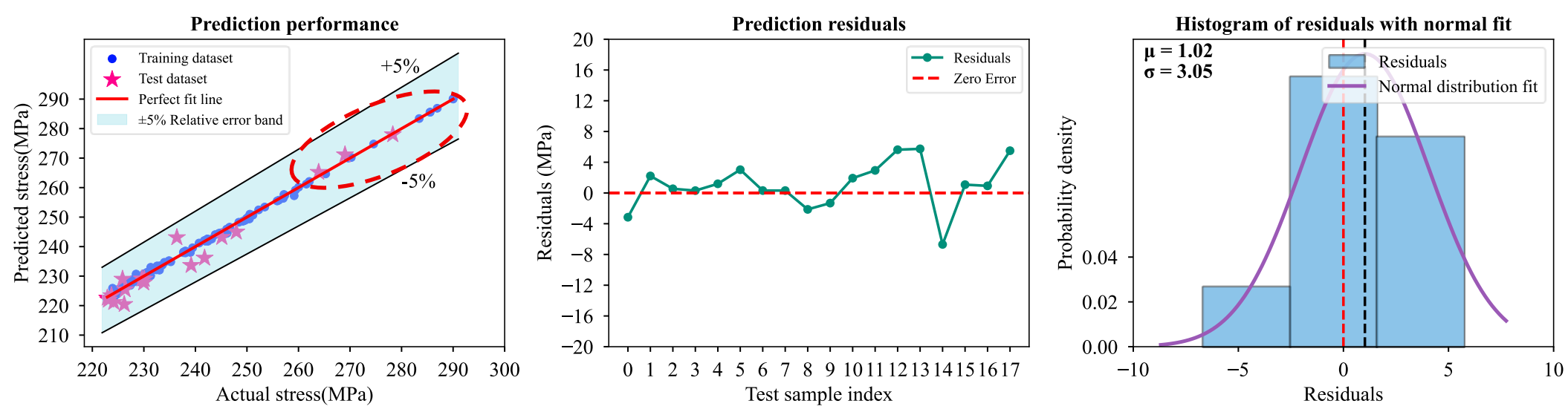

(b)

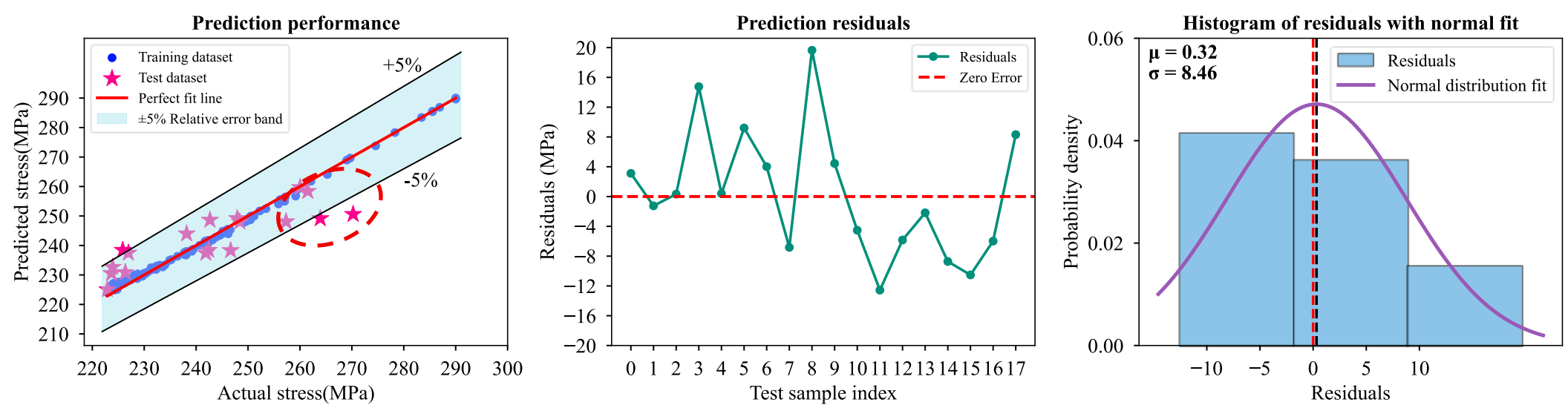

(c)

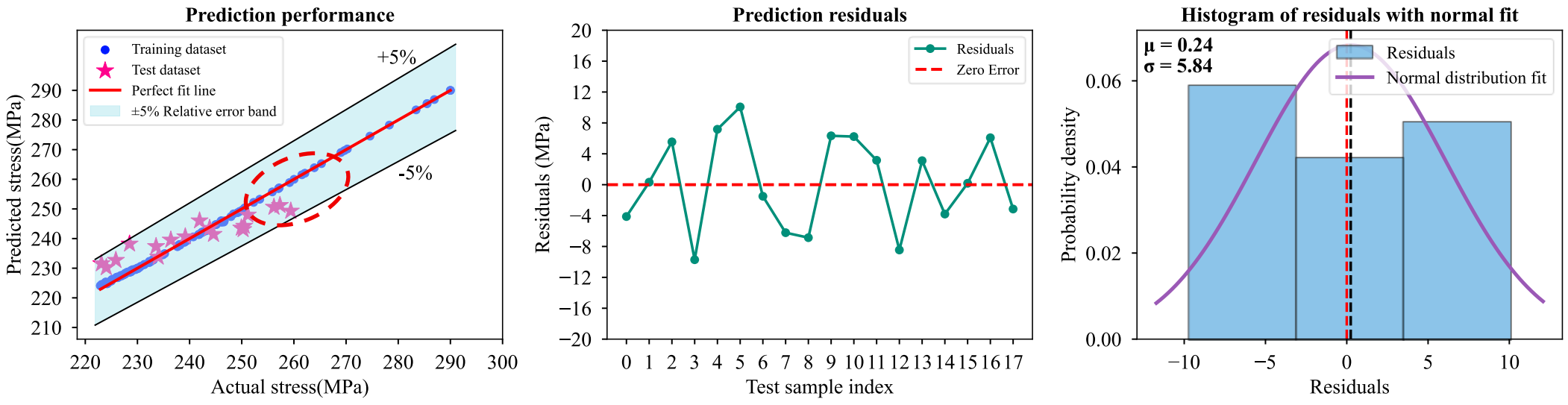

Prediction performance
Training dataset
Test dataset
Perfect fit line
±5% Relative error band
+5%
-5%
Predicted stress(MPa)
Actual stress(MPa)
Prediction residuals
Residuals
Zero Error
Residuals (MPa)
Test sample index
Histogram of residuals with normal fit
μ = 0.24
σ = 5.84
Normal distribution fit
Probability density
Residuals

(d)

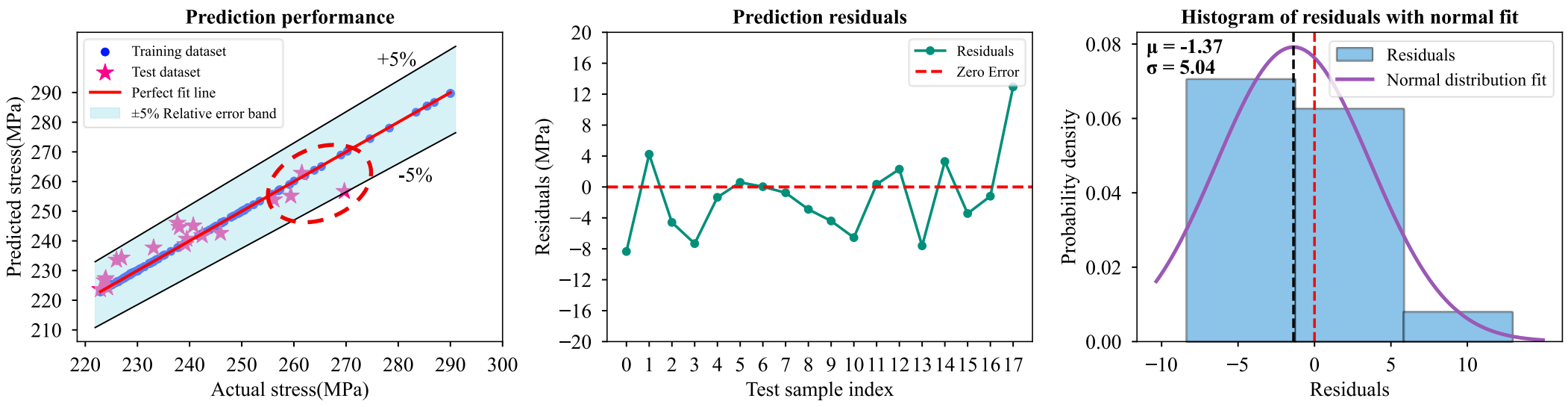

Prediction performance
Training dataset
Test dataset
Perfect fit line
±5% Relative error band
+5%
-5%
Predicted stress(MPa)
Actual stress(MPa)
Prediction residuals
Residuals
Zero Error
Residuals (MPa)
Test sample index
Histogram of residuals with normal fit
μ = -1.37
σ = 5.04
Normal distribution fit
Probability density
Residuals

(e)

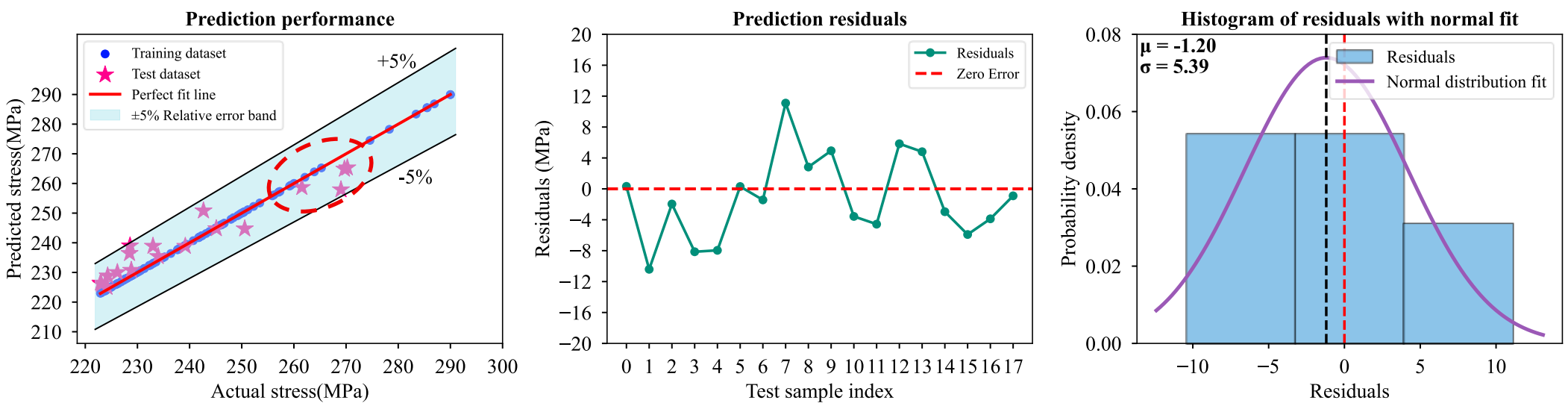

Prediction performance
Training dataset
Test dataset
Perfect fit line
±5% Relative error band
+5%
-5%
Predicted stress(MPa)
Actual stress(MPa)
Prediction residuals
Residuals
Zero Error
Residuals (MPa)
Test sample index
Histogram of residuals with normal fit
μ = -1.20
σ = 5.39
Normal distribution fit
Probability density
Residuals

(f)

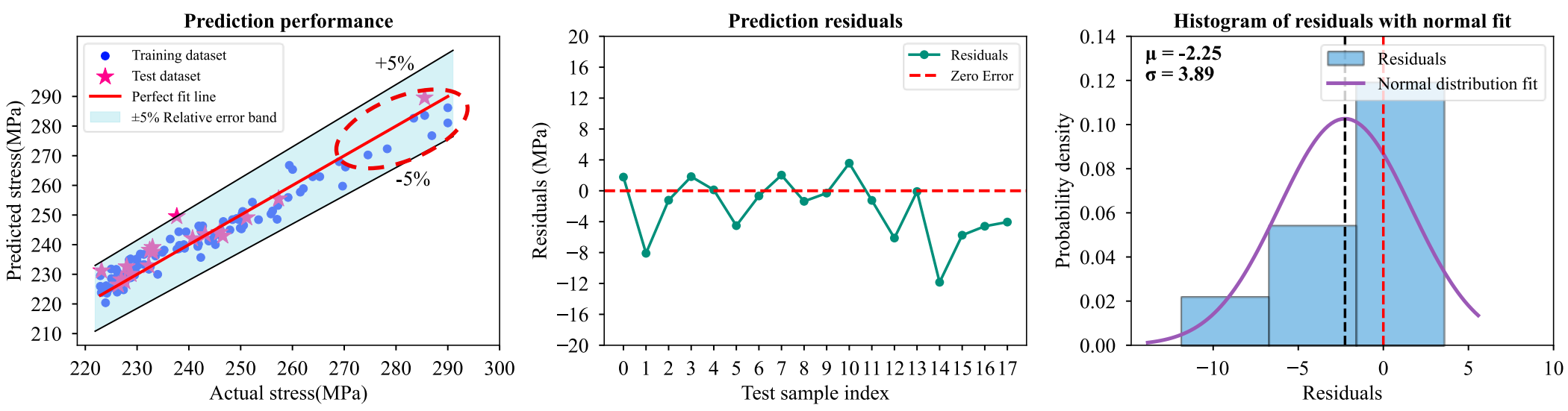

Prediction performance
Training dataset
Test dataset
Perfect fit line
±5% Relative error band
+5%
-5%
Predicted stress(MPa)
Actual stress(MPa)
Prediction residuals
Residuals
Zero Error
Residuals (MPa)
Test sample index
Histogram of residuals with normal fit
μ = -2.25
σ = 3.89
Normal distribution fit
Probability density
Residuals

(g)

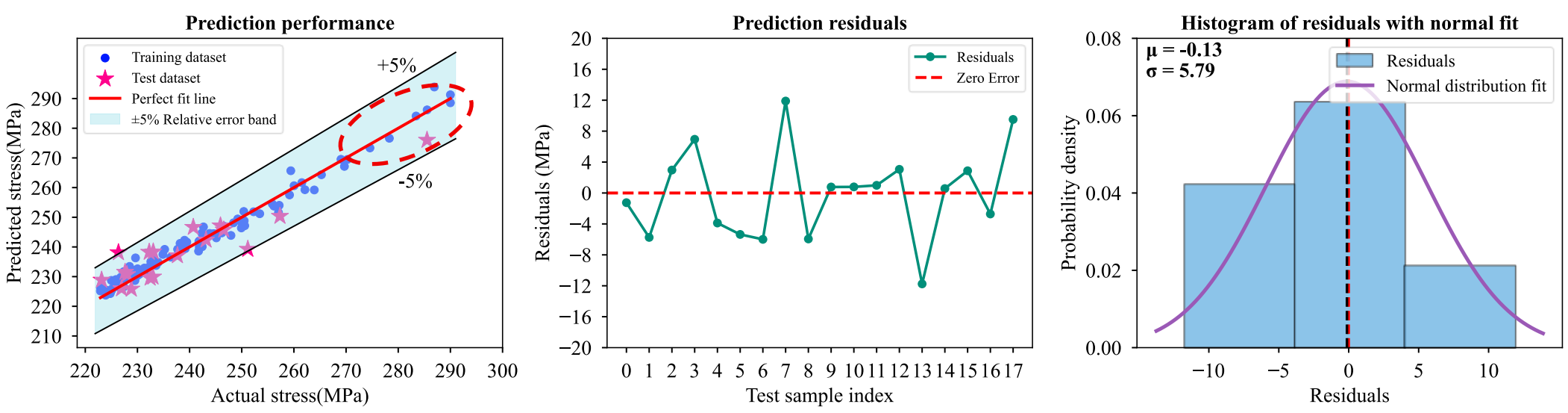

Prediction performance
Training dataset
Test dataset
Perfect fit line
±5% Relative error band
+5%
-5%
Predicted stress(MPa)
Actual stress(MPa)
Prediction residuals
Residuals
Zero Error
Residuals (MPa)
Test sample index
Histogram of residuals with normal fit
μ = -0.13
σ = 5.79
Normal distribution fit
Probability density
Residuals

(h)

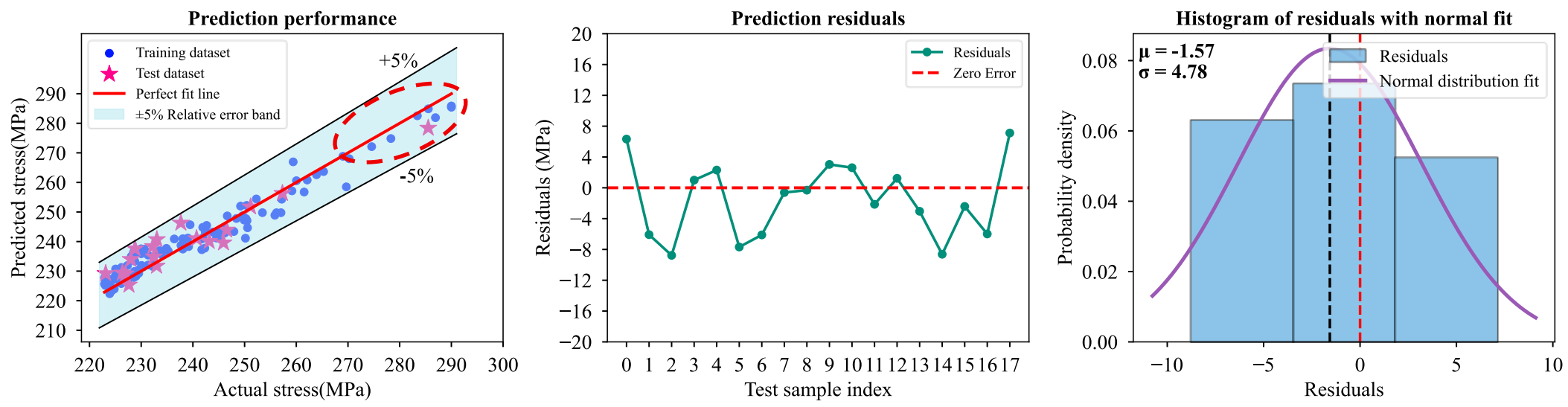

(i)

Fig. S1. Critical-stress prediction and residual diagnostics for all models: Each row shows predicted versus actual stress, testing residuals, and residual distribution with normal fit for one model: (a) MLR; (b) FA-RA MLR; (c) RF; (d) FA-RA RF; (e) XGBoost; (f) FA-RA XGBoost; (g) BP-NN; (h) FA-RA BP-NN; and (i) RA BP-NN.

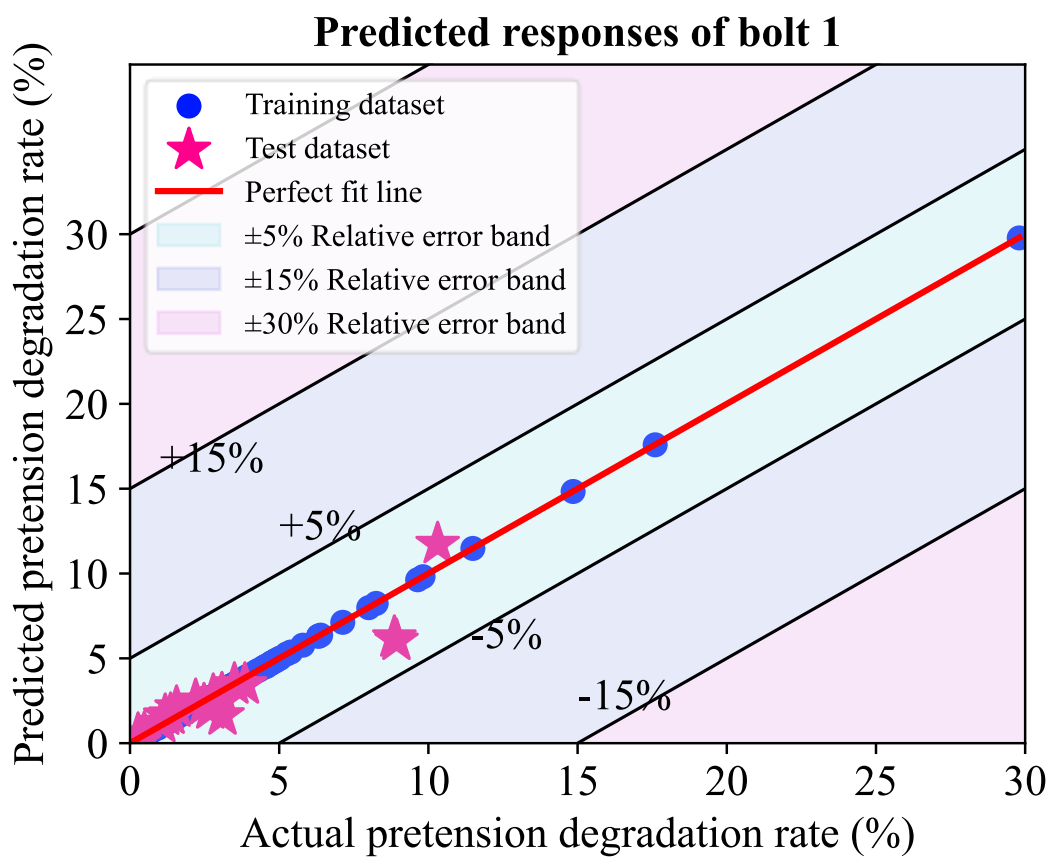

(a)

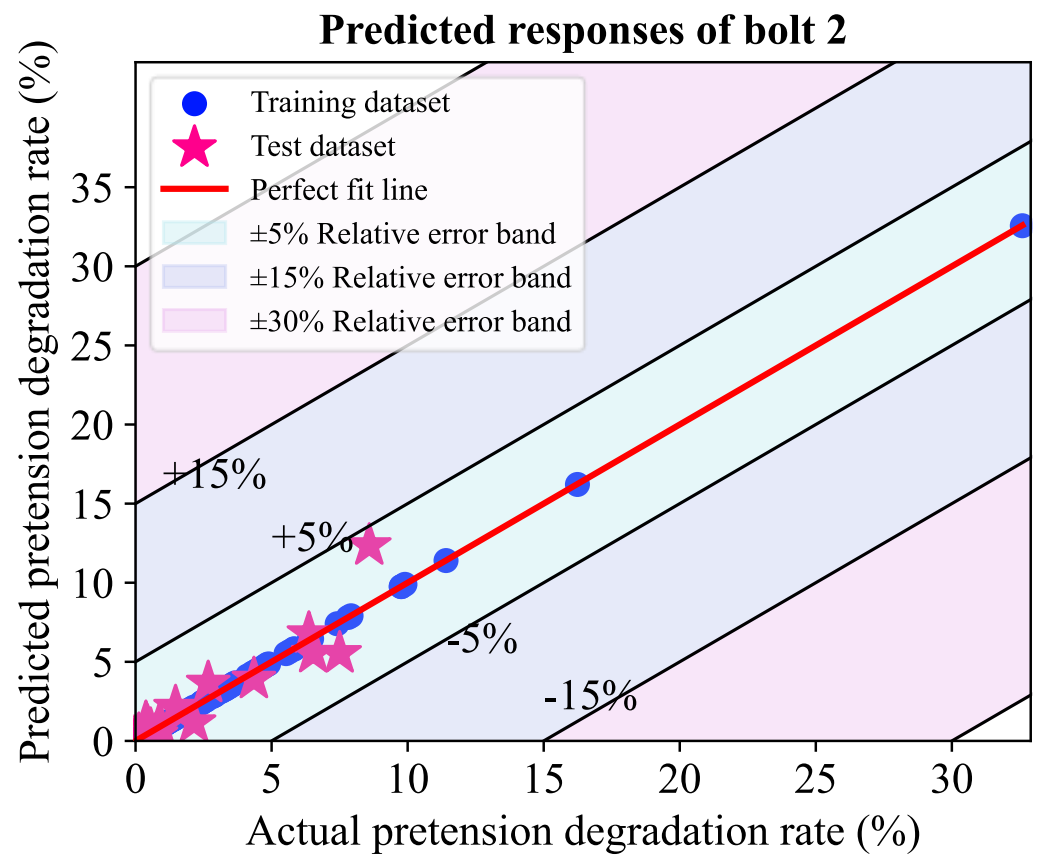

(b)

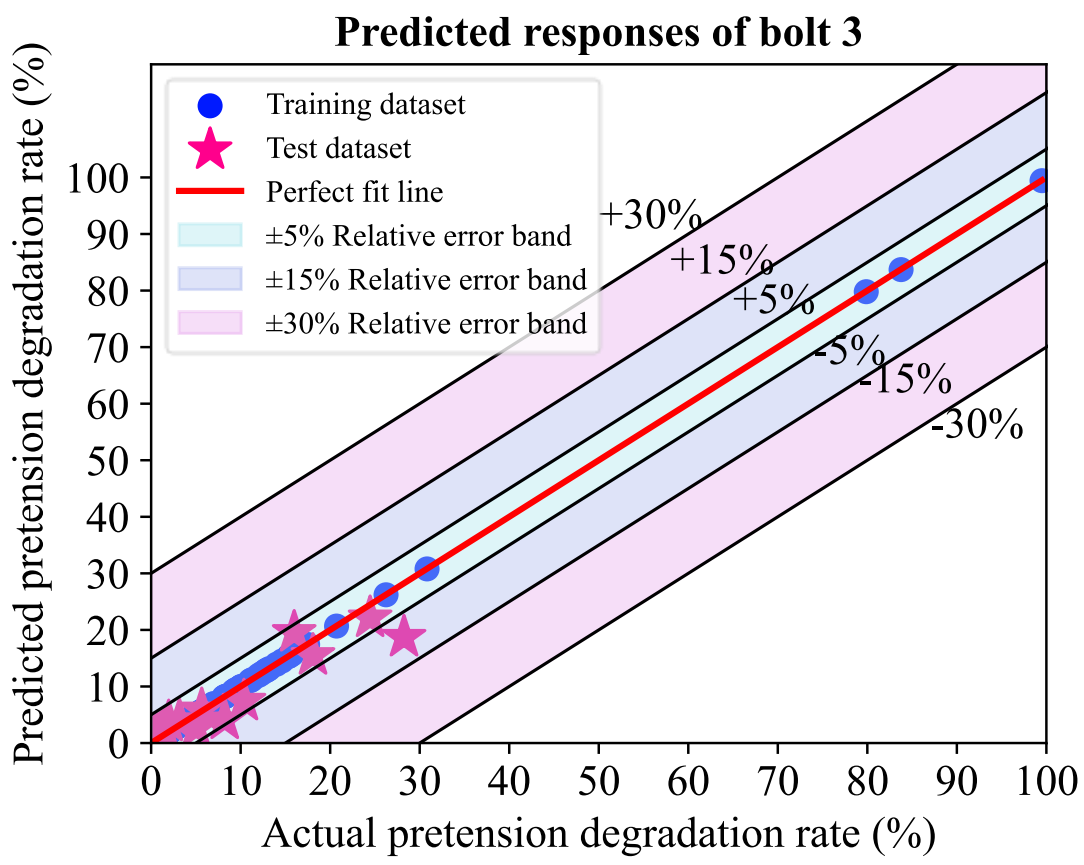

(c)

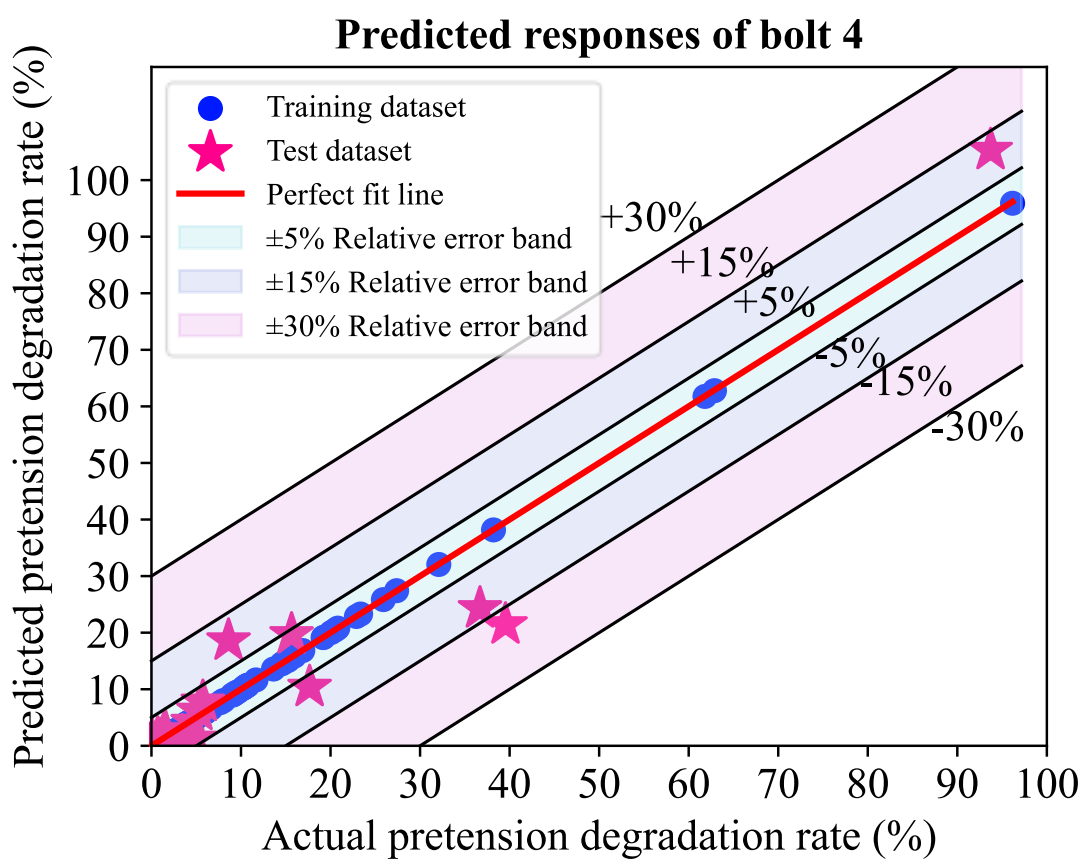

(d)

Fig. S2. Bolt pretension-degradation prediction with relative error bands: Predicted versus actual pretension degradation for (a) bolt 1, (b) bolt 2, (c) bolt 3, and (d) bolt 4. Shaded regions indicate ±5%, ±15%, and ±30% relative error bands.

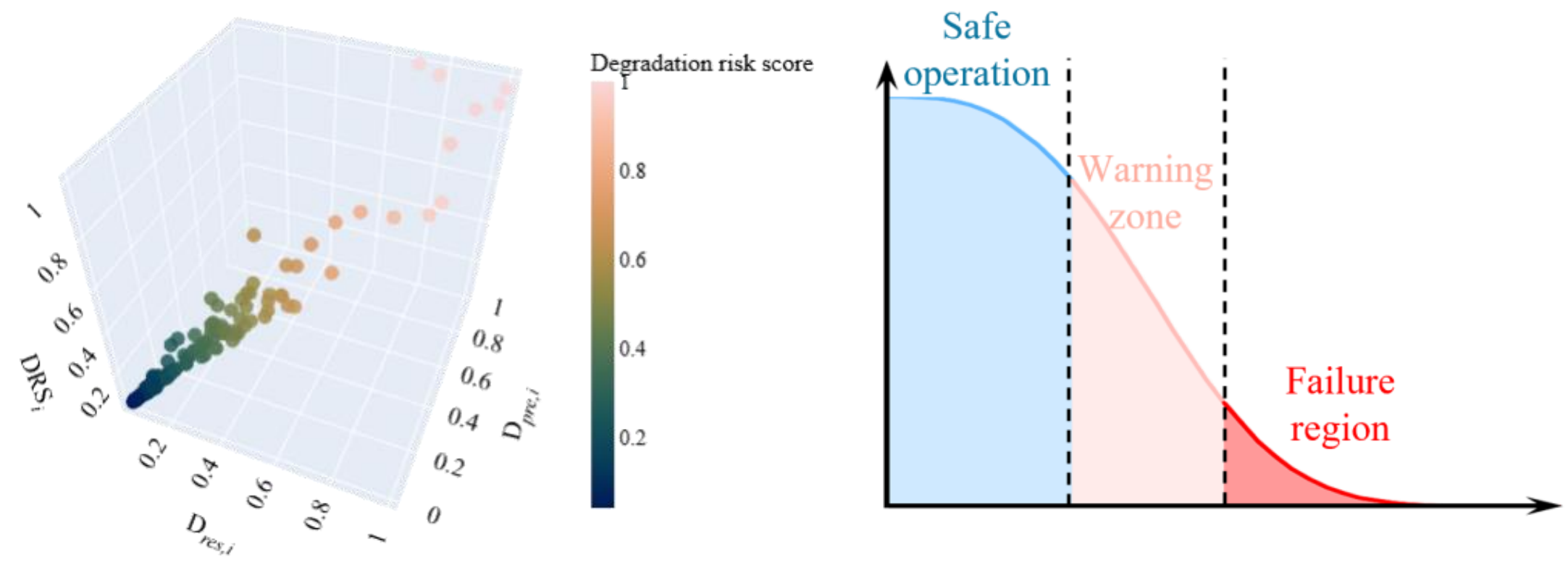

(a) (b)

Fig. S3. Sample-wise degradation risk scores and calibrated structural reliability. (a) Joint distribution of stress-margin degradation $D_{res,i}$, bolt pretension degradation $D_{pre,i}$, and unified DRS DRS_i. (b) Sample-wise and system-level reliability based on Bayesian calibration.

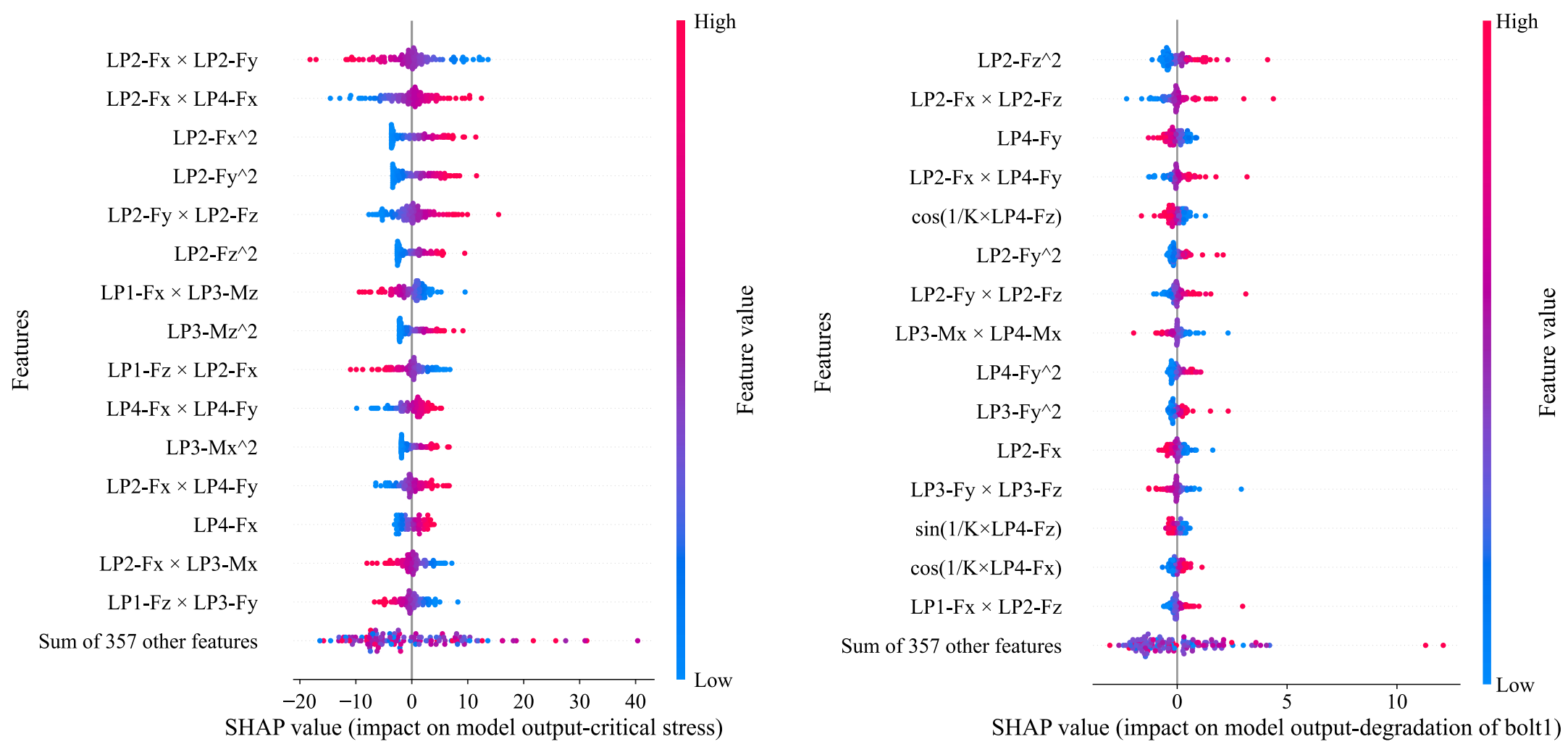

(a) (b)

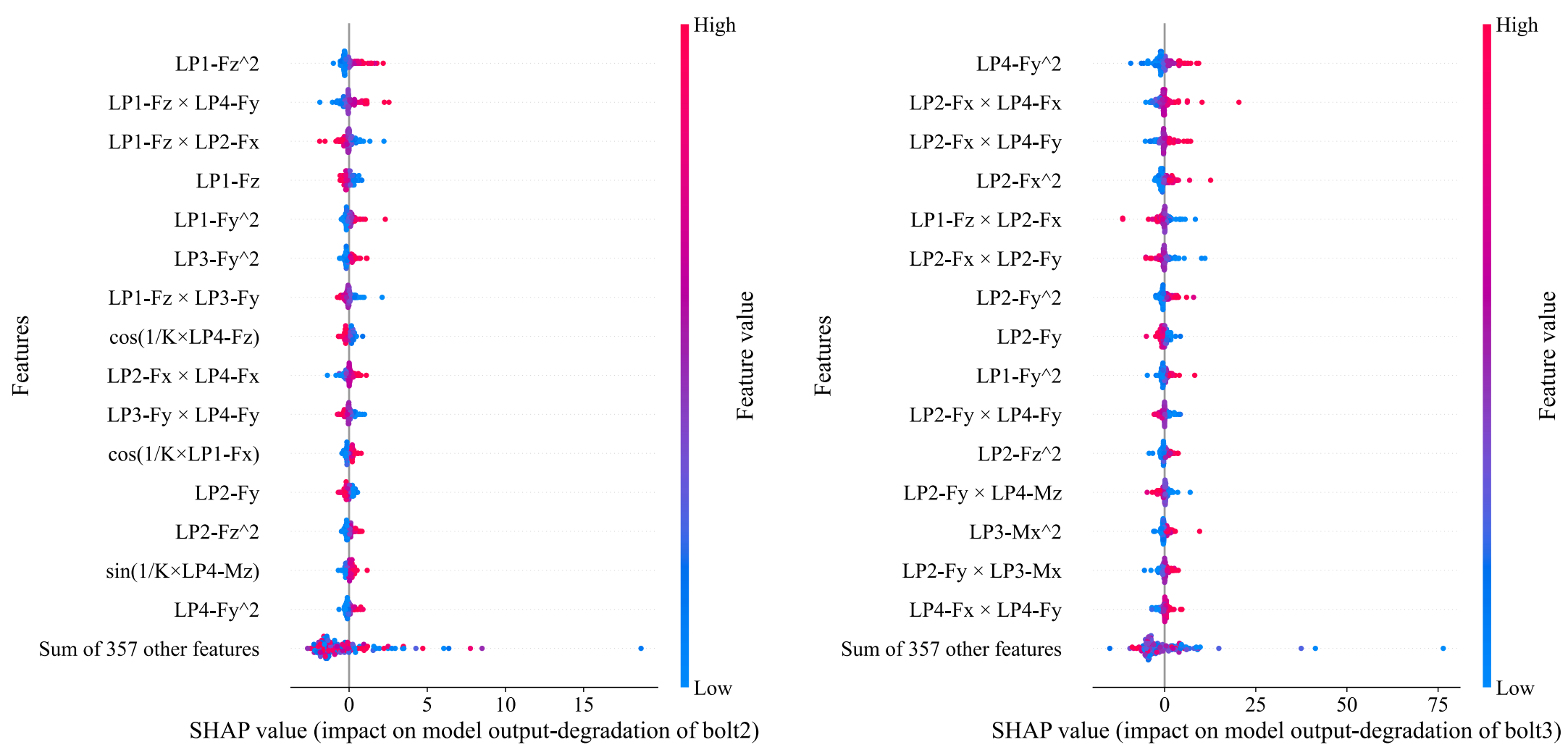

(c) (d)

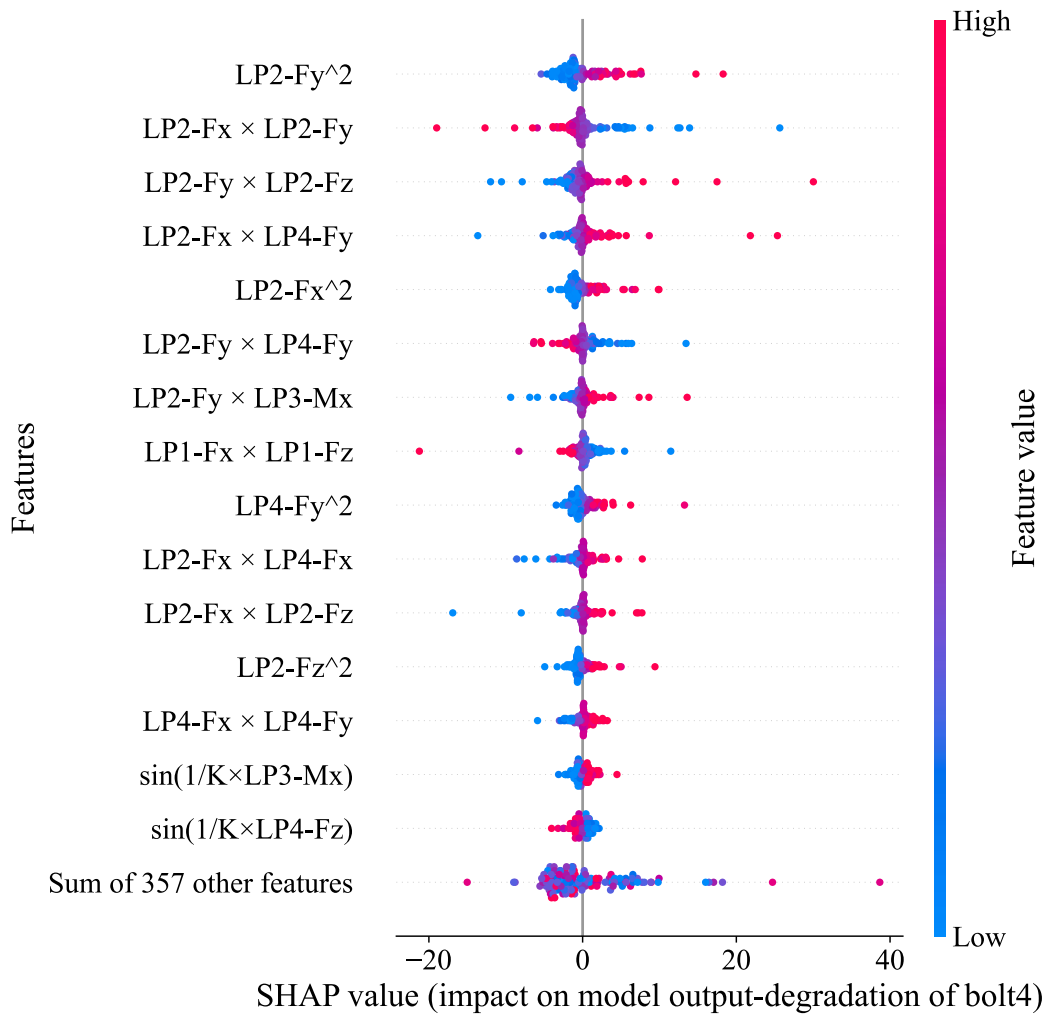

(e)

Fig. S4. SHAP beeswarm plots of the most influential augmented features. The 15 most important predictive features are shown for (a) critical stress and (b–e) bolt pretension degradation of bolts 1–4. Each point represents a sample, and the color denotes the corresponding feature value.

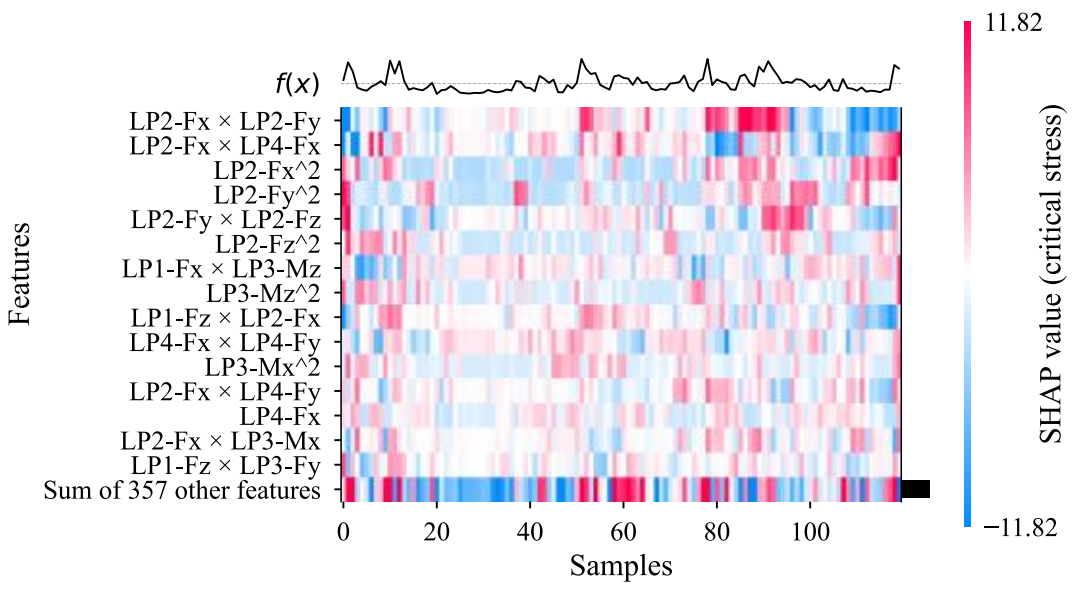

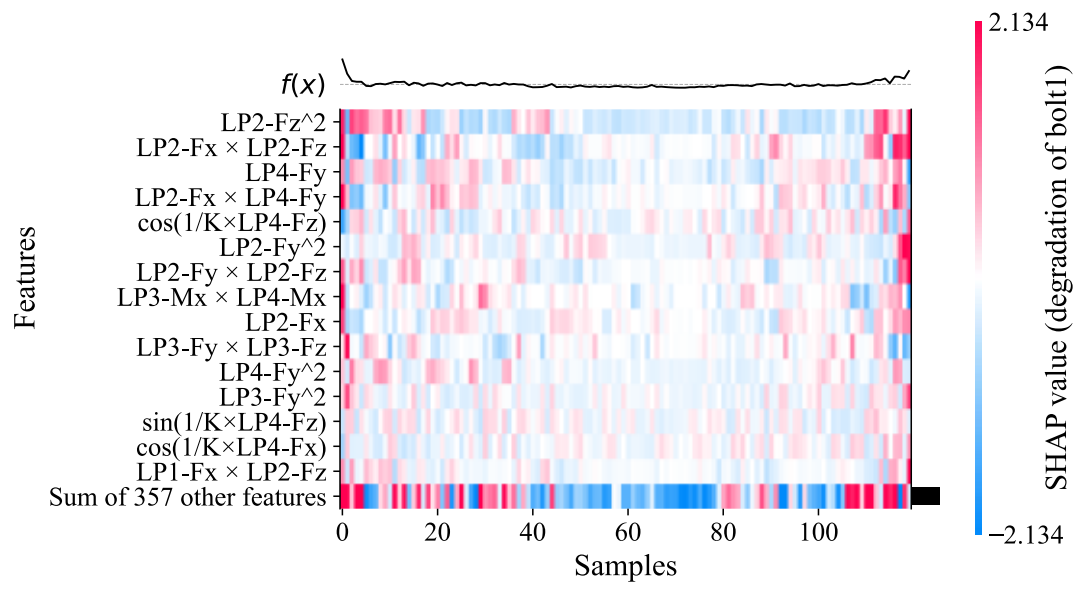

(a)

(b)

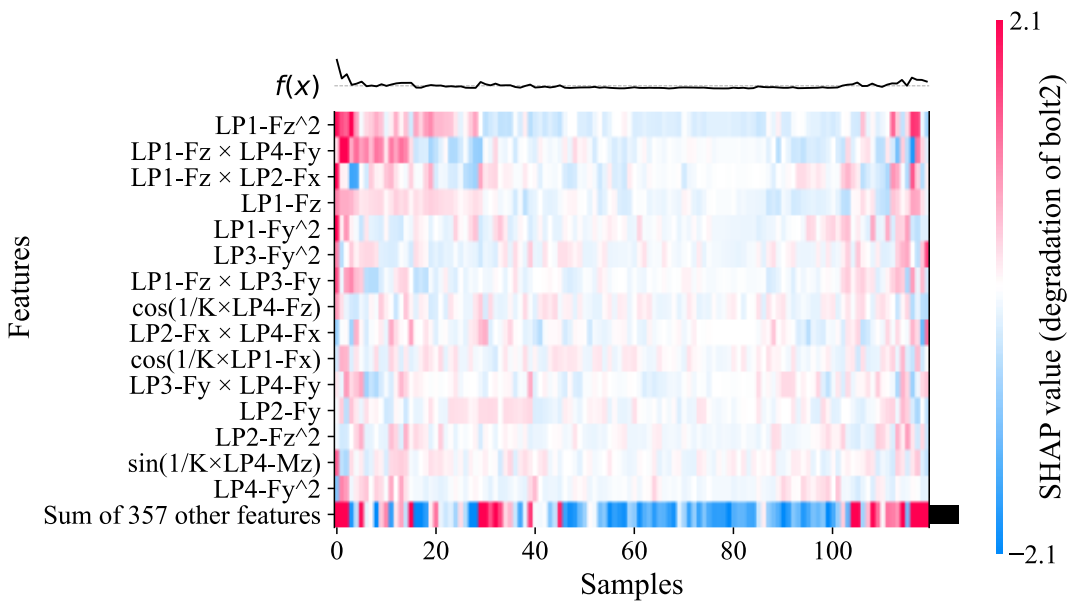

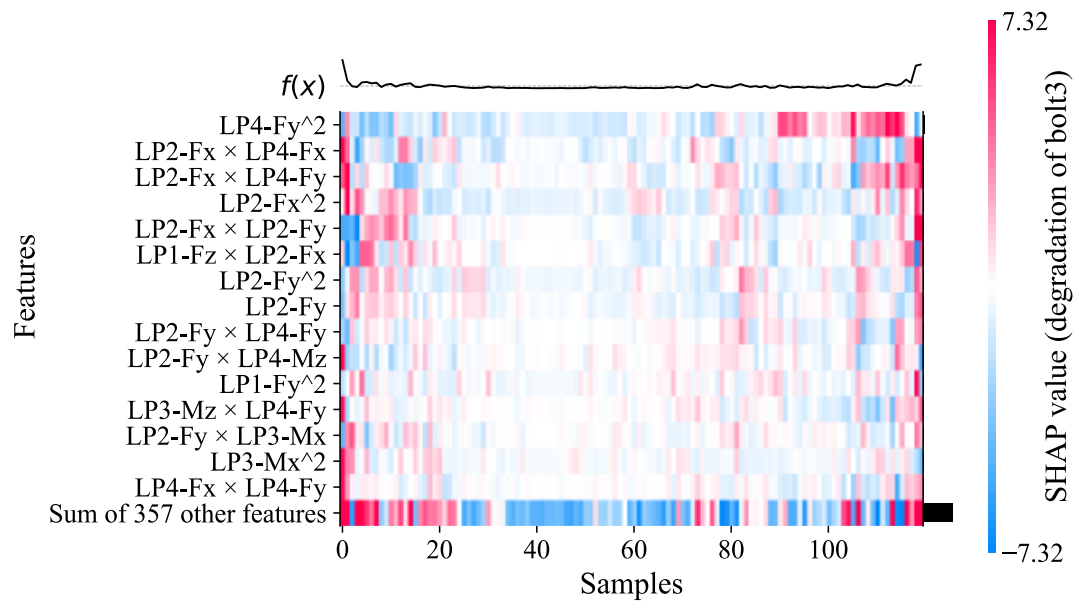

(c)

(d)

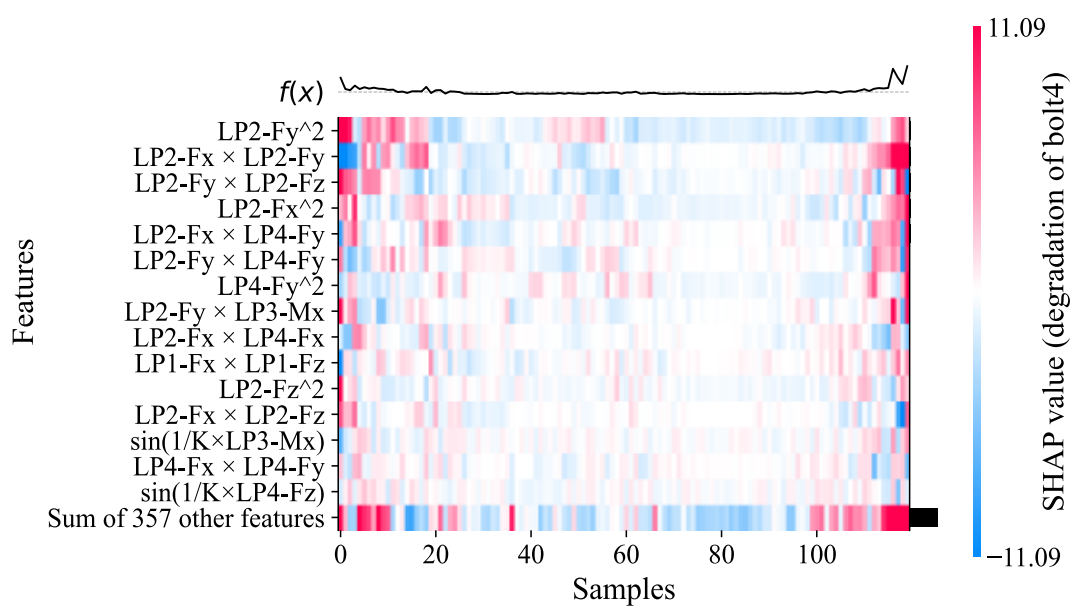

(e)

Fig. S5. Sample-wise SHAP heatmaps of the most influential augmented features. Heatmaps show the sample-level SHAP contributions of the 15 most important features for (a) critical stress and (b–e) bolt pretension degradation of bolts 1–4.

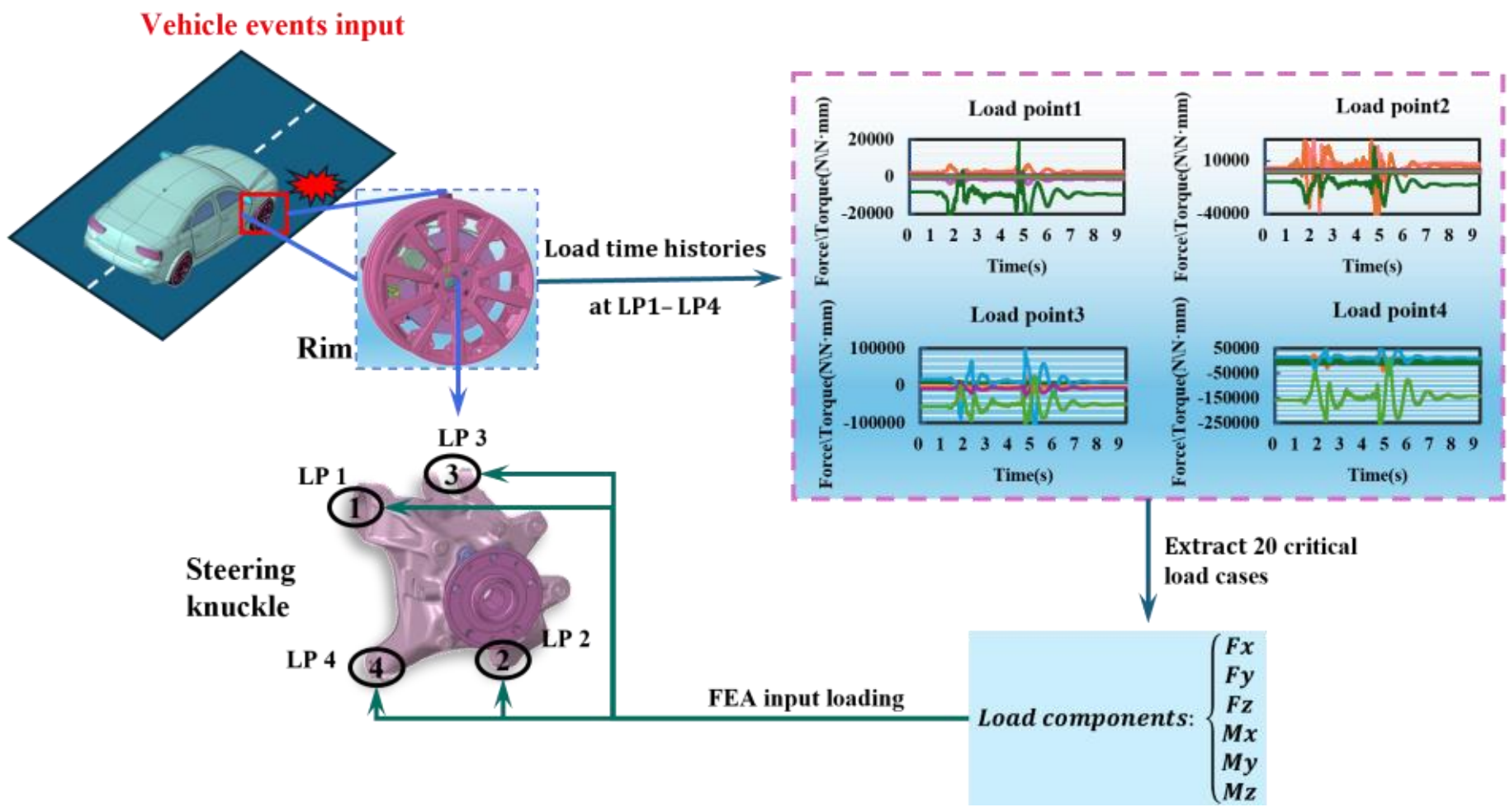

Fig. S6. Multiaxial load acquisition from MBD. Time histories of six-degree-of-freedom force–moment responses were extracted at four load-transfer points LP1–LP4, and mapped to the finite-element load points of the bolted steering-knuckle assembly.

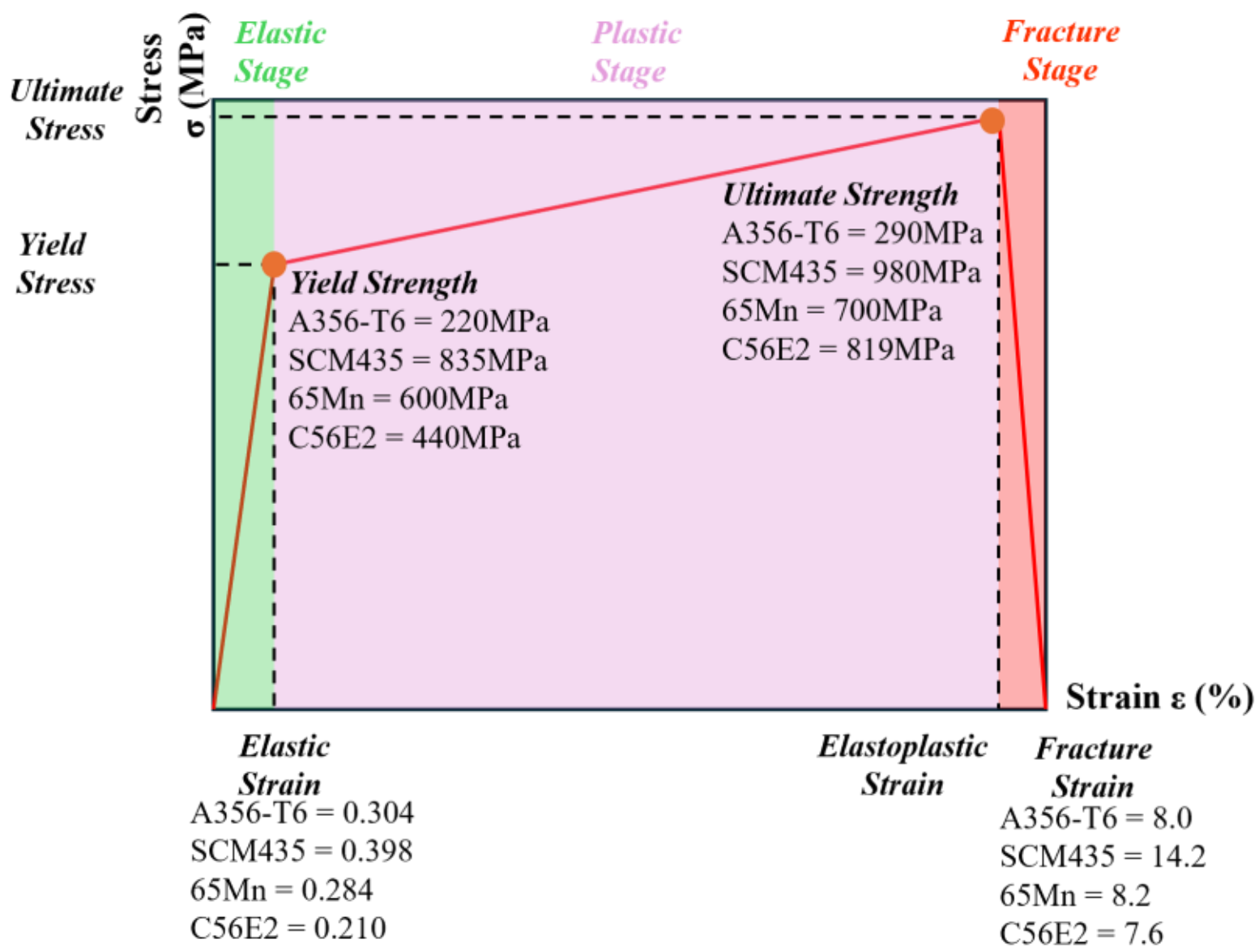

Fig. S7. Constitutive material properties used in FEA. Elastoplastic stress–strain relationships and mechanical properties for A356-T6 aluminum alloy, SCM435 steel, C56E2 steel, and 65Mn steel.

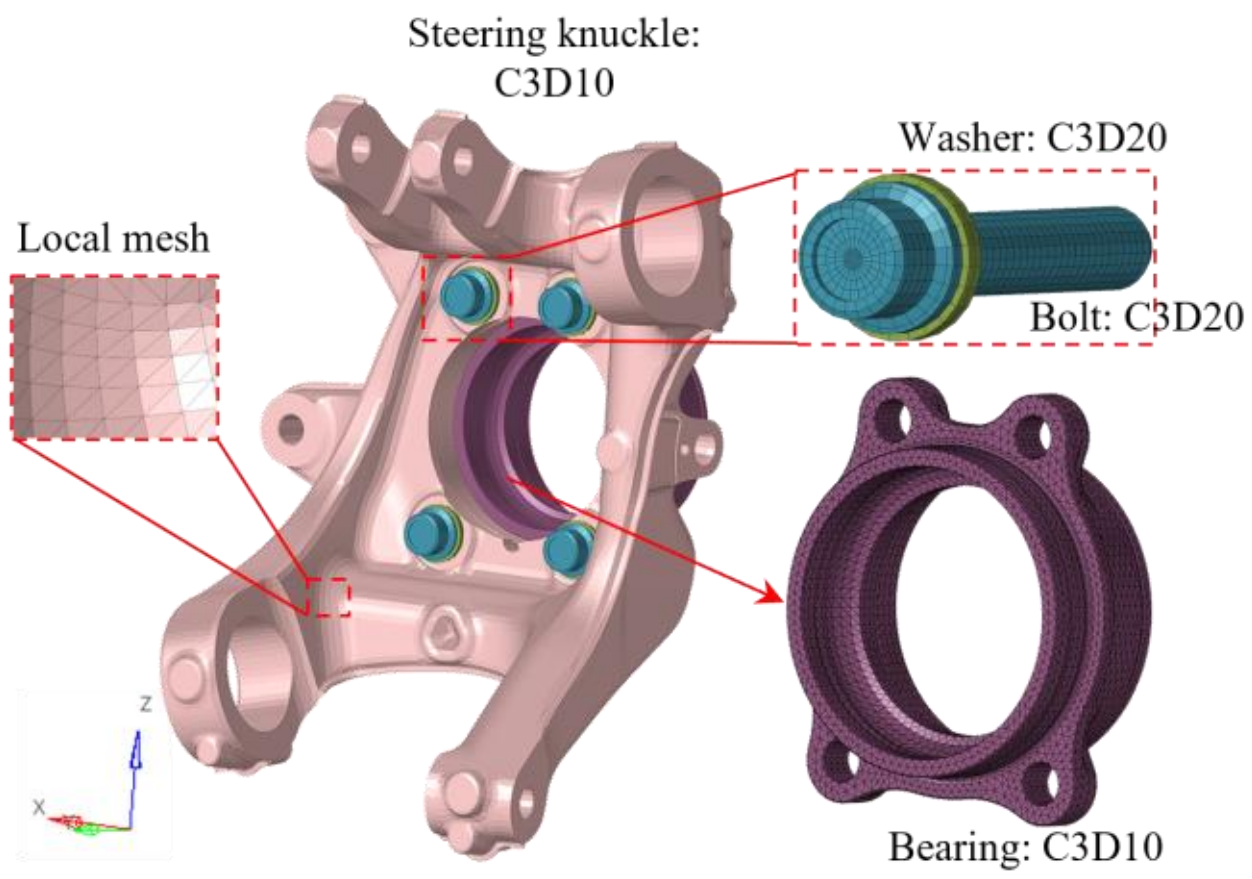

Fig. S8. Finite-element mesh of the bolted steering-knuckle assembly. The model uses C3D10 tetrahedral elements for the knuckle and bearing and C3D20 hexahedral elements for bolts and washers, with local refinement around bolt sections and stress-concentration regions.

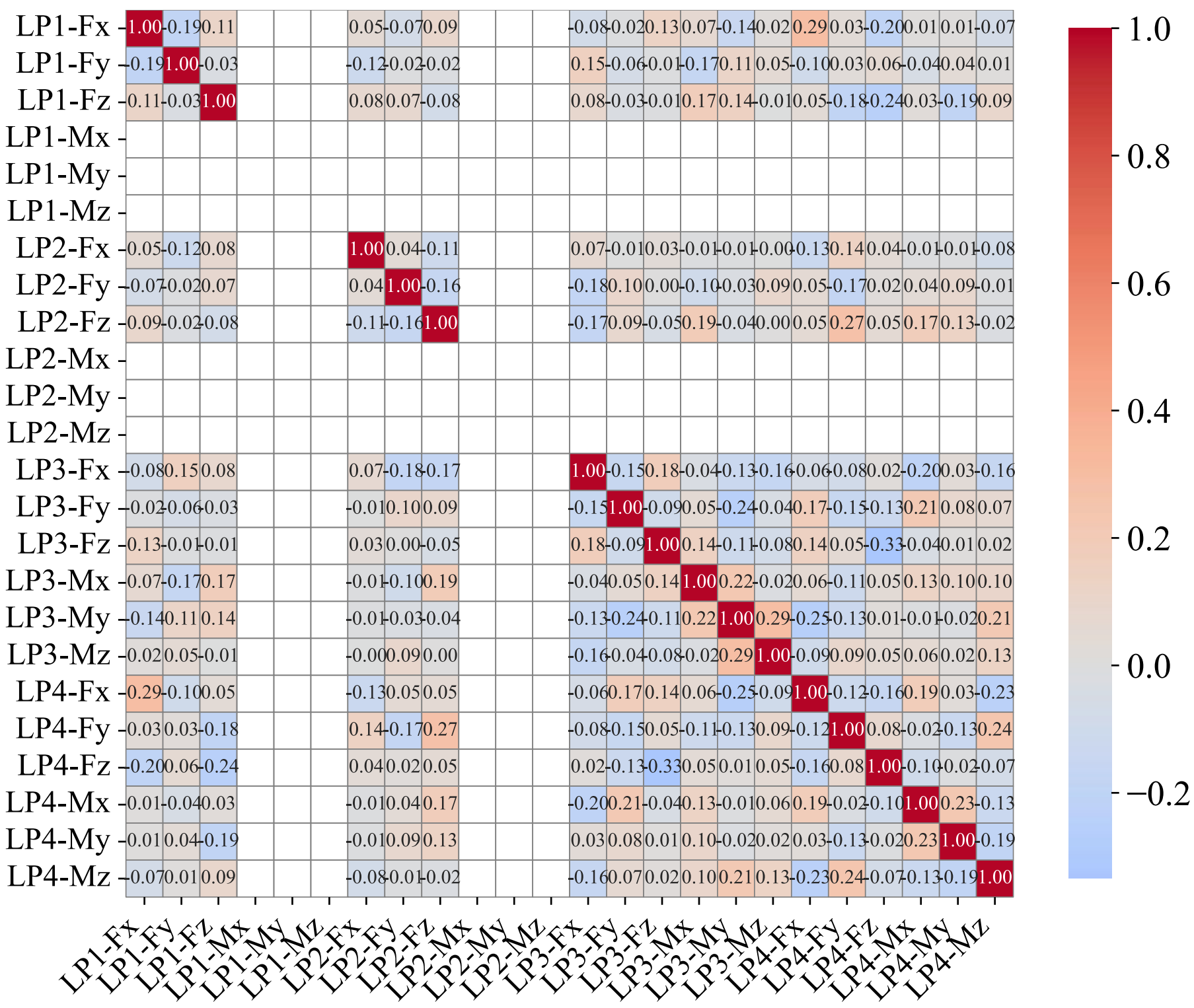

(a)

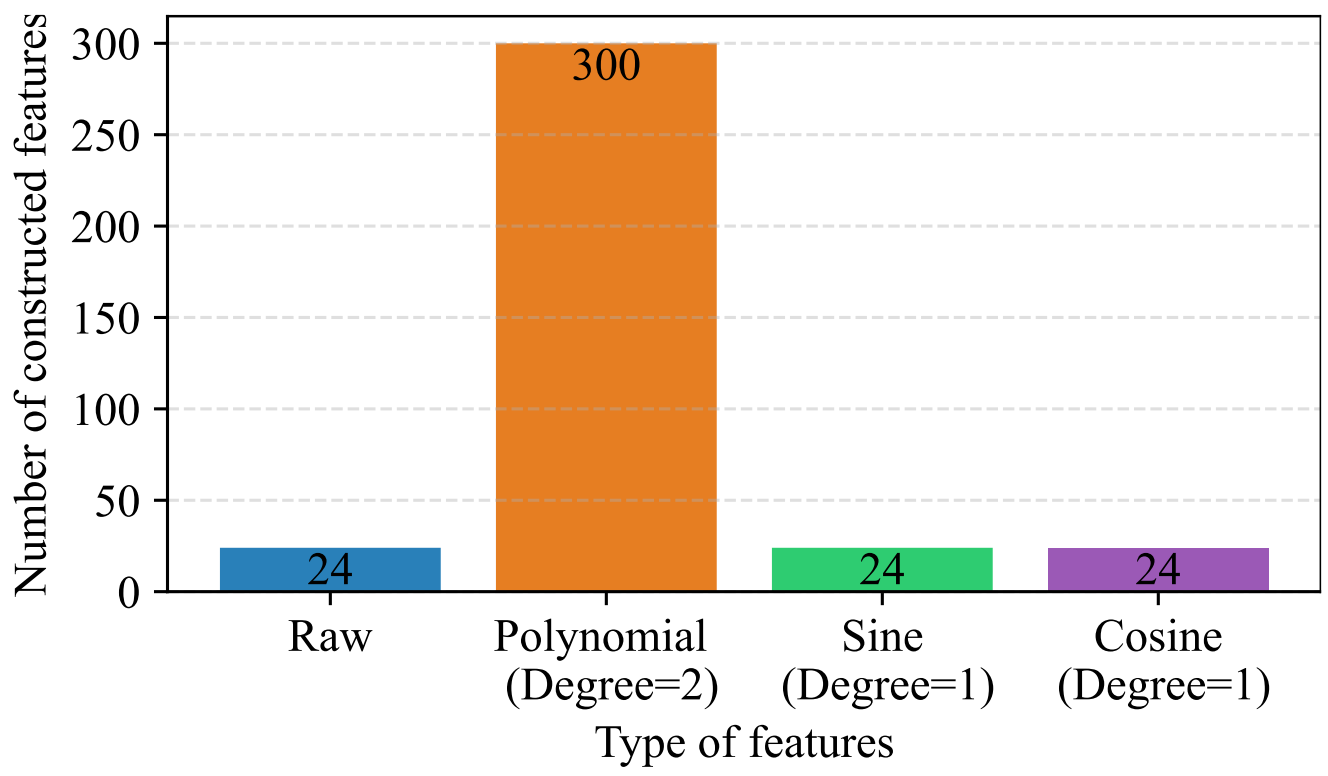

(b)

Fig. S9. Raw input correlation and polynomial–harmonic feature augmentation. (a) Pearson-correlation heatmap of the original 24 load components. Most raw load components show weak pairwise linear correlations, indicating that nonlinear interactions are not sufficiently represented by raw inputs alone. (b) Feature-space expansion from 24 raw load features to 372 augmented features, including second-order polynomial terms, sine terms, and cosine terms for nonlinear interaction representation.

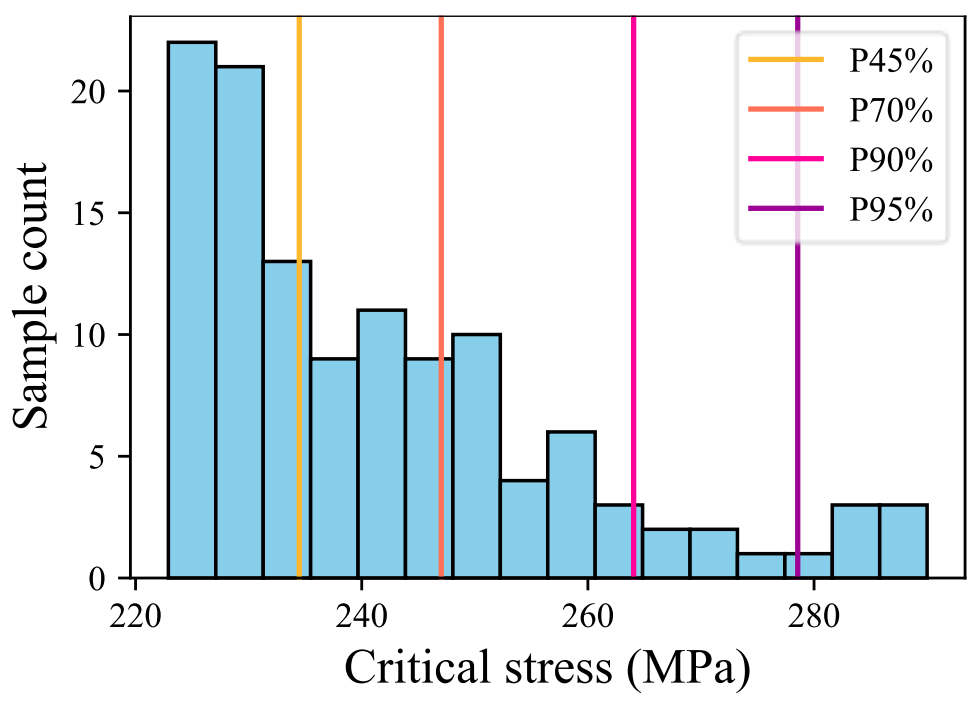

(a)

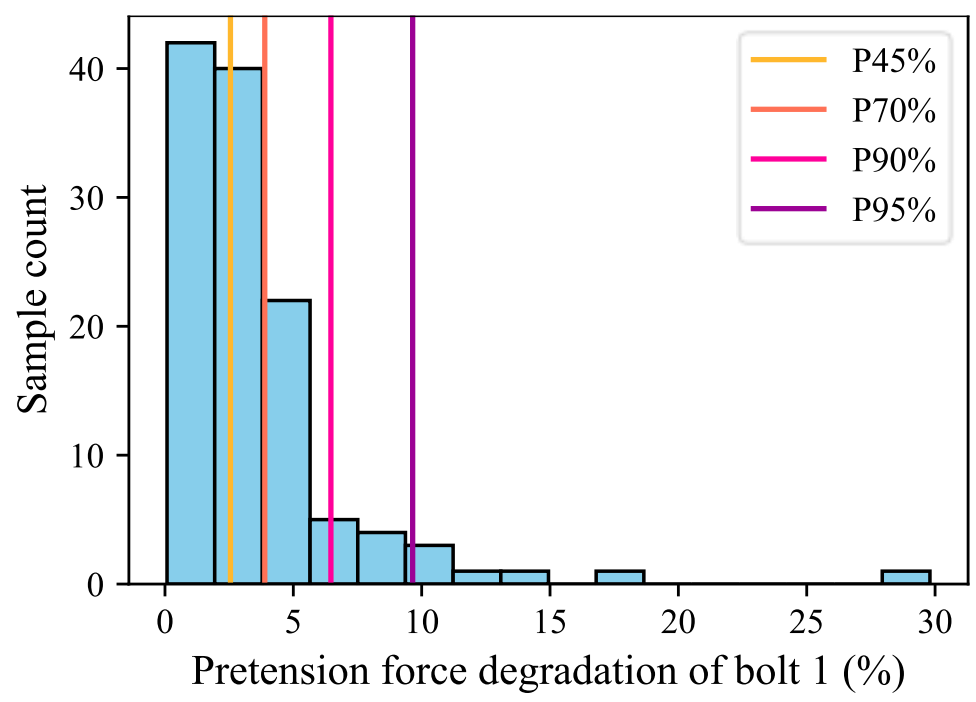

(b)

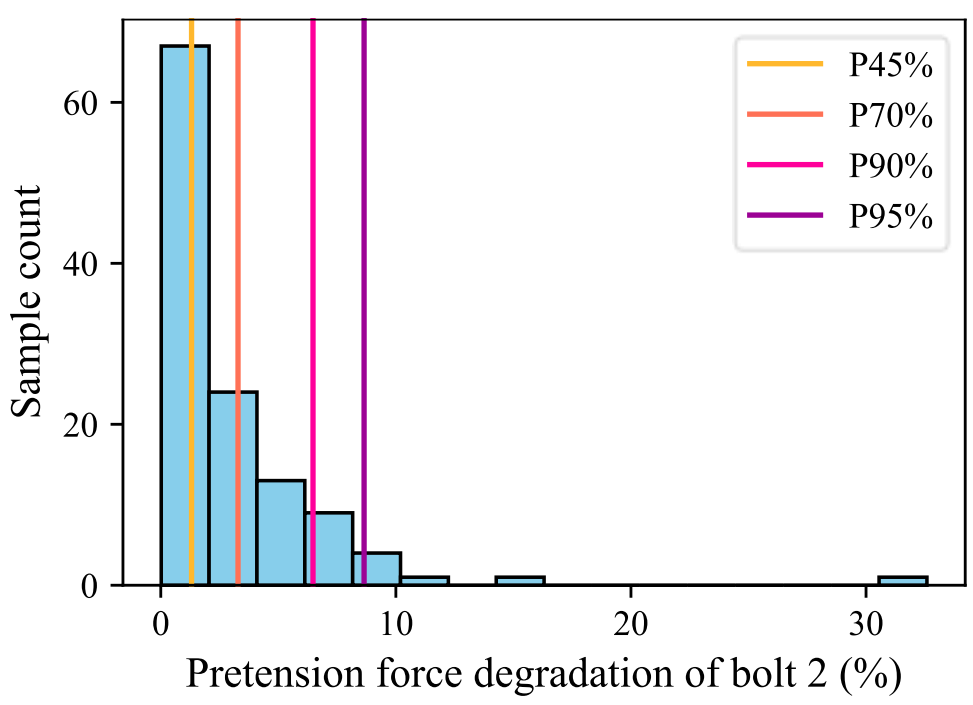

(c)

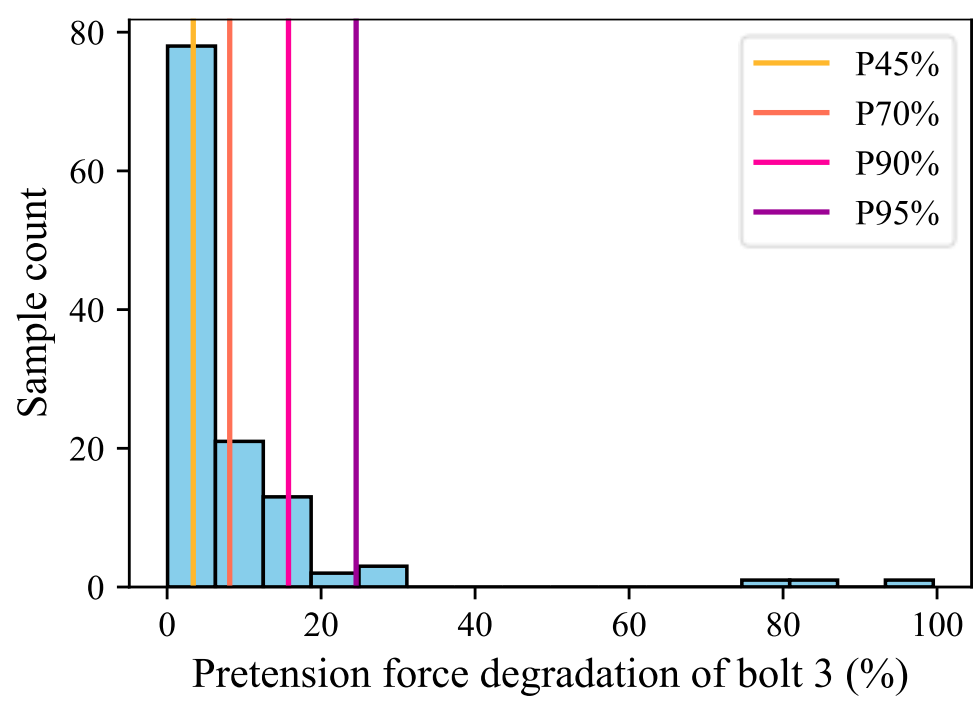

(d)

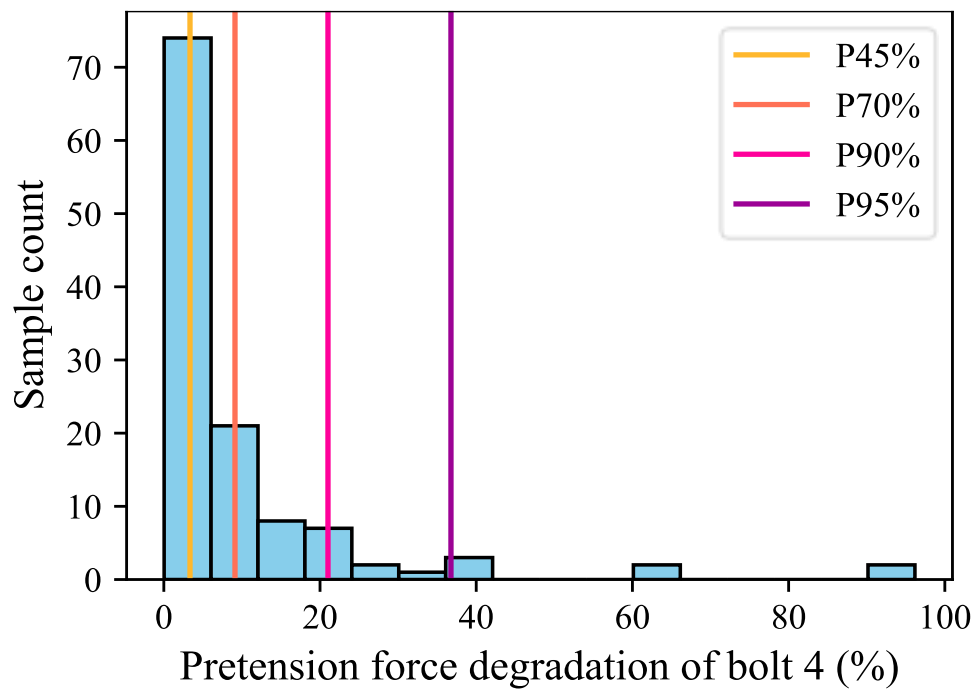

(e)

Fig. S10. Imbalanced distributions of stress and pretension-degradation responses. (a) Critical stress distribution; (b–e) Pretension degradation distributions of bolts 1–4. Colored vertical lines indicate the 45th, 70th, 90th, and 95th percentiles of each response distribution.

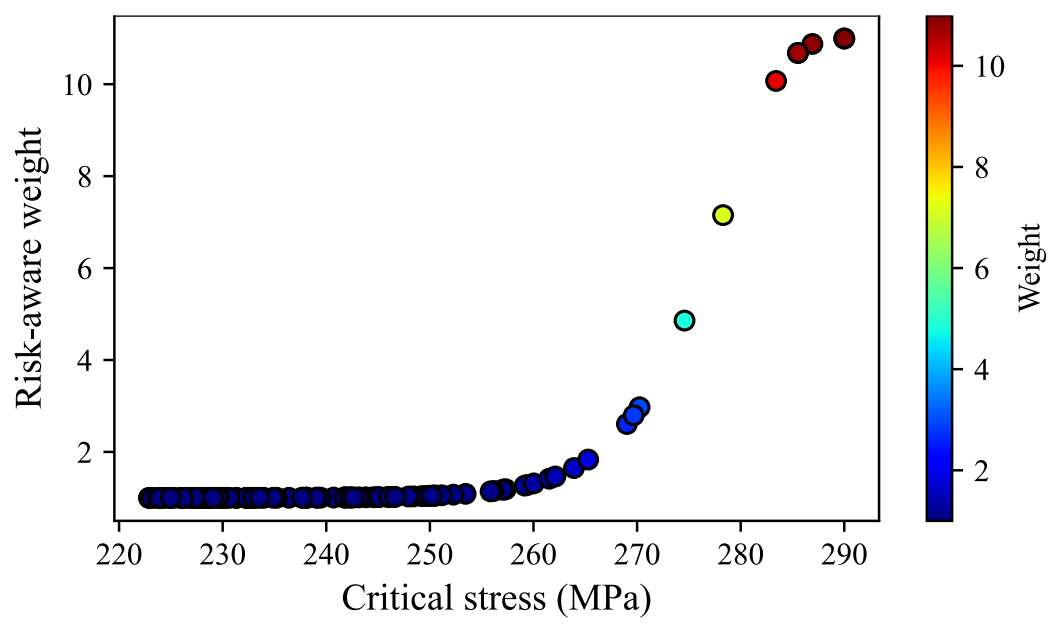

(a)

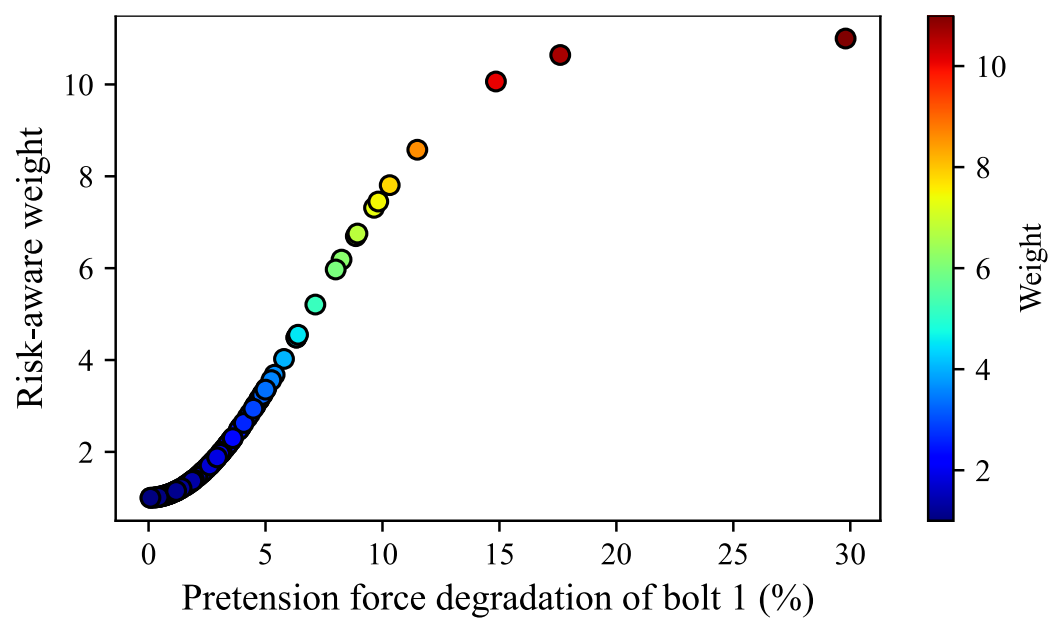

(b)

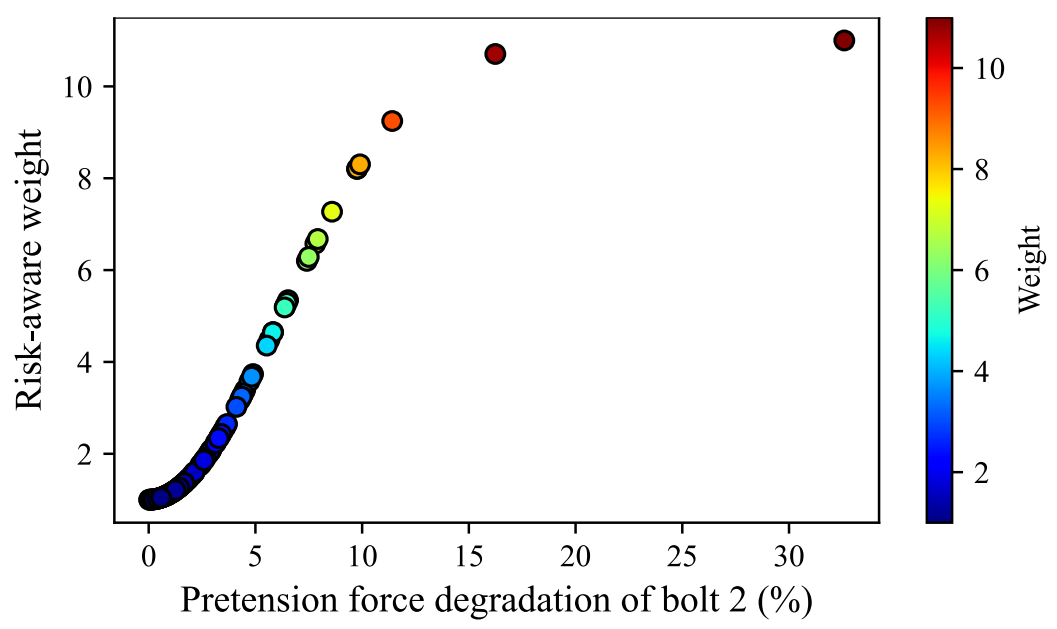

(c)

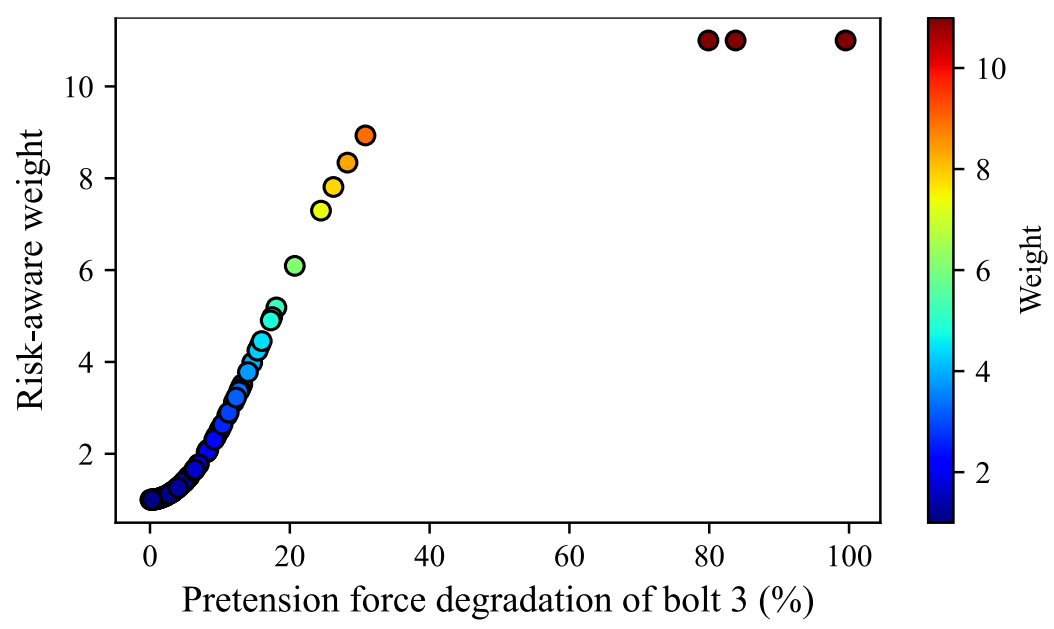

(d)

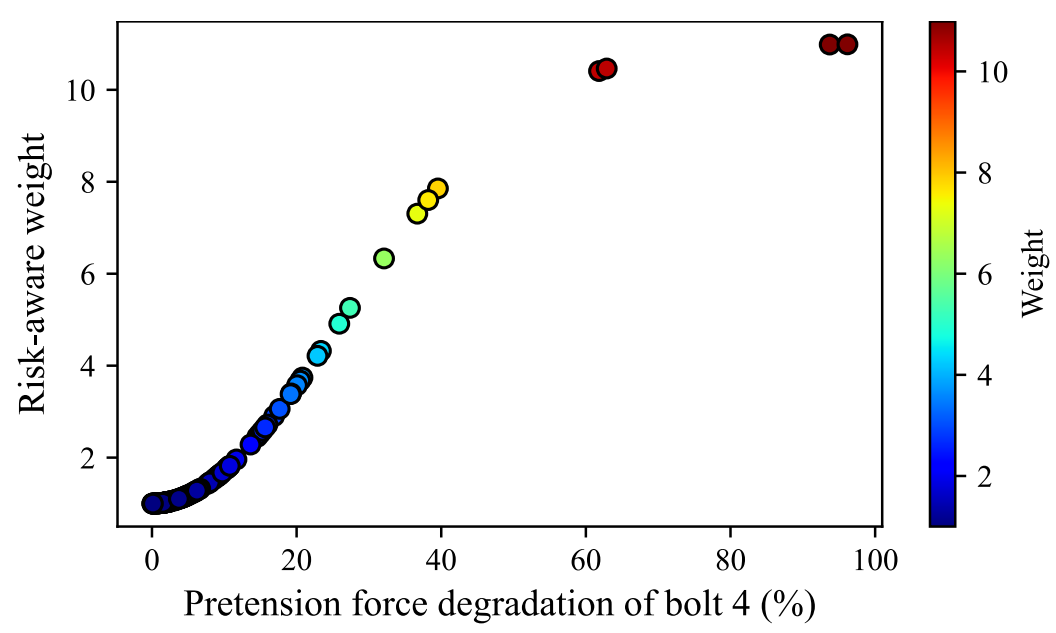

(e)

Fig. S11. Response-dependent training weights derived from the Weibull risk-weighting function. (a) Critical stress; (b–e) Pretension degradation of bolts 1–4. Color mapping represents the assigned

sample weights used in risk-aware model training.

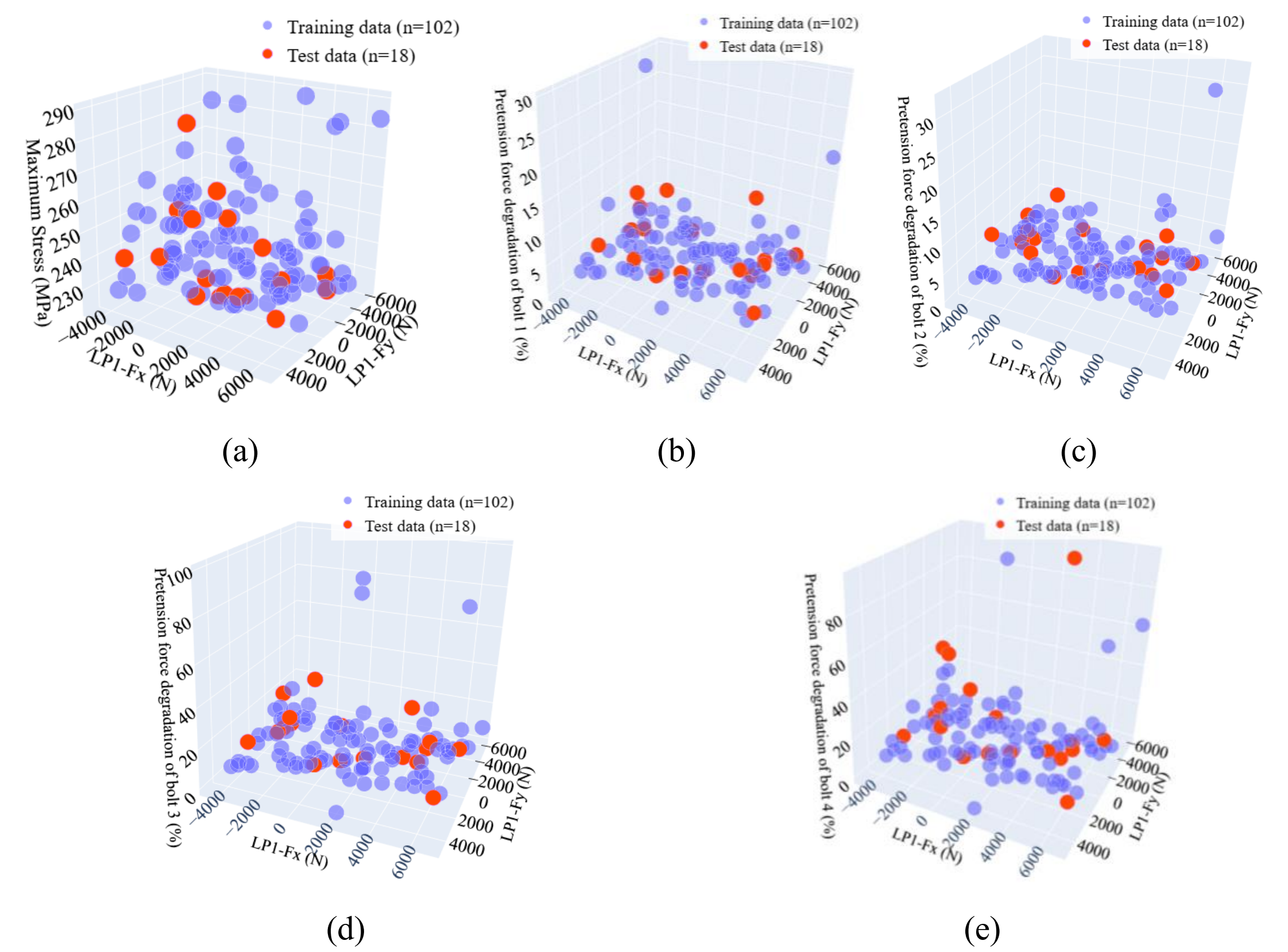

Fig. S12 Training and testing distributions across prediction targets. Spatial distribution of training and testing samples for (a) critical stress and (b–e) pretension degradation of bolts 1–4. The training set contains 85% of samples, and the testing set contains the remaining 15%.

Supplementary Tables.

Table S1

Evaluation metrics of predictive models (testing set).

Model name	RMSE	MSE	MAE	MAPE	RAE	R^2	RMO
MLR	9.96	99.23	8.11	3.39%	0.80	0.37	1.27
RF	8.48	71.88	6.69	6.09%	21.11	0.68	0.32
XGBoost	5.22	27.26	4.01	6.01%	23.54	0.86	1.37
BP-NN	4.49	20.18	3.29	1.39%	0.30	0.91	2.25
FA-RA MLR	3.22	10.35	2.50	1.06%	0.19	0.96	1.02
FA-RA RF	5.84	34.14	5.11	4.51%	19.83	0.74	0.24
FA-RA XGBoost	5.53	30.54	4.54	6.95%	21.31	0.89	1.20
FA-RA BP-NN	5.79	33.57	4.61	1.91%	0.42	0.84	0.13
RA BP-NN	5.03	25.29	4.19	1.76%	0.38	0.88	1.57

Table S2

Contact interaction types and friction coefficients for FE simulations.

Interaction pairs		Type	Coefficient
Component A	Component B		
Knuckle	Bearing	Coulomb friction	0.10(lubricated)
Knuckle	Washers	Coulomb friction	0.30(dry)
Knuckle	Bolts	Coulomb friction	0.25(dry)
Bolts	Washers	Coulomb friction	0.40(dry)
Bearing	Bolts	Tie	\

Table S3

Summary of critical stress demand and pretension force degradation across 20 load cases.

Results / Cases	Peak stress of knuckle (MPa)	Pretension force degradation (%)			
		Bolt1	Bolt2	Bolt3	Bolt4
Case 1	233.57	3.87	4.44	3.17	4.1
Case 2	241.72	3.13	1.14	9.42	20.06
Case 3	246.55	1.41	1.38	14.62	16.9
Case 4	225.86	0.65	0.71	1.51	1.08
Case 5	228.67	0.94	0.77	2.18	1.83
Case 6	223.92	0.5	0.15	0.44	0.27
Case 7	224.79	0.59	0.22	0.55	0.38
Case 8	229.97	2.35	2.4	1.25	1.9
Case 9	226.82	1.36	0.86	0.63	0.95
Case 10	229.63	1.95	1.03	0.86	1.15
Case 11	223.71	0.19	0.09	0.11	0.07
Case 12	224.03	0.27	0.12	0.15	0.09
Case 13	223.13	0.48	0.44	0.66	0.32
Case 14	223.9	0.09	0.02	0.06	0.04
Case 15	223.09	0.44	0.14	0.22	0.17
Case 16	224.29	0.54	0.51	0.31	0.68
Case 17	224.16	0.08	0.16	0.23	0.39
Case 18	223.97	0.32	0.22	0.11	0.13
Case 19	244.58	7.13	0.75	2.11	11.68
Case 20	248.42	9.64	4.11	4.21	19.24

Table S4

Configuration of predictive model variants and ablation schemes.

Scheme	Feature augmentation	Risk-aware learning	Models	Purpose

S_1	×	×	MLR, RF, XGBoost, BP-NN	Raw-feature baseline
S_2	✓	✓	MLR, RF, XGBoost, BP-NN	Full FA/RA configuration
S_3	×	✓	BP-NN	RA-only ablation for BP-NN

Table S5

Mathematical formulation of each evaluation metric.

Evaluation metrics	Expression
RMSE (Root Mean Squared Error)	$RMSE = \sqrt{\frac{1}{N}\sum_{i=1}^{N}(y_i - \hat{y}_i)^2}$
MSE (Mean Squared Error)	$MSE = \frac{1}{N}\sum_{i=1}^{N}(y_i - \hat{y}_i)^2$
MAE (Mean Absolute Error)	$MAE = \frac{1}{N}\sum_{i=1}^{N}\lvert y_i - \hat{y}_i\rvert$
MAPE (Mean Absolute Percentage Error)	$MAPE = 100\% \times \frac{1}{N}\sum_{i=1}^{N}\frac{\lvert y_i - \hat{y}_i\rvert}{\lvert y_i\rvert}$
RAE (Relative Absolute Error)	$RAE = \frac{\sum_{i=1}^{N}\lvert y_i - \hat{y}_i\rvert}{\sum_{i=1}^{N}\lvert y_i - \bar{y}\rvert}$
R^2 (Coefficient of Determination)	$R^2 = 1 - \frac{\sum_{i=1}^{N}(y_i - \hat{y}_i)^2}{\sum_{i=1}^{N}(y_i - \bar{y})^2}$
RMO (Residual mean offset)	$RMO = \lvert u_r\rvert, u_r = \text{argmax}\,[p(r)]$ $p(r) = \frac{1}{\sigma\sqrt{2\pi}} exp\left(-\frac{(r-u_r)^2}{2\sigma^2}\right)$

Here, y_i and $\hat{y}_i$ denote the FEA and predicted responses, respectively; $\bar{y}$ is the mean FEA response; N is the number of samples; and $r = y_i - \hat{y}_i$ is the residual.

Supplementary Methods

Supplementary Method 1. Finite-element model details

Supplementary Method 1.1. Simulation platform and constitutive material models

All finite-element simulations were implemented using ABAQUS and HyperWorks. The steering knuckle was modelled using A356-T6 aluminium alloy, with material information based on reported alloy data **[1-3]**. SCM435 steel was used for the bearing bolts, while C56E2 and 65Mn steels were assigned to the bearing and washer components. Mechanical properties were obtained from standards and peer-reviewed databases **[4-7]** and implemented as elastoplastic constitutive models.

The constitutive stress–strain relationships and mechanical properties used in the simulations are shown in Fig. S7.

Supplementary Method 1.2. Mesh discretization

A two-step meshing strategy was used to represent the complex knuckle–bolt geometry. A surface mesh was first generated to capture bolt holes, fillets, and contact interfaces, followed by volume meshing with second-order solid elements. The average and minimum element sizes were 2.8 mm and 0.7 mm, respectively, with aspect ratios below 4.0. Second-order solid elements were used to improve stress resolution in contact and transition regions **[8].**

The knuckle and bearing were meshed using C3D10 tetrahedral elements, whereas bolts and washers were meshed using C3D20 hexahedral elements. Local refinement was applied around bolt sections and stress-concentration regions. The final finite-element model contained 579,179 C3D10 elements, 23,808 C3D20 elements, and 968,468 nodes. The finite-element mesh is shown in Fig. S8.

Supplementary Method 1.3. Contact interactions and simulation settings

Contact interactions were defined using Coulomb friction models, with coefficients selected from standard recommendations **[9]**. Lubricated contact was applied to the knuckle–bearing pair, while dry friction was used for the remaining frictional interfaces. The bearing–bolt interface was simplified using tie constraints to approximate the threaded connection. The contact interaction settings are summarized in Table S2.

Supplementary Method 1.4 Baseline finite-element responses for the twenty load cases

The twenty multibody-derived baseline load cases were simulated using identical preload, boundary, contact, and unloading conditions. Each case applied six-component force–moment vectors at LP1–LP4, with an initial bolt pretension of 60 kN. The extracted outputs included the peak von Mises stress of the knuckle and pretension degradation of the four bolts.

The full baseline response data are provided in Table S3. These results show that peak stress and bolt pretension degradation depend strongly on load-path characteristics. Peak knuckle stress ranges from 223.09 MPa to 248.42 MPa, while pretension degradation varies substantially across bolts and loading cases, confirming the nonlinear coupling between global multiaxial loading and localized contact-driven degradation.

Supplementary Method 2. Statistical expansion and dataset composition

The original twenty multibody-derived load cases were obtained from a finite set of representative vehicle events. Although these cases capture important adverse service

conditions, they do not exhaust the full operational uncertainty of a real vehicle system. During service, the structure may experience additional load variations caused by impact events, wind disturbance, road roughness, potholes, bumps, and other stochastic operating conditions. If the learning model is trained only on the limited MBD-derived cases, it may overfit to the simulated event set and show reduced robustness when exposed to unseen load combinations.

To mitigate this issue, randomized uncertainty sampling was used to broaden the finite-element response dataset. The sampling was not performed over an arbitrary load domain. Instead, multibody simulations were first used to identify an adverse yet physically meaningful load envelope. The force and moment components in this envelope were treated as signed Cartesian vector components rather than unsigned load magnitudes. Therefore, positive and negative values represent opposite directions along the corresponding coordinate axis. For example, forward and backward driving events, or left- and right-turning events, may produce opposite signs for the same load component at a given load-transfer point. If the original MBD-derived cases contain both negative and positive values for a component such as F_x, the statistically generated value $\tilde{F}_x$ is sampled within this signed directional interval. A negative sampled value therefore represents loading along the negative x-direction, whereas a positive value represents loading along the positive x-direction. New load vectors were then sampled within expanded bounds derived from the minimum and maximum values of the original twenty cases, using an expansion factor of $\alpha^l = 1.1$. This strategy preserves a connection to physically plausible operating conditions while exposing the learning models to broader combinations of force–moment interactions.

Each additional load vector was evaluated using the same finite-element protocol as the baseline cases. Therefore, the expanded dataset did not rely on interpolated response labels. Every sample had FE-simulated response targets, including the peak von Mises stress of the knuckle and pretension degradation values of the four bolts.

The final dataset consisted of 120 FE-simulated samples. Each sample contained:

(i) A 24-dimensional multiaxial load input vector;

(ii) The corresponding peak von Mises stress of the knuckle;

(iii) Pretension degradation values for bolts 1–4.

This expanded dataset was used for predictive model training, testing, benchmarking, and DRS-based structural risk assessment.

Supplementary Method 3. Mechanics-informed feature augmentation

Supplementary Method 3.1 Raw-feature correlation under multiaxial loading

The original input matrix contained 24 load components for each sample, corresponding to six force–moment components at four load-transfer points. Pearson correlation analysis was used to examine the pairwise linear relationships among these raw load components. As shown in Fig. S9(a), most correlations were weak, indicating that raw load components alone cannot fully represent the nonlinear mechanics of the jointed structure. For example, the correlations between LP2-F_x and LP3-F_z, and between LP3-F_y and LP1-M_x, were both 0.03. Even the strongest observed correlation, between LP1-F_x and LP4-F_x, was only 0.29.

These weak pairwise correlations do not imply that the load components are mechanically irrelevant. Instead, they indicate that the important relationships are not well captured by simple linear correlation. Under multiaxial loading, stress redistribution and bolt pretension

degradation are often controlled by nonlinear combinations of forces and moments. This is why polynomial and harmonic feature augmentation was introduced.

Supplementary Method 3.2 Polynomial interaction features

Polynomial feature augmentation explicitly constructs nonlinear combinations of the original load components. For an input vector:

$$X = (x_1, x_2, x_3, \dots, x_d) \tag{1}$$

the second order ($p = 2$ proposed in the main text) polynomial expansion includes squared terms and pairwise products:

$$x_1^2, x_1x_2, x_1x_3, \dots \dots, x_ax_b \tag{2}$$

where $1 \leq a \leq b \leq d$. For $d = 24$, this gives:

$$C_{24+2-1}^2 = \frac{25!}{2! \times (25-2)!} = 300 \tag{3}$$

second order polynomial features.

A simple three-feature example helps illustrate the idea. If the original input is

$$(x_1, x_2, x_3) \tag{4}$$

Then the interaction-type expanded features can include:

$$x_1, x_2, x_3, x_1x_2, x_1x_3, x_2x_3 \tag{5}$$

and, when squared terms are included,

$$x_1^2, x_2^2, x_3^2 \tag{6}$$

These terms allow a model to represent situations where the structural response depends not only on a single load component, but on the combined effect of two load components. For example, stress concentration may become large only when a lateral force and a bending moment act together.

Supplementary Method 3.3 Harmonic features

Harmonic features were introduced to represent sign-dependent and possible oscillatory effects in the load–response relationship. For each raw load component x_i, sine and cosine terms were generated as:

$$\sin\left(\frac{x_i}{K}\right), \cos\left(\frac{x_i}{K}\right) \tag{7}$$

where K is a scaling factor. In the present implementation, one sine and one cosine term were generated for each of the 24 raw load components, producing 24 sine features and 24 cosine features.

Using the same simple three-feature example, the augmented representation can be written conceptually as:

$$x_1, x_2, x_3, x_1x_2, x_1x_3, x_2x_3, \sin x_1, \sin x_2, \sin x_3, \cos x_1, \cos x_2, \cos x_3 \tag{8}$$

If squared terms are also included, the representation further contains:

$$x_1^2, x_2^2, x_3^2 \tag{9}$$

This example shows the purpose of feature augmentation: the model is no longer forced to learn all nonlinear relationships only from the raw variables. Instead, physically meaningful nonlinear and sign-dependent terms are provided directly as candidate predictors.

Supplementary Method 3.4 Final augmented feature space

In the present study, the final augmented feature vector consisted of 372 features, including 24raw force–moment features, 300 second-order polynomial features, 24 sine features and 24

cosine features.

The complete feature-space expansion from 24 raw load components to 372 augmented features is shown in Fig. S9(b). This augmented feature matrix was used as the input for the feature-augmented learning models.

Supplementary Method 4. Response imbalance and Weibull-based risk weighting

Supplementary Method 4.1 Response imbalance after load-space expansion

Although the load vectors were expanded through randomized uniform sampling in the admissible multiaxial load space, the resulting structural responses remained strongly imbalanced. This occurs because the mapping from load inputs to structural responses is nonlinear: different force–moment combinations may produce limited stress changes, whereas a small subset can trigger strong stress concentration or substantial bolt pretension loss.

Fig. S10 shows the response distributions of critical stress and bolt pretension degradation. Critical-stress samples above 280 MPa account for less than 5% of the dataset. The imbalance is more evident for bolt pretension degradation, where most samples show small pretension loss and only a few cases reach severe degradation. These sparse upper-tail samples are important for reliability-oriented prediction but can be under-emphasized when all samples are assigned equal training importance.

Supplementary Method 4.2 Risk-weight distributions

The Weibull-based weighting function assigns larger weights to samples with more severe responses. Fig. S11 shows the resulting sample weights for critical stress and pretension degradation of bolts 1–4. For critical stress, weights increase near the upper tail of the response distribution, with high-weight samples mainly appearing above approximately 270 MPa. For bolt pretension degradation, higher weights are assigned to samples with substantial pretension loss, typically above approximately 10–20%, depending on the bolt.

These weight distributions confirm the intended behavior of the risk-aware objective: low-severity samples remain close to unit weight, whereas sparse high-severity samples contribute more strongly during training. The weighting is continuous rather than threshold-based, so it avoids defining a binary failure boundary during model fitting.

Supplementary Method 5. Predictive model configurations and evaluation metrics

Supplementary Method 5.1 Baseline predictive models

Four regression models were used to benchmark the proposed feature augmentation and risk-aware learning strategies: multiple linear regression (MLR), random forest (RF), extreme gradient boosting (XGBoost), and backpropagation neural network (BP-NN). These models span interpretable linear regression, ensemble tree learning, gradient boosting, and neural-network-based nonlinear mapping.

(1) MLR

MLR assumes a linear relationship between the input feature vector $X_i \in \mathbb{R}^d$ and the predicted response $\hat{y}_i$:

$$\hat{y}_i = X_i^{\mathrm{T}} \theta \tag{10}$$

where $\theta \in \mathbb{R}^d$ denotes the regression coefficient vector. The model minimizes the mean

squared error (MSE) loss:

$$\ell(\theta) = \frac{1}{N}\sum_{i=1}^{N}(y_i - \hat{y}_i)^2 \tag{11}$$

and updates parameter iteratively through gradient descent:

$$\theta^{(t+1)} = \theta^{(t)} - \eta \, \nabla_\theta \, \ell(\theta) \tag{12}$$

where η is the learning rate. When applied to the augmented feature space, MLR retains interpretability while gaining the ability to approximate nonlinear load–response relationships through polynomial and harmonic input terms [10].

(2) RF

RF constructs an ensemble of regression trees using bootstrap sampling and randomized feature selection [11]. At each node, the split is selected to maximize the reduction in prediction impurity:

$$\Delta I = I(D) - \sum \frac{|D_j|}{|D|} I(D_j) \tag{13}$$

where D is the parent node, D_j denotes a child node, and $I(\cdot)$ represents a regression impurity measure such as variance or mean squared error. For regression tasks, the final prediction is obtained by averaging the outputs of T trees:

$$\hat{y}_i = \frac{1}{t}\sum_{i=1}^{t} f_i\,(x) \tag{14}$$

where $f_i(x)$ is the output of the i-th tree for input x.

This ensemble structure reduces variance and provides a nonlinear benchmark for evaluating the proposed FA/RA strategies.

(3) XGBoost

XGBoost builds an additive ensemble of regression trees by sequentially fitting residual errors under a regularized objective [12]. At iteration t, the objective is written as:

$$\mathcal{L}^{(t)} = \sum_{i=1}^{N} \ell\left(y_i, \hat{y}_i^{(t)}\right) + \sum_{i=1}^{t} \Omega(f_i) \tag{15}$$

where $\Omega(f_i)$ penalizes the complexity of tree f_m. Using a second-order Taylor approximation, the objective for the newly added tree f_t becomes:

$$\mathcal{L}^{(t)} \approx \sum_{i=1}^{N} \left[g_i f_t(x_i) + \frac{1}{2} h_i f_t^2(x_i)\right] + \Omega(f_t) \tag{16}$$

where g_i and h_i are the first- and second- order derivatives of the loss with respect to the current prediction. The split gain is expressed as:

$$Gain_{XGBoost} = \frac{1}{2}\left[\frac{G_L^2}{H_L+\lambda} + \frac{G_R^2}{H_R+\lambda} - \frac{(G_L+G_R)^2}{H_L+H_R+\lambda}\right] - \gamma \tag{17}$$

where and G_R denote the cumulative first-order gradients over the left and right child nodes, respectively; H_L and H_R denote the corresponding cumulative second-order gradients; and λ and γ are regularization parameters. XGBoost provides a strong nonlinear ensemble benchmark for the proposed framework.

(4) BP-NN

BP-NN represents nonlinear mappings through multiple fully connected layers [13]. The output of an L-layer network can be written as:

$$\hat{y}_i = f^{(L)}(W^{(L)} f^{(L-1)}(\cdots f^{(1)}(W^{(1)} x_i + b^{(1)}) \cdots) + b^{(L)}) \tag{18}$$

where $W^{(l)}$, $b^{(l)}$ and $f^{(l)}(\cdot)$ denote the weight matrix, bias vector, and activation function

of layer l, respectively. Parameters are updated through backpropagation:

$$\frac{\partial \mathcal{L}}{\partial \theta} = \frac{\partial \mathcal{L}}{\partial \hat{y}_i} \cdot \frac{\partial \hat{y}_i}{\partial f^{(l)}} \cdot \frac{\partial f^{(l)}}{\partial \theta} \tag{19}$$

where θ denotes all trainable network parameters. BP-NN provides a flexible nonlinear benchmark for evaluating whether FA/RA improves learning stability and high-risk sensitivity beyond the intrinsic expressiveness of neural networks.

Supplementary Method 5.2 FA/RA integration schemes

To evaluate how input representation and response-severity weighting affect predictive performance, three modelling schemes were defined. Scheme S1 used raw load features and uniform sample weights for all four baseline models. Scheme S2 applied both feature augmentation and risk-aware learning to the same four models, representing the full FA/RA configuration. Scheme S3 applied risk-aware learning only to BP-NN, serving as an ablation case for assessing the role of feature augmentation in neural-network learning.

The full configuration table is provided in Table S4. This design enables controlled comparison of the contributions of feature augmentation and risk-aware weighting across linear regression, ensemble learners, gradient boosting, and neural networks.

Supplementary Method 5.3 Training–testing split and overfitting considerations

The 120 FE-simulated samples were divided into training and testing subsets for each prediction task, with 85% of the samples assigned to training and the remaining 15% assigned to testing. The resulting distributions across critical stress and bolt pretension-degradation targets are shown in Fig. S12. Both subsets cover the main response range and include upper-tail samples, allowing model performance to be evaluated not only in common response regimes but also near structurally relevant high-response regions.

Because feature augmentation increased the input dimension from 24 raw features to 372 augmented features, possible overfitting was considered during model development and evaluation. At the data level, statistical load-space expansion was used to reduce dependence on a small set of typical MBD-derived service events. The additional samples were generated by randomized sampling within an MBD-derived physically admissible load envelope and were then evaluated by finite-element simulation, rather than by interpolating response labels. This procedure exposes the models to broader force–moment combinations while preserving mechanically simulated response targets.

At the model level, overfitting can be mitigated by controlling model complexity and adding penalty terms to the learning objective. In this study, RF reduces variance through ensemble averaging, XGBoost uses regularization terms to penalize overly complex trees, and neural-network training can incorporate regularization to restrict excessive parameter growth. At the evaluation level, model performance was assessed primarily on the testing set using seven metrics and residual diagnostics, rather than relying only on training performance or R^2. Nevertheless, external component-level experiments and field-monitoring data are still required to further assess generalization under real operating conditions.

Supplementary Method 5.4 Evaluation metrics

Seven metrics were used to evaluate predictive performance: RMSE, MSE, MAE, MAPE, RAE, R^2, and RMO. These metrics quantify complementary aspects of model behaviour, including absolute error magnitude, relative error, goodness of fit, and residual bias. The mathematical definitions are provided in Table S5.

Supplementary Notes

Supplementary Note 1. Detailed prediction–observation and residual diagnostics

Supplementary Note 1.1 Critical-stress prediction and residual diagnostics

The prediction–observation and residual analyses provide additional support for the benchmarking trends reported in the main text. Fig. S1 compares predicted and physical critical stress, testing residuals, and residual distributions for all evaluated models.

Baseline MLR shows clear limitations when applied directly to raw multiaxial load inputs. It systematically underestimates critical stress in several high-stress testing samples, indicating that a raw linear mapping is insufficient for representing the nonlinear force–moment coupling and load-transfer behavior of the bolted steering-knuckle assembly. After introducing polynomial–harmonic feature augmentation and risk-aware learning, FA-RA MLR aligns more closely with the perfect-fit line and keeps most testing predictions within the ±5% relative error band. This confirms that the augmented feature space substantially improves the ability of a transparent linear model to capture nonlinear stress responses.

Tree-based models show a different trend. RF and XGBoost already provide nonlinear mappings and therefore achieve stronger baseline performance than MLR. After FA/RA integration, their prediction alignment improves further, although the gains are more moderate than those observed for MLR. This suggests that FA/RA contributes not only by increasing model flexibility but also by improving the representation of mechanically meaningful load interactions and upper-tail response patterns.

Neural-network models also capture the main stress trend at baseline, but the FA/RA and RA configurations improve residual symmetry and reduce systematic offset. In particular, the improved residual centering indicates that risk-aware learning helps stabilize predictions in high-response regions without relying solely on overall error minimization.

Overall, the critical-stress residual diagnostics show that FA/RA integration improves more than average predictive accuracy. It also enhances prediction stability in upper-tail stress regimes, where underestimation or biased residuals would directly affect subsequent degradation risk scoring and reliability assessment.

Supplementary Note 1.2 Bolt pretension-degradation prediction

The bolt pretension-degradation task evaluates whether the proposed learning strategy can also capture connection-level degradation, not only critical stress response. Fig. S2 presents predicted versus physical pretension degradation for bolts 1–4, together with ±5%, ±15%, and ±30% relative error bands.

Under the joint risk-aware objective, FA-RA MLR achieves an overall $R^2 = 0.90$for bolt pretension degradation. Most testing samples fall within the ±15% relative error band, indicating stable prediction accuracy across multiple bolt-level outputs. This result is important because bolt pretension degradation is more unevenly distributed than critical stress, with severe degradation appearing only in a small number of cases.

For bolts 3 and 4, where large pretension loss occurs in sparse upper-tail samples, the predictions remain close to the perfect-fit line over a broader degradation range. This suggests that risk-aware weighting improves model sensitivity to severe degradation cases that would otherwise be underrepresented under uniform loss functions. The bolt-level results are therefore consistent with the critical-stress results: mechanics-informed feature augmentation improves

nonlinear representation, while risk-aware learning preserves sensitivity to structurally important upper-tail responses.

Together, the stress and pretension-degradation predictions provide the two response inputs required for the DRS-based risk assessment in the main text.

Supplementary Note 2. Bayesian updating formulation for calibrating failure probability from predicted stress and pretension degradation

This supplementary note details two Bayesian calibration formulations for linking the predicted stress response and bolt pretension degradation to failure probability. A logistic model is first introduced to explicitly relate D_{res}, D_{pre}, and their interaction to the probability of failure. A Weibull-based model is then presented to calibrate the integrated Degradation Risk Score (DRS) as a scalar vulnerability indicator. Together, these formulations provide probabilistic extensions of the proposed FA/RA–DRS framework when inspection, testing, or monitoring data are available.

Supplementary Note 2.1 Bayesian logistic failure-probability model

(1) Logistic formulation

In the first formulation, the probability of failure for the i-th loading case is directly related to the stress-related degradation component and the pretension-degradation component:

$$P_f = P\left(failure \middle| D_{res,i}, D_{pre,i}, \theta\right) \tag{20}$$

where

$$\theta = (\theta_0, \theta_1, \theta_2, \theta_3) \tag{21}$$

is a vector of unknown parameters. A logistic failure-probability model can be written as:

$$P_f = \frac{1}{1+\ e^{-\eta_i}} \tag{22}$$

with

$$\eta = \theta_0 + \theta_1 D_{res,i} + \theta_2 D_{pre,i} + \theta_3 D_{res,i} D_{pre,i} \tag{23}$$

Where, θ_0 represents the baseline tendency toward failure, θ_1 controls the contribution of material overstress, θ_2 controls the contribution of bolt pretension degradation, and θ_3 captures the coupled effect between material overstress and connection degradation.

(2) Bayesian updating of logistic parameters

An initial prior distribution is assigned to the unknown logistic parameter vector $p(\theta)$. The prior can be specified using engineering experience, previous experiments, historical reliability data, or conservative expert judgment.

Assume that new inspection, testing, simulation, or monitoring data become available:

$$\mathbb{D} = \left\{D_{res,i}, D_{pre,i}, Y_i\right\}_{i=1}^{n} \tag{24}$$

where Y_i is a binary indicator of structural state:

$$Y_i = \begin{cases} 1, & failure\ or\ high\,risk\ state \\ 0, & safe\ state \end{cases} \tag{25}$$

The posterior distribution of θ is obtained using Bayes' theorem:

$$p(\theta|\mathbb{D}) = \frac{p(\mathbb{D}|\theta)p(\theta)}{p(\mathbb{D})} \tag{26}$$

For binary state observations, the likelihood function is

$$p(\mathbb{D}|\theta) = \prod_{i=1}^{n} P_{f,i}^{Y_i}(1 - P_{f,i})^{1-Y_i} \quad (27)$$

This likelihood assigns higher probability to parameter values that predict large $P_{f,i}$ for observed failure or high-risk samples ($Y_i = 1$), and small $P_{f,i}$ for observed safe samples ($Y_i = 0$). Therefore, Bayesian updating refines the relationship between the degradation indicators and the failure probability as additional observations accumulate.

(3) Prediction for a new loading case

For a new multiaxial loading case, the FA/RA model first predicts critical stress and bolt pretension degradation:

$$X_* \to \hat{\sigma}_{crit,*}, \hat{D}_{pre,*} \quad (28)$$

These predictions are then converted into $D_{res,*}$ and $D_{pre,*}$. The calibrated failure probability is obtained by integrating over the posterior uncertainty of θ:

$$P_{f,*} = \int P\left(failure \middle| D_{res,i}, D_{pre,i}, \theta\right) p(\mathbb{D}|\theta) d\theta \quad (29)$$

This marginalization accounts for uncertainty in the logistic parameters rather than relying on a single deterministic estimate.

Supplementary Note 2.2 Bayesian Weibull risk-calibration model

(1) Weibull formulation based on DRS

In the second formulation, the DRS is used directly as a scalar vulnerability indicator, and the probability of failure is modeled using a Weibull-type risk function:

$$P_f = P(failure|DRS_i, \lambda, k) \quad (30)$$

where $\lambda > 0$is the scale parameter and $k > 0$is the shape parameter. The Weibull risk-calibration function is written as:

$$P_{f,i} = 1 - e^{-(\frac{DRS_i}{\lambda})^k} \quad (31)$$

where $\theta = (\lambda, k)$ is the unknown parameter vector.

In this formulation, λcontrols the risk scale, while k controls how rapidly the failure probability increases with DRS. A smaller λ or a larger k generally leads to a sharper increase in failure probability as DRS_iapproaches high-risk values.

(2) Bayesian updating of Weibull parameters

A prior distribution is assigned to the Weibull parameters:

$$p(\lambda, k) \quad (32)$$

When observed safe or failure states are available, the dataset can be written as:

$$\mathbb{D} = \{DRS_i, Y_i\}_{i=1}^{n} \quad (33)$$

where

$$Y_i = \begin{cases} 1, & failure\ or\ high\ risk\ state \\ 0, & safe\ state \end{cases} \quad (34)$$

The posterior distribution is obtained as:

$$p(\lambda, k|\mathbb{D}) = \frac{p(\mathbb{D}|\lambda,k)p(\lambda,k)}{p(\mathbb{D})} \quad (35)$$

The likelihood function is

$$p(\mathbb{D}|\lambda, k) = \prod_{i=1}^{n} P_{f,i}^{Y_i}(1 - P_{f,i})^{1-Y_i} \quad (36)$$

This likelihood increases when the Weibull function assigns high failure probability to observed failure or high-risk samples and low failure probability to safe samples.

(3) Prediction for a new loading case

For a new multiaxial loading case, the FA/RA–DRS framework first computes:

$$DRS_* = 1 - (1 - D_{res,*})(1 - D_{pre,*}) \tag{37}$$

The calibrated failure probability is then obtained by marginalizing over the posterior uncertainty in $\lambda$and k:

$$P_{f,*} = \int \int P(failure|DRS_*, \lambda, k)\, p(\mathbb{D}|\lambda, k) d\lambda dk \tag{38}$$

Supplementary Note 2.3 Comparison of the two Bayesian calibration forms

The two Bayesian formulations serve similar purposes but differ in how failure probability is linked to the predicted degradation indicators.

(1) Bayesian-Logistic form

The logistic model uses $D_{res}$and D_{pre}as separate inputs:

$$P_f = P(failure|D_{res,i}, D_{pre,i}, \theta) \tag{39}$$

It is suitable when the individual contributions of material overstress and connection degradation need to be explicitly distinguished. The interaction term $D_{res,i}D_{pre,i}$ also allows the model to capture the coupled effect between the two degradation pathways.

(2) Bayesian-Weibull form

The Weibull model uses the integrated DRS as a scalar input:

$$P_f = P(failure|DRS_i, \lambda, k) \tag{40}$$

It is more compact and directly aligns with the risk-score formulation used in the proposed framework. It is suitable when DRS is treated as the primary vulnerability indicator and the objective is to calibrate the relationship between DRS and failure probability.

Supplementary Note 2.4 Relation to the proposed FA/RA–DRS framework

The proposed FA/RA–DRS framework can be interpreted as the front-end mechanics-informed response prediction module:

$$X_i \to \hat{\sigma}_{crit,i}, \hat{D}_{pre,i} \tag{41}$$

The predicted responses are converted into degradation indicators:

$$\hat{\sigma}_{crit,i}, \hat{D}_{pre,i} \to D_{res,i}D_{pre,i} \tag{42}$$

and then integrated into a risk score:

$$D_{res,i}D_{pre,i} \to DRS_i \tag{43}$$

A Bayesian updating layer can then be appended as a probability-calibration module:

$$(D_{res,i}D_{pre,i}) \to P_{f,i} \tag{44}$$

or

$$DRS_i \to P_{f,i} \tag{45}$$

Thus, the proposed FA/RA–DRS framework and Bayesian updating are complementary. The FA/RA component provides mechanics-informed response prediction under complex multiaxial loading, while Bayesian updating can calibrate the relationship between predicted degradation indicators and actual failure probability when component-level inspection, testing, or monitoring data become available.

Supplementary Discussion

Supplementary Discussion 1. Feature attribution, risk-aware reasoning, and transferability analysis

Supplementary Discussion 1.1 Feature-level interpretation of mechanics-informed augmentation

SHAP analysis was used to interpret how the mechanics-informed augmented features contributed to model predictions. The beeswarm plots in Fig. S4 provide a global ranking of influential features, whereas the heatmaps in Fig. S5 show sample-wise feature contributions. Together, these analyses clarify why feature augmentation improves prediction accuracy and how the learned feature importance relates to the degradation risk patterns discussed in the main text.

For critical-stress prediction, the dominant predictors are not isolated raw load components but interaction terms involving load points. Features such as $\mathrm{LP2\text{-}F_x \times LP2\text{-}F_y}$, $\mathrm{LP2\text{-}F_x \times LP4\text{-}F_x}$, and $\mathrm{(LP2\text{-}F_x)^2}$rank among the most important contributors. This indicates that critical stress is governed by coupled multiaxial force interactions rather than by a single load component. Mechanically, this agrees with the finite-element stress distributions [Fig 2], where stress concentrations occur near the flange–arm transition and bolt-hole regions under specific load combinations.

For bolt pretension degradation, the important features vary across bolts, showing that each bolt responds differently to the applied load paths. Bolt 2 is strongly influenced by features involving $\mathrm{LP1\text{-}F_z}$and its interaction with $\mathrm{LP4\text{-}F_y}$, whereas bolts 3 and 4 show stronger sensitivity to interaction terms involving $\mathrm{LP2\text{-}F_x}$, $\mathrm{LP2\text{-}F_y}$, and $\mathrm{LP4\text{-}F_y}$. This bolt-specific attribution pattern is consistent with the localized nature of pretension loss, where global multiaxial loading produces different contact deformation and residual force loss depending on bolt position and neighboring load-transfer paths.

Harmonic features, such as $\sin\left(1/K \times LP4\text{-}F_z\right)$, are generally less dominant than polynomial interaction terms in the global rankings. However, they show noticeable SHAP contributions in specific samples, suggesting that they help capture sign-dependent or oscillatory response patterns that are not fully represented by polynomial terms alone. Their role is therefore sample-dependent rather than globally dominant.

Overall, the SHAP results support the physical relevance of the proposed feature augmentation strategy. The most influential predictors are dominated by mechanics-informed interaction terms rather than raw load magnitudes, indicating that the augmented feature space improves model expressiveness while preserving interpretability.

Supplementary Discussion 1.2 Risk-aware learning and degradation-integrated reasoning

Reliability-oriented prediction requires not only low average error but also sensitivity to rare upper-tail response states, where stress demand or bolt pretension loss becomes structurally significant. The proposed framework addresses this requirement through two connected mechanisms: response-dependent risk-aware learning and DRS-based vulnerability assessment.

Risk-aware learning compensates for imbalanced response distributions by assigning larger training weights to severe stress or pretension-degradation samples. In the present dataset, most samples correspond to low or moderate stress and pretension degradation, whereas only a

small subset occupies the upper tail. The Weibull-type weighting function increases gradually for moderate responses and rises more sharply near high-stress or high-degradation regions. This smooth weighting avoids hard binary thresholds while helping the model learn response patterns that would otherwise be underrepresented under uniform loss functions.

The DRS plays a different role. Unlike Weibull training weights, which act during model fitting, DRS is applied after prediction to rank loading cases according to compounded degradation severity. Low-DRS samples correspond to cases with low stress-margin degradation and limited bolt pretension loss, whereas high-DRS samples occur when D_{res}, D_{pre}, or both become large. This separation is important: the Weibull weighting scheme improves training sensitivity, whereas DRS provides a deterministic vulnerability score for post-prediction ranking.

Neither the Weibull training weight nor DRS is treated as a directly calibrated probability of failure in the main workflow. When binary state labels, inspection records, testing observations, or monitoring data become available, Bayesian updating can be introduced as a probability-calibration layer. The FA/RA model first predicts critical stress and bolt pretension degradation from multiaxial load descriptors; these responses are converted into D_{res}, D_{pre}, and DRS; and Bayesian logistic or Weibull calibration can then map these indicators to P_f. This layered structure preserves the interpretability of the FA/RA–DRS framework while allowing future extension toward probabilistic reliability assessment.

Supplementary Discussion 1.3 Model reliability and transferability under uncertainty

Residual analyses show that baseline learners tend to produce biased or inflated errors in high-stress or severe-loss regions, whereas FA/RA integration mitigates these effects. Particularly, FA-RA MLR shows narrowed residuals in upper-tail stress regimes, while FA/RA-enhanced XGBoost and BP-NN improve residual symmetry and reduce systematic offset. For bolt-level degradation, risk-aware weighting helps the model track substantial pretension loss more consistently.

Performance benchmarking also suggests that transparent models with physics-aligned features may be effective in moderate-sample regimes. In the present dataset, FA-RA MLR achieves balanced testing performance across multiple metrics, whereas more flexible models benefit mainly through improved residual behaviour. This result should be interpreted within the scope of the current dataset rather than as a universal model-selection rule. The relative advantage of transparent or flexible models will depend on sample size, noise level, load-space coverage, and the complexity of the target structure.

For transfer to other multiaxial jointed systems, the framework should be adapted by redefining system-specific load descriptors, feature bases, and degradation indicators. For example, in bolted frames, flange connections, or pinned links, the same modelling logic can be retained, but the relevant load-transfer points, interaction terms, stress-margin definitions, and connection-degradation measures should be selected according to the governing mechanics of the target structure.

In real-world monitoring environments, the framework can be implemented as a sequential reliability-assessment loop. Structural load data are collected from sensors, dynamically passed into the calibrated FA/RA model, and converted into predicted stress and connection degradation. These predictions are then integrated into DRS and, when calibration data are

available, mapped to component-level failure probability through Bayesian updating. This workflow allows high-risk operating states to be identified and communicated to operators or maintenance personnel, supporting inspection planning and preventive maintenance.

Supplementary References

[1] Park C, Kim S, Kwon Y, et al. Mechanical and corrosion properties of rheocast and low-pressure cast A356-T6 alloy[J]. Materials Science and Engineering: A, 2005, 391(1-2): 86-94.

[2] Yi J Z, Lee P D, Lindley T C, et al. Statistical modeling of microstructure and defect population effects on the fatigue performance of cast A356-T6 automotive components[J]. Materials Science and engineering: A, 2006, 432(1-2): 59-68.

[3] Zhu M, Jian Z, Yang G, et al. Effects of T6 heat treatment on the microstructure, tensile properties, and fracture behavior of the modified A356 alloys[J]. Materials & Design (1980-2015), 2012, 36: 243-249.

[4] Yang B C, Chen S F, Song H W, et al. Effects of microstructure coarsening and casting pores on the tensile and fatigue properties of cast A356-T6 aluminum alloy: A comparative investigation[J]. Materials Science and Engineering: A, 2022, 857: 144106.

[5] Chen J, Feng G, Zheng Y, et al. Effect of Cooling Rate on Microstructure Evolution and Mechanical Properties of SCM435 Steel[J]. Metals, 2024, 14(2): 140.

[6] Nguyen V H T, Pessard E, Bellett D, et al. Effects of Shot-Blasting and Induction Hardening on the Fatigue Strength of Hot-Forged Wheel Bearing Hubs[J]. Fatigue & Fracture of Engineering Materials & Structures, 2025.

[7] Zhang P, Wang D, Cheng P, et al. Microstructure, mechanical properties and corrosion behavior of 65Mn tape-steel via electromagnetic induction heating[J]. Materials Today Communications, 2022, 30: 103175.

[8] Chalasani S, Thompson D. Quality improvements in extruded meshes using topologically adaptive generalized elements[J]. International Journal for Numerical Methods in Engineering, 2004, 60(6): 1139-1159.

[9] Budinski K G. Guide to friction, wear and erosion testing[M]. West Conshohocken, PA: ASTM international, 2007.

[10] Draper N R. Applied regression analysis[M]. McGraw-Hill. Inc, 1998.

[11] Sipper M, Moore J H. Conservation machine learning: a case study of random forests[J]. Scientific Reports, 2021, 11(1): 3629.

[12] Chen T, Guestrin C. Xgboost: A scalable tree boosting system[C]//Proceedings of the 22nd acm sigkdd international conference on knowledge discovery and data mining. 2016: 785-794.

[13] Kelleher J D. Deep learning[M]. MIT press, 2019.
