# Supplementary material for: Mechanics-informed risk-aware learning for multiaxial structural reliability with Bayesian calibration
Source: Commun Eng. 2026 Aug 14;5:148. doi: 10.1038/s44172-026-00752-y (PMC13476256; doi:10.1038/s44172-026-00752-y)
Supplement: Supplementary file 3 [file 44172_2026_752_MOESM3_ESM.pdf]

Description of Additional Supplementary Files:

File: Supplementary Data 1

Description: Original data set for Figure 2

File: Supplementary Data 2

Description: Coding for drawing Figure 2
