# Supplementary material for: Mechanics-informed risk-aware learning for multiaxial structural reliability with Bayesian calibration
Source: Commun Eng. 2026 Aug 14;5:148. doi: 10.1038/s44172-026-00752-y (PMC13476256; doi:10.1038/s44172-026-00752-y)
Supplement: Supplementary file 4 [file 44172_2026_752_MOESM5_ESM.pdf]

```
import pandas as pd

import matplotlib.pyplot as plt

import matplotlib as mpl

mpl.rcParams['font.family'] = 'Times New Roman'


# === Read Data ===

df = pd.read_csv("EvaluationMetrics.csv")

df.columns = ['Set', 'Metric'] + list(df.columns[2:])


# === MAPE % ===

for col in df.columns[2:]:

    df[col] = df[col].replace('%', '', regex=True).astype(float)


# === Conversion to Growth Table ===

melted = df.melt(id_vars=['Set', 'Metric'], var_name='Model', value_name='Value')


# === Fixed indicator order (according to CSV&legend) ===

metric_order = ['RMSE', 'MSE', 'MAE', 'MAPE', 'RAE', 'R2', 'RMO']


# === Each indicator independently normalizes bubble size ===

melted['Size'] = 0

for metric in metric_order:

    mask = melted['Metric'] == metric

    vmin, vmax = melted.loc[mask, 'Value'].min(), melted.loc[mask, 'Value'].max()

    melted.loc[mask, 'Size'] =  500*((melted.loc[mask, 'Value'] - vmin) / (vmax -
vmin))**2+2
```

```
# === Color Mapping ===
palette = plt.cm.get_cmap('tab10', len(metric_order))
colors = {m: palette(i) for i, m in enumerate(metric_order)}


# === Drawing fig.2 ===
fig, ax = plt.subplots(figsize=(11, 7))


#   Training upper layer,   Testing lower layer
for idx, set_name in enumerate(['Training set', 'Testing set']):
    subset = melted[melted['Set'] == set_name]
    for metric in metric_order:
        data = subset[subset['Metric'] == metric]
        base_y = metric_order.index(metric)
        flipped_y = len(metric_order)-1 - base_y
        if set_name == 'Training set':
            y_positions = [flipped_y + len(metric_order) for _ in range(len(data))]
        else:
            y_positions = [flipped_y for _ in range(len(data))]
        ax.scatter(data['Model'], y_positions,
                   s=data['Size'], color=colors[metric], alpha=0.7, label=metric if idx
== 0 else "")


# === Background fill ===
xmin, xmax = ax.get_xlim()
ax.axhspan(len(metric_order)-0.5, 2*len(metric_order)-0.5, color='lavender', alpha=0.3,
zorder=0)   # Training
ax.axhspan(-0.5, len(metric_order)-0.5, color='lightblue', alpha=0.3,
zorder=0)                          # Testing
```

```
ax.set_xlim(xmin, xmax)


# === Y-axis label (flip order) ===

y_labels = metric_order[::-1] + metric_order[::-1]

ax.set_yticks(range(len(y_labels)))

ax.set_yticklabels(y_labels)

ax.axhline(y=len(metric_order)-0.5, color='black', linewidth=1)


# === Left annotation Training / Testing ===

ax.text(-1, len(metric_order) + len(metric_order)/2 - 0.5, 'Training set',

        fontsize=16, color='purple', fontweight='bold', va='center', ha='right',
rotation=90)

ax.text(-1, len(metric_order)/2 - 0.5, 'Testing set',

        fontsize=16, color='blue', fontweight='bold', va='center', ha='right',
rotation=90)


# === Unified size legend ===

legend_handles = [plt.Line2D([], [], color=colors[m],

                             marker='o', linestyle='', markersize=8) for m in
metric_order]

first_legend = ax.legend(legend_handles, metric_order,
title='Metrics',title_fontsize=12,fontsize=10,

                         bbox_to_anchor=(1, 1.015), loc='upper left')

ax.add_artist(first_legend)


# === Perfect Prediction External reference ===

perfect_handles = [plt.Line2D([], [], color=colors[m], marker='o',

                              linestyle='', markersize=2 if m != 'R2' else 20)
```

```
                         for m in metric_order]
ax.legend(perfect_handles, metric_order, title='Perfect prediction',title_fontsize=12,fontsize=10,
             bbox_to_anchor=(1, 0.7), loc='upper left')


# === Axis label ===
plt.xlabel('Predictive models', fontsize=16)
plt.xticks(rotation=30, fontsize=12)
plt.yticks(fontsize=12)


plt.tight_layout()
plt.show()


#   High resolution output
fig.savefig("Fig14-Comparative benchmarking of predictive models across training and testing sets.svg", dpi=600, bbox_inches='tight')
```
