# Supplementary material for: Mechanics-informed risk-aware learning for multiaxial structural reliability with Bayesian calibration
Source: Commun Eng. 2026 Aug 14;5:148. doi: 10.1038/s44172-026-00752-y (PMC13476256; doi:10.1038/s44172-026-00752-y)
Supplement: Supplementary file 5 [file 44172_2026_752_MOESM6_ESM.pdf]

nature portfolio

Corresponding author(s): Gaofeng Zhang, Xuanrui Yu
Last updated by author(s): Gaofeng Zhang

Machine Learning Checklist V 1.1

Nature Portfolio wishes to improve the reproducibility of the work that we publish. This form is intended to provide structure for consistency and transparency in reporting of works using or developing Machine Learning models. Some list items might not apply to an individual manuscript, but all fields must be completed for clarity.

1. Availability and reproducibility of Code and Data

Please select all that apply regarding the availability of the data and code used in the study.

☐ Code will be included in a CodeOcean capsule.

☑ The **source code** is included in the submission or available in a public repository:
https://github.com/Peaker99/Risk-ware-learning-with-mechanics-informed-feature-augmentation.git

☐ A **compiled standalone version** of the software is included in the submission or available in a public repository:
URL

☐ A **test dataset** and instructions/scripts for replicating the results are included in the submission or available in a public repository:
URL

☐ A **Readme file** with instructions for installing and running the code is included in the submission or available in a public repository:
URL

☐ The code is made available to reviewers during review.

☐ **Pretrained models** are used in the study and accessible through:
URL

☐ **Pretrained models** are used in the study and are not accessible.

☑ The paper contains information on how to obtain code and data after publication.

2. Datasets

A. All data sources are listed in the paper.

☑ Yes
☐ No

B. The train, test and validation datasets are publicly available, and links/accession numbers have been provided in the manuscript or supplementary materials.

☑ Yes
☐ No

C. We have reported and discussed potential dataset biases in the paper. Where applicable, appropriate mitigation strategies were used.

☑ Yes State where this information can be found in the text

☐ No Explain why this information is not reported/not relevant

D. The data cleaning and preprocessing steps are clearly and fully described, either in text or as a code pipeline.

☑ Yes State where this information can be found in the text

☐ No Explain why this information is not reported/not relevant

E. Instances of combining data from multiple sources are clearly identified, and potential issues mitigated.

☑ Yes State where this information can be found in the text

☐ No Explain why this information is not reported/not relevant

3. Model and training

A. What model architecture is the current model based on? Multiple Linear Regression, Random Forest, XGBoost, B

B. A Model Card is provided[1].

☑ Yes

☐ No

C. The model clearly splits data into different sets for training (model selection), validation (hyperparameter optimization), and testing (final evaluation).

☑ Yes

☐ No

D. The method of data splitting (e.g. random, cluster- or time-based splitting, forward cross-validation) is clearly stated.

☑ Yes State where this information can be found in the text

☐ No Explain why this information is not reported/not relevant

E. The data splitting mimics anticipated real-world applications.

☑ Yes State where this information can be found in the text

☐ No Explain why this information is not reported/not relevant

F. The data splitting procedure has been chosen to avoid data leakage.

☑ Yes State where this information can be found in the text

☐ No Explain why this information is not reported/not relevant

[1] https://huggingface.co/docs/hub/model-cards

G. The interpretability of the model has been studied and clearly validated.

- [x] Yes State where this information can be found in the text
- [ ] No Explain why this information is not reported/not relevant

4. Evaluation

A. The performance metrics used are described and justified in the paper.

- [x] Yes State where this information can be found in the text
- [ ] No Explain why this information is not reported/not relevant

B. Cross-validation of the results is included.

- [ ] Yes
- [x] No

C. Community-accepted benchmark datasets/tasks are used for comparisons.

- [x] Yes State where this information can be found in the text
- [ ] No Explain why this information is not reported/not relevant

D. Baseline comparisons to simple/trivial models (for example, 1-nearest neighbour, random forest, most frequent class) are provided.

- [x] Yes State where this information can be found in the text
- [ ] No Explain why this information is not reported/not relevant

E. Benchmarks with current state-of-the-art are provided.

- [x] Yes State where this information can be found in the text
- [ ] No Explain why this information is not reported/not relevant

F. Ablation experiments are included.

- [x] Yes State where this information can be found in the text
- [ ] No Explain why this information is not reported/not relevant

G. The model has been tested on a fully independent dataset.

- [x] Yes
- [ ] No

5. Computational resources

A. The paper contains information on hardware/computing resources that were used.

- [ ] Yes
- [x] No

B. The paper includes information on the computational costs in terms of computation time, parallelization or carbon footprints estimates.

- [ ] Yes
- [x] No
